# Copper-catalyzed intermolecular C(sp^3^)–H bond functionalization towards the synthesis of tertiary carbamates†
†Dedicated to Professor Iwao Ojima on his 70th birthday.
‡
‡Electronic supplementary information (ESI) available: Experimental procedures, characterization data, and ^1^H and ^13^C NMR charts. See DOI: 10.1039/c5sc00238a



**DOI:** 10.1039/c5sc00238a

**Published:** 2015-03-23

**Authors:** Prasanna Kumara Chikkade, Yoichiro Kuninobu, Motomu Kanai

**Affiliations:** a Graduate School of Pharmaceutical Sciences , The University of Tokyo , 7-3-1 Hongo, Bunkyo-ku , Tokyo 113-0033 , Japan . Email: kuninobu@mol.f.u-tokyo.ac.jp ; Email: kanai@mol.f.u-tokyo.ac.jp; b ERATO (Japan) Science and Technology Agency (JST) , Kanai Life Science Catalysis Project , 7-3-1 Hongo, Bunkyo-ku , Tokyo 113-0033 , Japan

## Abstract

We describe the development of an intermolecular unactivated C(sp^3^)–H bond functionalization towards the direct synthesis of tertiary carbamates. The transformation proceeded using a readily available, abundant first-row transition metal catalyst (copper), and isocyanates as the source of the amide moiety. This is a novel strategy for direct transformation of a variety of unactivated hydrocarbon feedstocks to *N*-alkyl-*N*-aryl and *N*,*N*-dialkyl carbamates without pre-functionalization or installation of a directing group. The reaction had a broad substrate scope with 3° > 2° > 1° site selectivity. The reaction proceeded even on a gram scale, and a corresponding free amine was directly obtained when the reaction was performed at high temperature. Kinetic studies suggested that radical-mediated C(sp^3^)–H bond cleavage was the rate-determining step.

## Abstract 2

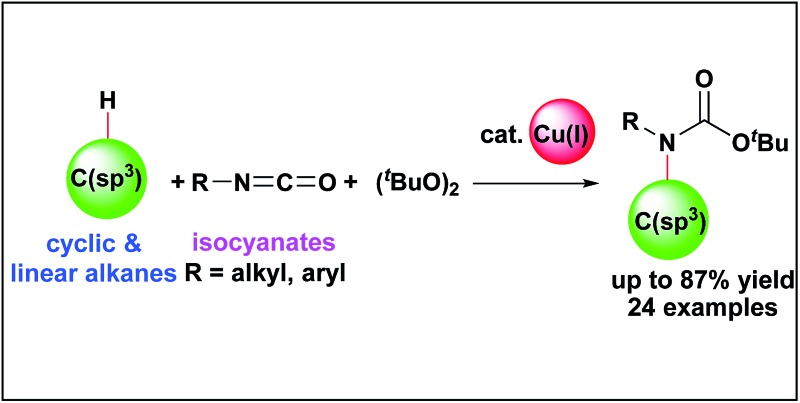
We describe the development of an intermolecular unactivated C(sp^3^)–H bond functionalization towards the direct synthesis of tertiary carbamates.

## Introduction

**Fig. 1:**
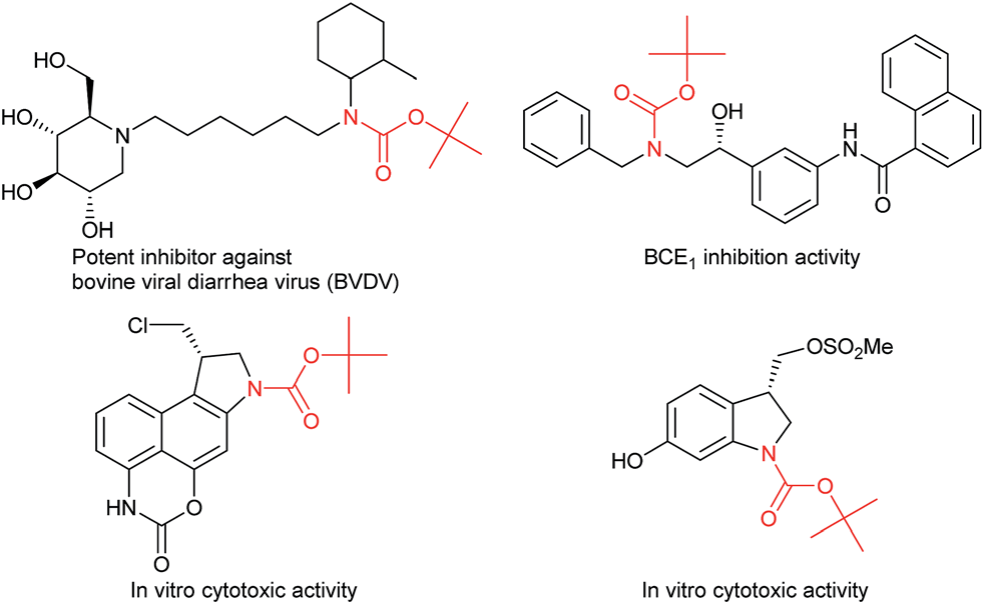
Biologically active compounds with tertiary carbamate motifs.

Carbamates are ubiquitous in nature, and are key functional and structural motifs in a broad range of important compounds, such as pharmaceuticals, agrochemicals, natural products, and functional materials. Molecules with *N*-alkyl-*N*-aryl and *N*,*N*-dialkyl motifs exhibit various biological properties (Fig. 1).^1,2^ The biological significance of carbamates has inspired the development of novel and efficient carbamation reactions.

**Scheme 1:**
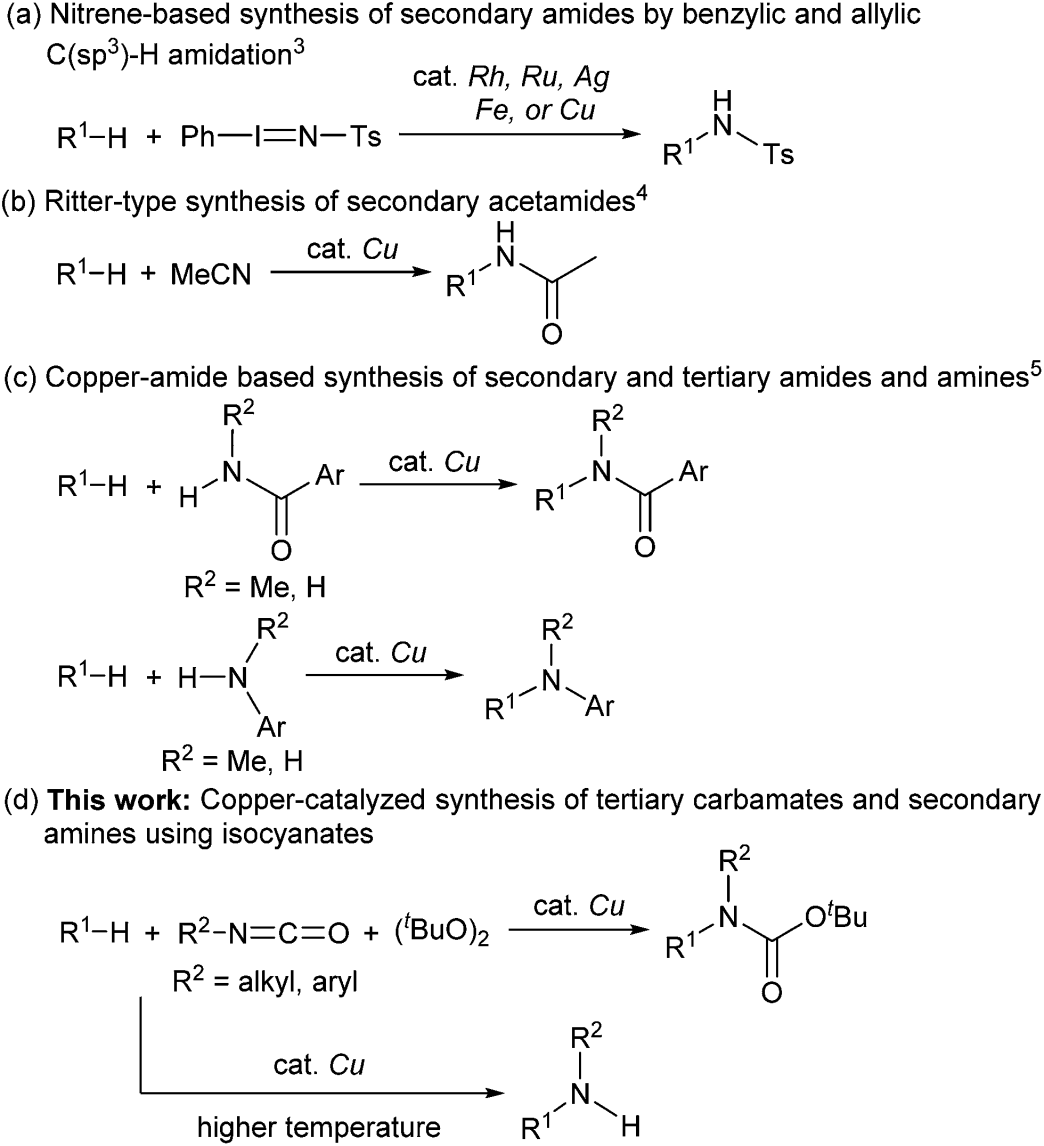
Transition metal-catalyzed C(sp^3^)–H bond transformations to construct C(sp^3^)–N bonds.

Catalytic C(sp^3^)–H amidation, amination, and carbamation of inert hydrocarbons is a challenging and attractive strategy for preparing nitrogen-containing compounds. Reactions such as these that do not require typical functional group manipulations have enormous economic benefits. The notable transformation of a C–H bond to a C–N bond occurs in nitrene chemistry (Scheme 1, route a).^3^ However, intermolecular amidations using nitrenes are limited to reactions which produce secondary amides and functionalize benzylic or allylic positions. A more efficient copper-catalyzed Ritter-type C–H amidation was explored using a fluorine-based oxidant and acetonitrile as the nitrogen source (Scheme 1, route b); however, this reaction was limited to the synthesis of *N*-monoalkyl acetamides.^4^ Hartwig’s and Warren's copper-amide-based strategies constitute powerful synthetic methods (Scheme 1, route c); albeit, *N*-alkyl-*N*-aryl and *N*,*N*-dialkyl amides (tertiary amides) were obtained in low yields.^5^ Some other intermolecular and intramolecular C–H amination reactions with or without the use of metal catalysts have also been reported.^6^

Despite remarkable progress in C–H amidation and amination reactions, the formation of tertiary carbamates from inert hydrocarbons remains underdeveloped and the synthesis of *N*-alkyl-*N*-aryl or *N*,*N*-dialkyl tertiary carbamates remains a challenge. Although several reagents are effective as nitrogen atom sources in amidation and amination reactions, isocyanates have not been used to synthesize carbamates. Herein we report a novel and efficient unactivated C(sp^3^)–H bond functionalization that produces *N*-alkyl-*N*-aryl and *N*,*N*-dialkyl tertiary carbamates and the corresponding secondary amines using a copper catalyst and isocyanates as carbamation reagents (Scheme 1, route d).

## Results and discussion

First, we investigated transition metal complexes and oxidants for the reaction between cyclohexane (**1a**) and phenyl isocyanate (**2a**) (Table 1). In the presence of a copper(i) iodide–phenanthroline complex (10 mol%) and oxidant **3** in benzene solvent, *tert*-butyl *N*-cyclohexyl-*N*-phenylcarbamate (**4a**) was obtained in 30% yield (entry 1). Other copper salts were comparably active regardless of their counterions and oxidation states (entries 2–4). The cationic copper complex [Cu(NCMe)_4_]BF_4_ proved to be the best catalyst, giving **4a** in 47% yield (entry 5). Other peroxides, such as *tert*-butyl hydroperoxide (TBHP) and *tert*-butyl perbenzoate (TBPB), did not produce **4a**, and di-*tert*-amyl peroxide (DTAP) had lower reactivity (entries 6–8). Further screening of bipyridyl-type and phenanthrolyl-type ligands revealed that neocuproine was the best ligand, affording the desired product **4a** in 67% yield (entries 9–13). Extensive evaluation revealed that a reaction in the presence of 5 mol% of [Cu(NCMe)_4_]BF_4_ and 2.5 equiv. of oxidant **3** in trifluorotoluene as a co-solvent gave **4a** in 75% yield (entry 14). Other transition metal salts, however, such as Fe(OAc)_2_, Co(OAc)_2_, Ni(OAc)_2_·4H_2_O, and AgOAc, did not promote the carbamation reaction. In addition, the reaction in the absence of a ligand led to low yield (32%).

**Table 1:** Development of optimized conditions for *N*-phenylcarbamation of cyclohexane^*a*^

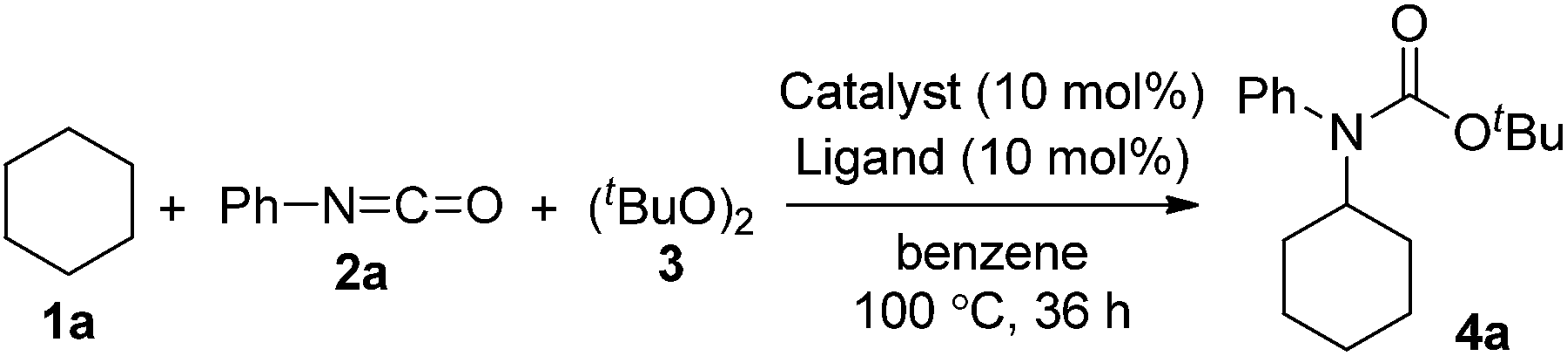			
Entry	Catalyst	Ligand	Yield^*b*^ (%)
1	CuI	1,10-Phen	30
2	CuCl	1,10-Phen	36
3	CuCl_2_	1,10-Phen	35
4	CuOAc	1,10-Phen	32
5	[Cu(NCMe)_4_]BF_4_	1,10-Phen	47
6^*c*^	[Cu(NCMe)_4_]BF_4_	1,10-Phen	0
7^*d*^	[Cu(NCMe)_4_]BF_4_	1,10-Phen	0
8^*e*^	[Cu(NCMe)_4_]BF_4_	1,10-Phen	20
9	[Cu(NCMe)_4_]BF_4_	(MeO)_2_phen	60
10	[Cu(NCMe)_4_]BF_4_	Cl_2_phen	51
11	[Cu(NCMe)_4_]BF_4_	^*t*^Bu_2_bipy	55
12	[Cu(NCMe)_4_]BF_4_	Bathocuproine	53
13	[Cu(NCMe)_4_]BF_4_	Neocuproine	67
14^*f*^	[Cu(NCMe)_4_]BF_4_	Neocuproine	75

^*a*^Reaction conditions: **1a** (5.00 mmol), **2a** (0.500 mmol), **3** (1.00 mmol), catalyst (0.0500 mmol), ligand (0.0500 mmol), C_6_H_6_ (1.0 mL), 100 °C, 36 h.

^*b*^ ^1^H NMR yield using 1,1,2,2-tetrachloroethane as an internal standard.

^*c*^TBHP (2.0 equiv.) was used as an oxidant.

^*d*^TBPB (2.0 equiv.) was used as an oxidant.

^*e*^DTAP (2.0 equiv.) was used as an oxidant.

^*f*^Reaction in trifluorotoluene, Cu catalyst (5.0 mol%) and ligand (5.0 mol%), **3** (2.5 equiv.), 100 °C, 24 h. 1,10-Phen = 1,10-phenanthroline, (MeO)_2_phen = 4,7-dimethoxyphenanthroline, Cl_2_phen = 4,7-dichlorophenanthroline, ^*t*^Bu_2_phen = 4,7-di(*tert*-butyl)phenanthroline, bathocuproine = 2,9-dimethyl-4,7-diphenyl-1,10-phenanthroline, neocuproine = 2,9-dimethyl-1,10-phenanthroline.

The substrate scope of isocyanates was investigated under the above optimal reaction conditions (Table 2). A variety of aryl and alkyl isocyanates were tested in cyclohexane (**1a**) or in a mixture of **1a**/trifluorotoluene. Aryl isocyanates possessing a halogen atom, such as a fluorine, chlorine, or bromine atom, afforded the desired products **4b–e** in excellent yields. Aryl isocyanates containing a trifluoromethyl group underwent carbamation of cyclohexane, affording the corresponding carbamates **4f** and **4g** in optimum yields. The carbamation reaction also proceeded from aryl isocyanates **4h–j** containing an electron-donating group, such as a methoxy, methyl, or *n*-butyl group; however, the isolated yields of the products were slightly lower than those of aryl isocyanates with an electron-withdrawing group. Aryl isocyanates containing an acetyl, methoxycarbonyl, or cyano group were also effective, and the corresponding tertiary carbamates **4k–m** were obtained in good yields. The carbamation reaction was next extended to more challenging substrates, alkyl isocyanates. Notably, reactions of **1a** also occurred with alkyl isocyanates, such as *n*-butyl, cyclopentyl, and cyclohexylisocyanates, giving the corresponding *N*,*N*-dialkyl carbamates (**4n–p**).

**Table 2:** C(sp^3^)–H carbamation of cyclohexane with various isocyanates^*a*^ ^,^^*b*^

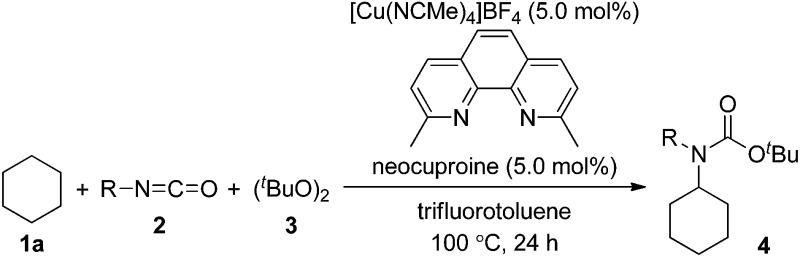
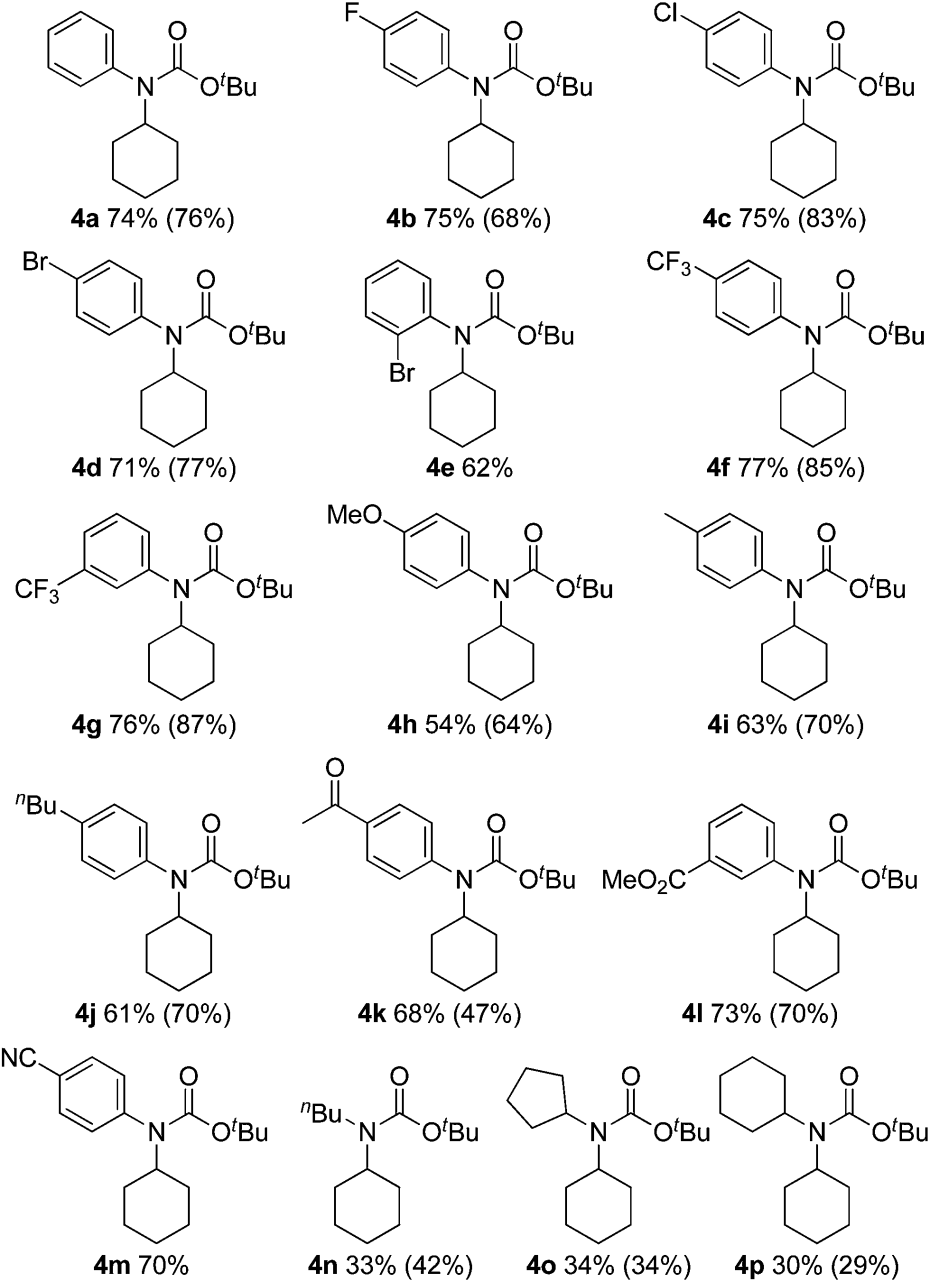

^*a*^Reaction conditions: **1a** (5.00 mmol), **2** (0.500 mmol), **3** (1.25 mmol), [Cu(NCMe)_4_]BF_4_ (0.0250 mmol), neocuproine (0.0250 mmol), trifluorotoluene (0.5 mL), 100 °C, 24 h.

^*b*^Yields in parentheses were obtained in cyclohexane (1.6 mL, 15.0 mmol).

We next evaluated C(sp^3^)–H carbamation of other cyclic and linear unactivated alkanes (Table 3). C(sp^3^)–H carbamation of cycloalkanes such as cyclopentane, cycloheptane, and cyclooctane proceeded well, giving the corresponding tertiary carbamates **4q–s** in good yields. C(sp^3^)–H carbamation of norbornane proceeded with single-site selectivity and gave a mixture of stereoisomers **4t** in 46% yield (*exo* : *endo* = 6 : 1). The reaction of adamantane with phenyl isocyanate (**2a**) afforded a regioisomeric mixture (**4ua** and **4ub**) in 42% yield (**4ua** : **4ub** = 1.7 : 1). In the case of toluene, benzylic C(sp^3^)–H amidation proceeded and **4v** was obtained in 44% yield. Ethylbenzene showed exclusive site selectivity, and furnished only a single carbamation product **4w** in good yield. The reaction of **2a** with a linear alkane (*n*-hexane), C(sp^3^)–H carbamation, proceeded nicely and afforded a mixture of carbamates **4xa**, **4xb**, and **4xc** (3.2 : 1.4 : 1) in good yield. This is a highly valuable transformation of hydrocarbon feedstocks to tertiary carbamates.

**Table 3:** C(sp^3^)–H aryl-carbamation of various alkanes^*a*^

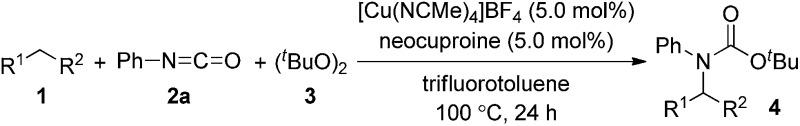
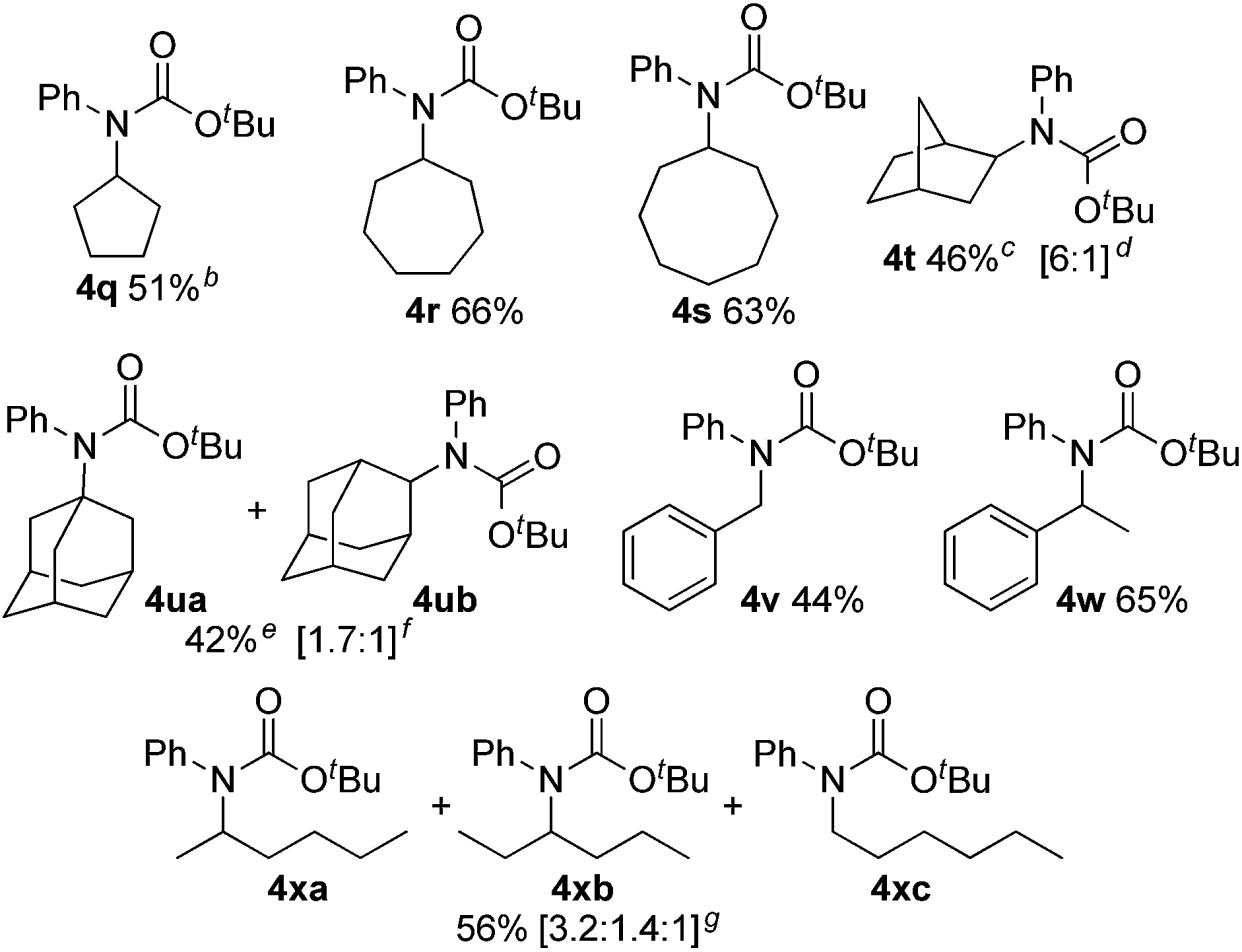

^*a*^Reaction conditions: **1** (5.00 mmol), **2a** (0.500 mmol), **3** (1.25 mmol), [Cu(NCMe)_4_]BF_4_ (0.0250 mmol), neocuproine (0.0250 mmol), trifluorotoluene (1.0 mL), 100 °C, 24 h.

^*b*^Cyclopentane (1.4 mL, 30 equiv.) was used.

^*c*^Trifluorotoluene (1.5 mL) was used.

^*d*^Ratio of *exo*- to *endo*-products.

^*e*^Trifluorotoluene (2.0 mL) was used.

^*f*^Ratio of **4ua** to **4ub**.

^*g*^Ratio of **4xa** to **4xb** to **4xc**.

**Scheme 2:**
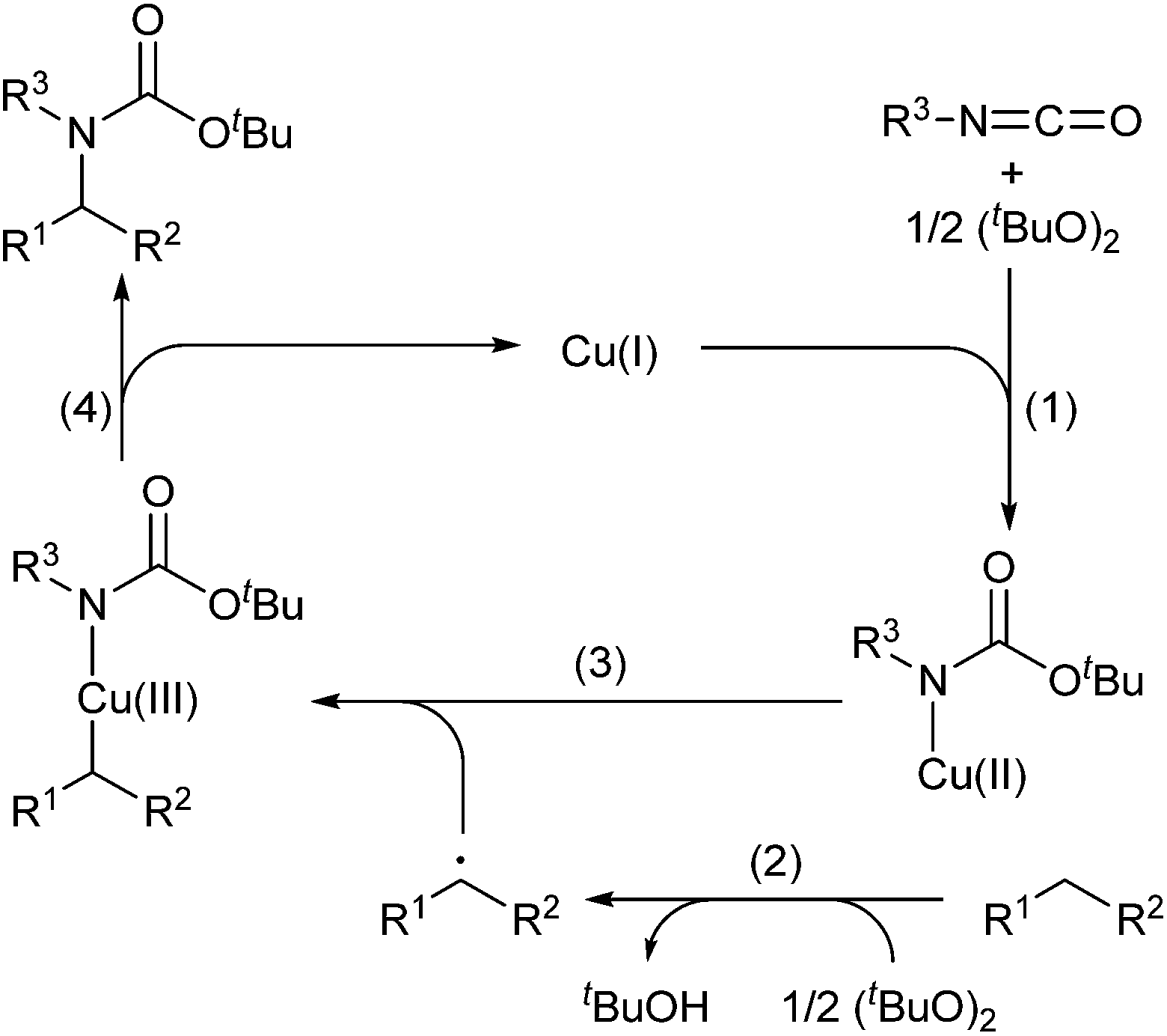
Proposed catalytic cycle for C(sp^3^)–H carbamation of alkanes.

A proposed reaction mechanism is summarized in Scheme 2: (1) the copper(i) species-catalyzed^7^ or thermal homolytic cleavage of a peroxide generates *tert*-butoxy radical, which reacts with an isocyanate and sequential oxidation of the copper(i) species to give Cu(ii)–amide species; (2) abstraction of a hydrogen atom from the C(sp^3^)–H bond of an alkane by *tert*-butoxy radical generates an alkyl radical with the release of ^*t*^BuOH;^8^ (3) combination of the alkyl radical with Cu(ii)–amide species produces a Cu(iii) intermediate with alkyl and amide ligands;^8*a*^ and (4) reductive elimination of the Cu(iii) species affords a tertiary carbamate and regenerates the Cu(i) species.

**Scheme 3:**
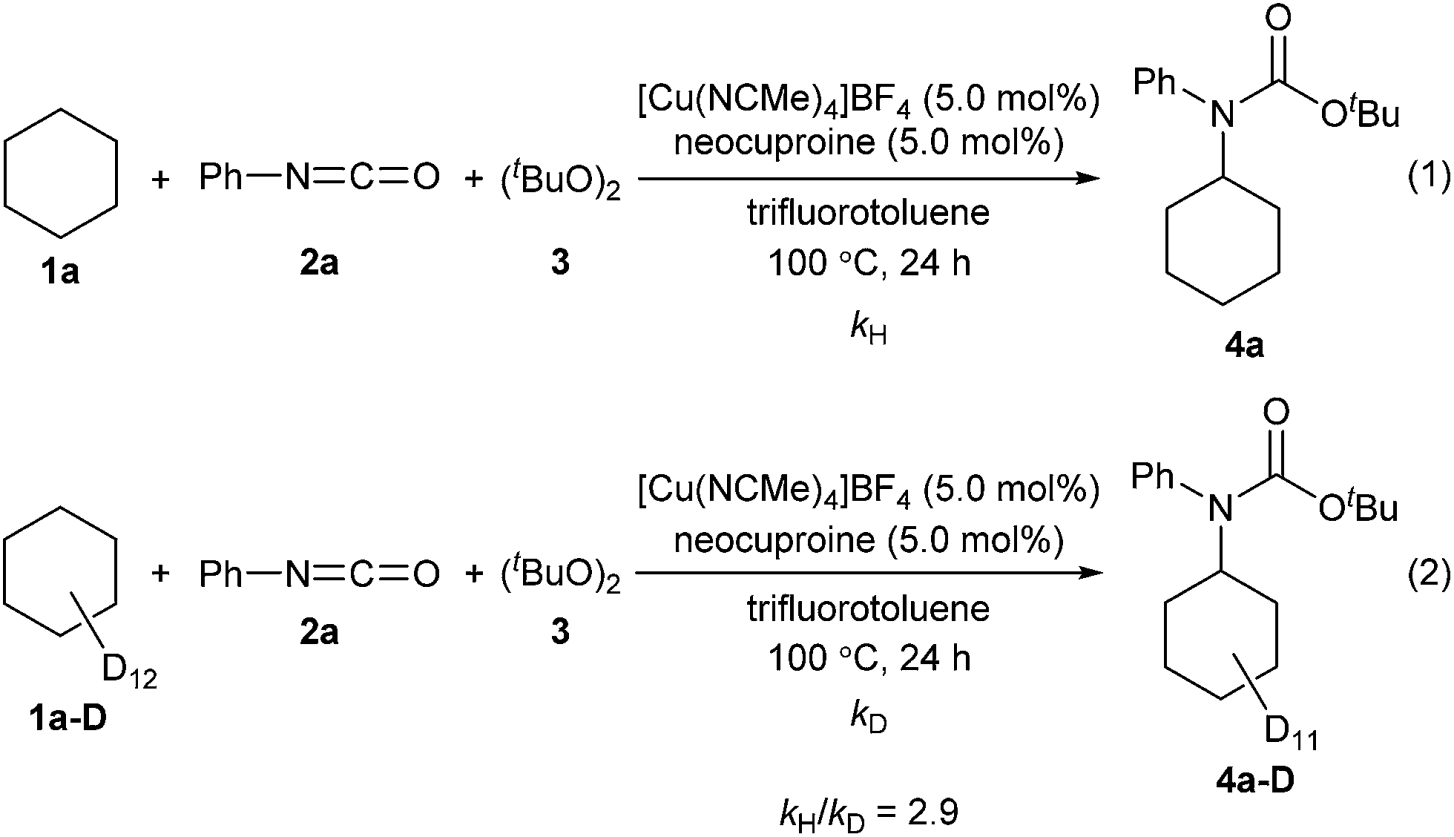
Kinetic isotopic effect studies.

Next, kinetic isotopic effect studies with separate kinetic experiments were performed to gain insight into the rate-determining step for the amidation of cyclohexane (**1a**). Significant kinetic isotopic effects were observed (*k*_H_/*k*_D_ = 2.9), suggesting that C–H bond cleavage was the rate-determining step of the reaction (Scheme 3, for details, see ESI‡).

**Scheme 4:**
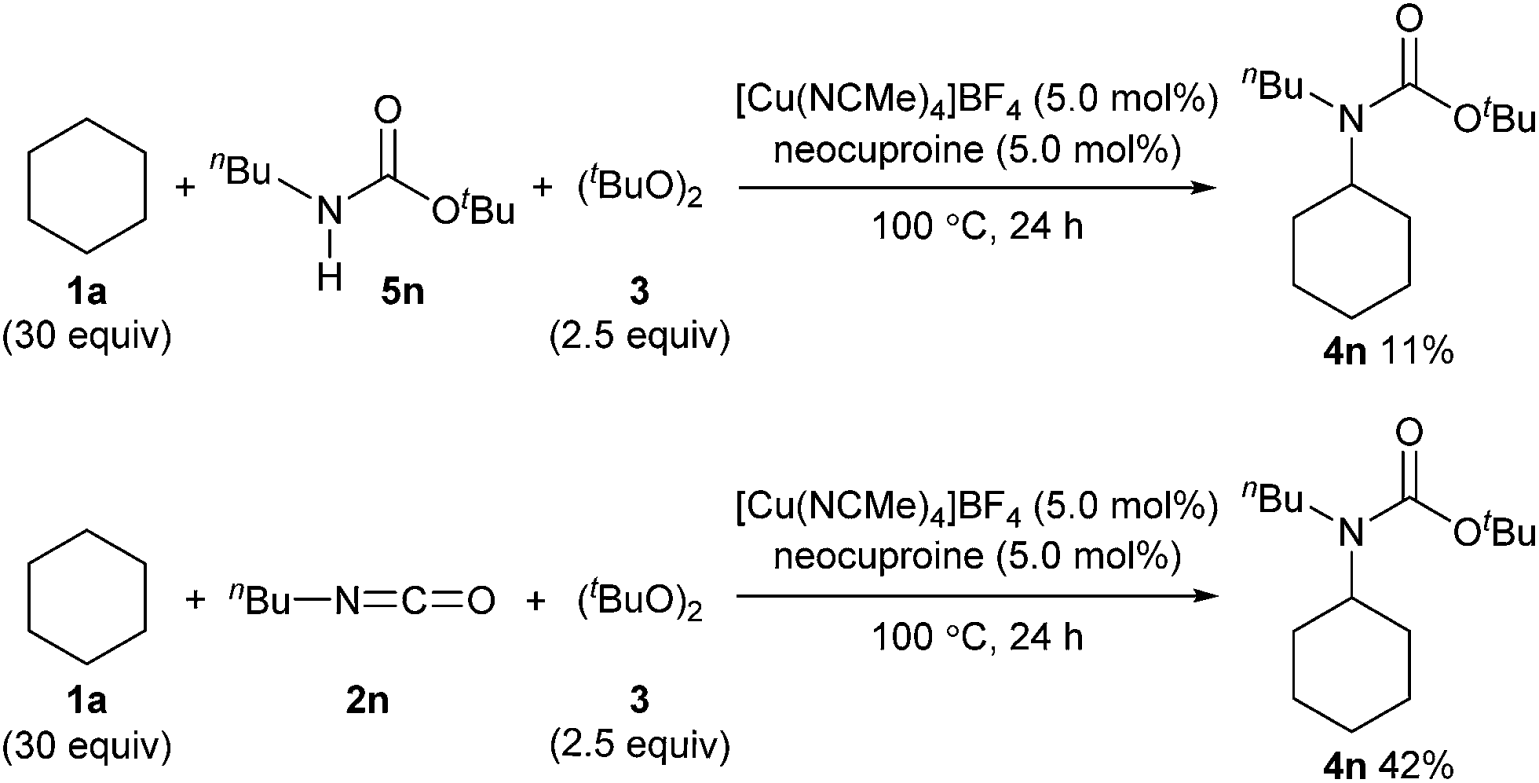
Evaluation of reactivity difference between secondary carbamates and isocyanates.

We also examined the reactivity difference between carbamate **5n** and isocyanate **2n** in the carbamation reaction (Scheme 4). Treatment of *n*-butyl carbamate **5n** with cyclohexane (**1a**) under the optimized reaction conditions afforded **4n** in only 11% yield. On the other hand, *n*-butyl isocyanate (**2n**) gave **4n** in 42% yield under similar conditions. These results indicated that isocyanates are better carbamation reagents than secondary carbamates^5*d*^ under the optimized conditions.

**Scheme 5:**
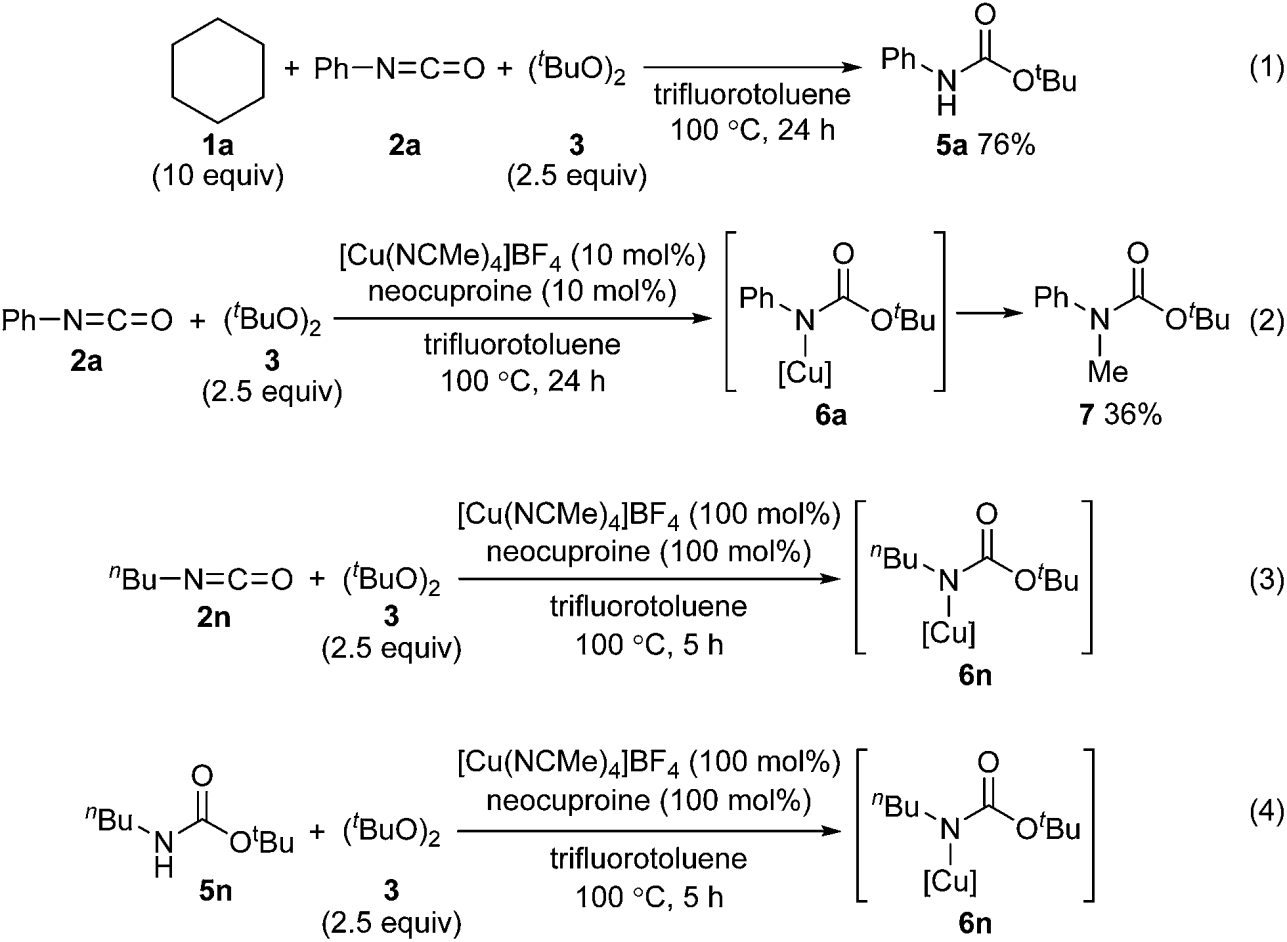
Several experiments performed to understand the reaction mechanism: (1) a reaction between cyclohexane (**1a**), isocyanate **2a**, and peroxide **3** without using a copper catalyst; (2) a reaction between isocyanate **2a** and peroxide **3** without addition of cyclohexane (**1a**); (3) *in situ*-FT-IR study of a reaction between [Cu(NCMe)_4_]BF_4_, isocyanate **2n**, and peroxide **3**; (4) *in situ*-FT-IR study of a reaction between [Cu(NCMe)_4_]BF_4_, carbamate **5n**, and peroxide **3**.

To address the reactivity difference between isocyanates and secondary carbamates, and to gain further support for the proposed reaction mechanism in Scheme 2, we performed several reactions as follows (Scheme 5): (1) A reaction of phenyl isocyanate (**2a**) with cyclohexane (**1a**) under the optimized conditions without using a copper catalyst was performed. As a result, carbamate **5a** was obtained in 76% yield, and tertiary carbamate **4a** was not formed at all (Scheme 5(1)). This result showed that the copper catalyst was crucial for coupling between isocyanates and alkanes. (2) A reaction of phenyl isocyanate (**2a**) in the absence of **1a** under the optimized conditions afforded carbamate **7** in 36% yield (Scheme 5(2)). A methyl radical was generated through β-methyl elimination of a *tert*-butoxy radical, and sequential coupling with an *in situ* generated copper(ii)–carbamate species (**6a**) would lead to the formation of carbamate **7**. (3) A reaction of *n*-butyl isocyanate (**2n**) with di-*tert*-butyl peroxide (**3**) in the presence of a stoichiometric amount of a copper complex was conducted in trifluorotoluene, and the course of the reaction was monitored by *in situ*-FT-IR (Scheme 5(3), see ESI‡ for details). As a result, an absorption band at 2266 cm^–1^ (assigned for the carbonyl group of isocyanate **2n**) started to disappear after 1.4 h (induction period), and a new absorption band, possibly assigned to the carbonyl groups of copper complex **6n**, appeared at 1725 cm^–1^. The maximum absorbance intensity at 1725 cm^–1^ was 0.063 (A.U.). (4) Under similar conditions, the progress of a reaction between *n*-butyl carbamate **5n** and di-*tert*-butyl peroxide (**3**) and the formation of copper complex **6n** was monitored by *in situ*-FT-IR (Scheme 5(4), see ESI‡ for details). An absorbance band for the carbonyl group of carbamate **5n** was observed at 1720 cm^–1^, and an absorbance band at 1725 cm^–1^ corresponding to **6n** started to appear after 3.9 h (induction period). The maximum absorbance intensity at 1725 cm^–1^ was 0.040 (A.U., the absorbance intensity of **6n** must be less than 0.040 due to overlapping with an absorbance of **5n**). The differences in the induction period and absorbance intensity of the bands at 1725 cm^–1^ for reactions (3) and (4) correlated well with the result that *n*-butyl isocyanate (**2n**) was more reactive compared with *n*-butyl carbamate **5n**. In addition, the reaction of cyclohexane (**1a**) with phenylisocyanate (**2a**) was almost completely inhibited by 2,2,6,6-tetramethylpiperidine 1-oxyl (TEMPO, 2 equivalents) under the optimized conditions. This result indicated that a radical pathway is involved as a key step in the catalytic cycle.

**Scheme 6:**
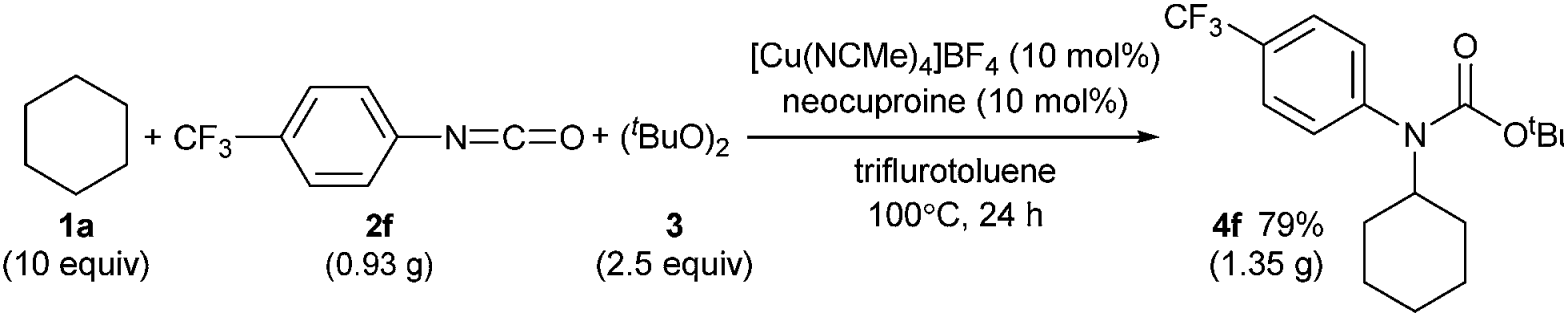
Gram scale synthesis of *tert*-butyl *N*-cyclohexyl-*N*-(4-trifluromethylphenyl)carbamate (**4f**).

C(sp^3^)–H carbamation could also be performed on a gram scale by treating cyclohexane (**1a**) and *p*-trifluoromethylphenyl isocyanate (**2f**) with a copper catalyst in trifluorotoluene solvent (Scheme 6, see also the ESI‡). As a result, **4f** was obtained in 79% yield (1.35 g), which was comparable to the yield of **4f** on a smaller scale (77% yield, 132 mg, Table 2).

**Scheme 7:**
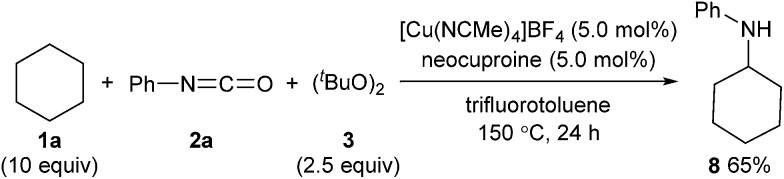
One-pot direct synthesis of a secondary amine from an alkane.

The developed method can be further extended to the direct synthesis of valuable amines from alkanes in combination with thermal cleavage of the *tert*-butoxycarbonyl group at a higher temperature (Scheme 5). The reaction of cyclohexane (**1a**) with phenyl isocyanate (**2a**) and (^*t*^BuO)_2_ (**3**) at 150 °C gave *N*-cyclohexylaniline (**8**) in 65% yield (Scheme 7).

## Conclusions

In summary, we developed a copper-catalyzed, non-nitrene-based C(sp^3^)–H carbamation reaction. This new C(sp^3^)–H to C(sp^3^)–N bond transformation proceeded from unactivated alkanes and isocyanates. Although there are few examples of copper-catalyzed amidation or amination to afford secondary amides or amines, this novel protocol allowed us to obtain tertiary carbamates directly from hydrocarbon feedstocks. The reaction had a broad substrate scope, and the observed site selectivity was 3° > 2° > 1°. The reaction proceeded smoothly even on a gram scale, and a higher temperature directly produced the corresponding free amine without the addition of an acid. Kinetic studies suggested that radical mediated C(sp^3^)–H bond cleavage was the rate-limiting step. The copper-catalyzed C(sp^3^)–H carbamation reaction will be useful for rapid construction of synthetically useful and pharmaceutically valuable molecules bearing *N*-alkyl-*N*-aryl or *N*,*N*-dialkyl tertiary carbamate moieties.

## Supplementary Material

Supplementary information
