# Supplementary material for: Copper-catalyzed intermolecular C(sp3)–H bond functionalization towards the synthesis of tertiary carbamates
Source: Chem Sci. 2015 Mar 23;6(5):3195–200. doi: 10.1039/c5sc00238a (PMC5657409; doi:10.1039/c5sc00238a)
Supplement: Supplementary file 1 [file SC-006-C5SC00238A-s001.pdf]

Electronic Supplementary Material (ESI) for Chemical Science.
This journal is © The Royal Society of Chemistry 2015

Supporting Information

Copper-Catalyzed Intermolecular C(sp^3)-H Bond Functionalization Towards the Synthesis of Tertiary Carbamates **

Prasanna Kumara Chikkade,[†] Yoichiro Kuninobu*[†,‡] and Motomu Kanai*[†,‡]

[†]Graduate School of Pharmaceutical Sciences, The University of Tokyo
7-3-1 Hongo, Bunkyo-ku, Tokyo 113-0033, Japan
[‡]ERATO, Japan Science and Technology Agency (JST), Kanai Life Science Catalysis Project, 7-3-1 Hongo, Bunkyo-ku, Tokyo113-0033, Japan

E-mail: kuninobu@mol.f.u-tokyo.ac.jp; kanai@mol.f.u-tokyo.ac.jp

Table of Contents

Contents	Page No.
1. General	S2
2. Biologically Active Compounds Containing Tertiary Carbamate Motifs	S3
3. Optimization of Reaction Conditions	S4
4. General Procedure	S15
5. Characterization Data	S16
6. Evaluation of Reactivity Difference between Carbamate and Isocyanate	S23
7. Several Experiments for Understanding the Reaction Mechanism and Plausible Reaction Mechanism	S24
8. Gram Scale Experiment	S30
9. Kinetic Isotopic Effect (KIE) Experiments	S31
10. Copper-Catalyzed Amination of Cyclohexane	S33
11. ^{1}H and ^{13}C NMR Spectra	S34

1. General

Unless otherwise noted, all reactions were performed in a flame-dried 10 mL screw cap reaction tubes with Teflon-coated magnetic stirring bar. Air- and moisture-sensitive liquids were transferred via a gas-tight syringe and stainless steel needle under argon atmosphere. All work-up and purification procedures were carried out with reagent-grade solvents in air at ambient temperature. Column chromatographic purifications were performed with silica gel Merck 60 (230-400 mesh ASTM).

^{1}H and ^{13}C NMR spectra of isolated compounds were recorded on JEOL ECX500 (500 MHz for ^{1}H NMR and 125 MHz for ^{13}C NMR) spectrometer. Chemical shifts were reported in parts per million (ppm) in the scale relative to the solvent used as an internal reference for ^{1}H (δ = 7.26 ppm for $CDCl_3$) and ^{13}C NMR (δ = 77.0 ppm for $CDCl_3$). Data are reported as follows: chemical shift, multiplicity (s = singlet, d = doublet, dd = doublet of doublet, td = triplet of doublet, m = multiplet), coupling constants (Hz), and integration. Infrared (IR) spectra were recorded on a JASCO FT/IR 410 Fourier transform infrared spectrophotometer. ESI-mass spectra were measured on JEOL JMS-T100LC Accutof spectrometer for HRMS. Recycling preparative HPLC (Gel permeation chromatography) (Japan Analytical Industry Co., Ltd.) LC9210NEXT equipped with JAIGEL-1H and JAIGEL-2H columns and chloroform as an eluent were used to purify some of the compounds. *In-situ* FTIR experiments were conducted using ReactIR 4000 (Mettler Toledo AutoChem ReactIR) instrument.

Isocyanates (**2a**, **2b**, **2c**, **2d**, **2e**, **2f**, **2g**, **2h**, **2i**, **2j**, **2k**, **2l**, **2m**, and **2p**), alkanes (**1a, 1q**, **1r**, **1s**, **1t**, **1u**, **1v**, **1w**, and **1x**), oxidants and solvents were purchased from Aldrich Chemical Company (Aldrich), Wako Pure Chemical Industries (Wako), Ltd., or Tokyo Chemical Industry Co., Ltd. (TCI), and were used as received without any further purification. Tetrakis(acetonitrile)copper(I) tetrafluroborate and neocuproine (2,9-dimethyl-1,10-phenanthroline) were purchased from Aldrich. Butyl *tert*-butyl carbamate (**5n**) was synthesized according to the reported literature procedure[1] and all the spectral data for the obtained carbamate **5n** were in accord with the reported data. All solvents and alkanes were stored over 4Å molecular sieves before use.

[1] (*a*) K. S. Kumar, J. Iqbal and M. Pal, *Tetrahedron Lett.*, 2009, **50**, 6244; (*b*) L. R. Steffel, T. J. Cashman, M. H. Reutershan and B. R. Linton, *J. Am. Chem. Soc.*, 2007, **129**, 12956.

2. Biologically Active Compounds Containing Tertiary Carbamate Motifs

Organic compounds having *N*-alkyl-*N*-aryl and *N*,*N*-dialkyl carbamate motifs show wide variety of biological properties. Some of the representative examples[2] and their biological properties are shown in the Figure S1. Compound **C-1** having an *N*,*N*-dialkyl carbamate motif displays potent inhibitor activity against bovine viral diarrehea virus (BVDV, EC_{50} 0.4 mM), and **C-2** displays BCE_1 inhibition activity (IC_{50} 4.9 mM). Compounds **C-3** and **C-4** display *in-vitro* cytotoxic activities (IC_{50} L_{1210} 4900 nm and IC_{50} L_{1210} 0.04 mg/mL, respectively).

The biological significance of the compounds containing *N*-alkyl-*N*-aryl and *N*,*N*-dialkyl carbamate motifs have attracted many synthetic efforts to develop greener C-N bond forming reactions. In this regard, we have developed conceptually new and greener approach for the preparation of tertiary carbamates (*N*-alkyl-*N*-aryl and *N*,*N*-dialkyl carbamates) from hydrocarbon feedstocks by using cheaply available first-row transition metal catalysis. Reaction optimization studies were described below.

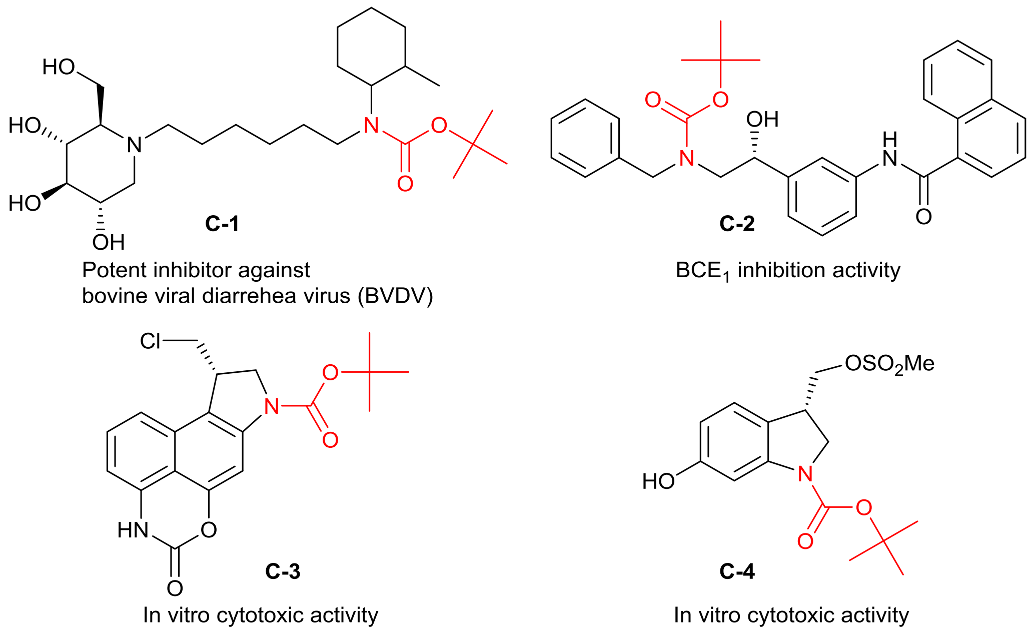

Figure S1. Biologically active compounds with tertiary carbamate motifs.

---

[2] (*a*) D. L. Boger, H. Zarrinmayeh, S. A. Munk, P. A. Kitos, O. Suntornwat, *Proc. Natl. Acad. Sci. U. S. A.*, 1991, **88**, 1431; (*b*) A. L. Wolfe, K. K. Duncan, N. K. Parelkar, S. J. Weir, G. A. Vielhauer, D. L. Boger, *J. Med. Chem.*, 2012, **55**, 5878; (*c*) Y. Du, H. Ye, T. Gill, L. Wang, F. Guo, A. Cuconati, J.-T. Guo, T. M. Block, X. Xu, *Bioorg. Med. Chem. Lett.*, 2013, **23**, 2172; (*d*) S. Butini, E. Gabellieri, M. Brindisi, S. Giovani, S. Maramai, G. Kshirsagar, S. Brogi, V. L. Pietra, M. Giustiniano, L. Marinelli, E. Novellino, G. Campiani, A. Cappelli, S. Gemma, *Eur. J. Org. Chem.*, 2013, **70**, 233.

3. Optimization of Reaction Conditions

3-1. Screening of Metal Sources

In a nitrogen-filled glove box, a flame-dried 10 mL screw cap test tube with magnetic stir-bar was charged with a metal salt (0.050 mmol, 10 mol%) and 1,10-phenanthroline (9.0 mg, 0.050 mmol, 10 mol%). The test tube was removed from the glove box, and cyclohexane (**1a**, 0.54 mL, 5.00 mmol, 10.0 equiv), phenyl isocyanate (**2a**, 59.6 mg, 54.3 µL, 0.500 mmol), di-*tert*-butylperoxide (**3**, 146 mg, 0.18 mL, 1.00 mmol, 2.0 equiv), and benzene (1.0 mL) were added under argon atmosphere. The test tube was placed in a preheated oil bath and the mixture was stirred vigorously at 100 °C for 36 h. Cooled to room temperature, the reaction mixture was passed through a short pad of silica gel with the aid of dichloromethane, and concentrated. ^{1}H NMR using 1,1,2,2-tetrachloroethane as an internal standard quantified the yield of **4a**.

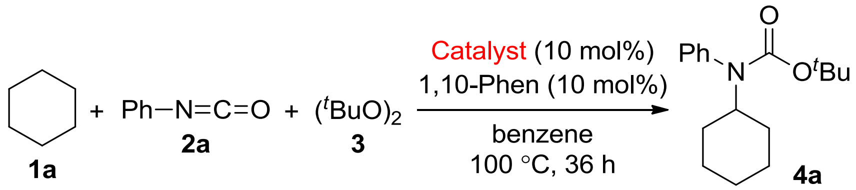

Entry	Metal Source (MX)	Yield (%)[a]
1	-	0
2	CuI	30
3	CuCl	36
4	CuSCN	34
5	CuBr	34
6	$CuCl_2$	35
7	CuCN	27
8	$Cu(OAc)_2$	10
9	$[Cu(NCMe)_2]OTf$	36
10	$Co(OAc)_2$	0
11	$Fe(OAc)_2$	0
12	AgOAc	0
13	$Ni(OAc)_2 \cdot 4H_2O$	0
14	CuOAc	32
**15**	**$[Cu(NCMe)_4]BF_4$**	**47**
16	$[Cu(NCMe)_4]PF_6$	47

[a]Yield was determined by ^{1}H NMR spectrum using 1,1,2,2-tetrachloroethane as an internal standard.

Tetrakis(acetonitrile)copper (I) tetrafluroborate $[Cu(NCMe)_4]BF_4$ was proved to be the best metal source for carbamation of cyclohexane.

3-2. Screening of Oxidants

In a nitrogen-filled glove box, a flame-dried 10 mL screw cap test tube with a magnetic stir-bar was charged with tetrakis(acetonitrile)copper(I) tetrafluoroborate $[Cu(NCMe)_4]BF_4$ (15.7 mg, 0.0500 mmol, 10 mol%) and 1,10-phenanthroline (9.0 mg, 0.0500 mmol, 10 mol%). The test tube was removed from the glove box, and cyclohexane (**1a**, 0.54 mL, 5.00 mmol, 10.0 equiv), phenyl isocyanate (**2a**, 59.6 mg, 54.3 μL, 0.500 mmol), oxidant (1.00 mmol, 2.0 equiv), and benzene (1.0 mL) were added under argon atmosphere. The test tube was placed in preheated oil bath and the mixture was stirred vigorously at 100 °C for 36 h. Cooled to room temperature, the reaction mixture was passed through a short pad of silica gel with the aid of dichloromethane, and concentrated. ^{1}H NMR using 1,1,2,2-tetrachloroethane as an internal standard quantified the yield of **4a**.

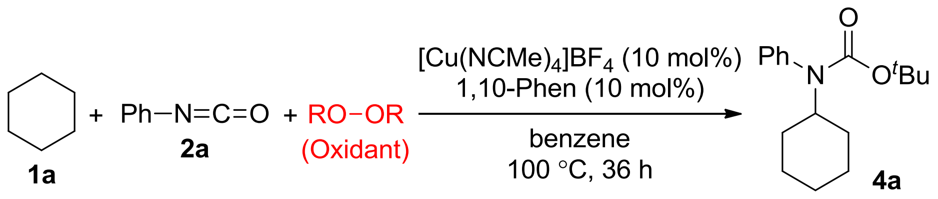

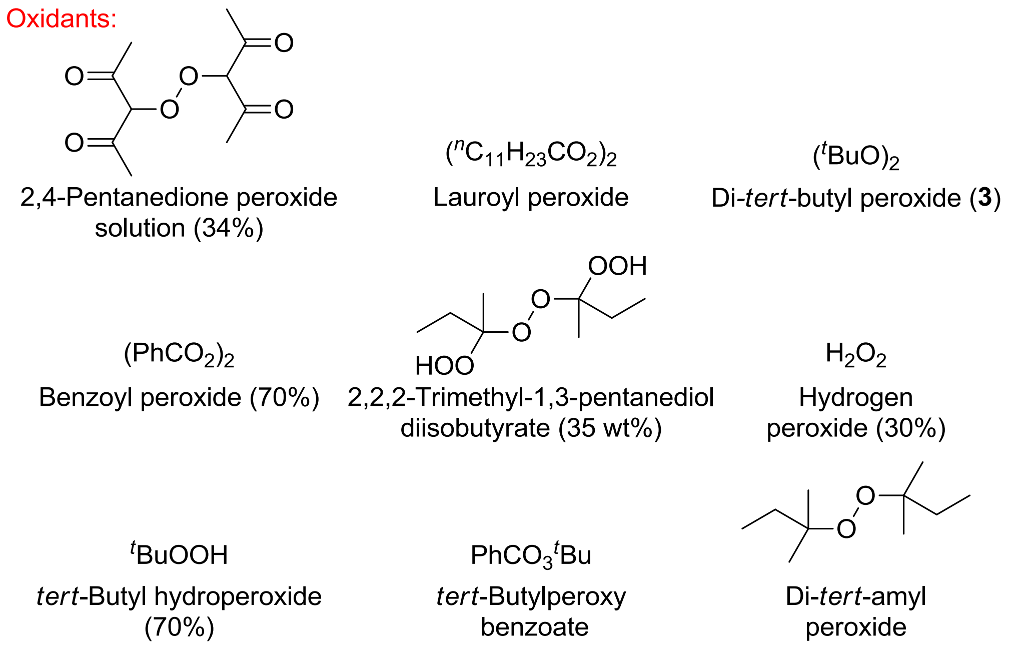

Entry	Oxidant	Yield (%)[a]
1	2,4-Pentanedione peroxide solution (34%)	0
2	Lauroyl peroxide	0
**3**	**Di-^{t}butyl peroxide (3)**	**47**
4	Benzoyl peroxide	0
5	2,2,2-Trimethyl-1,3-pentanediol diisobutyrate (35 wt%)	0
6	Hydrogen peroxide	0
7	^{t}Butylhydroperoxide (70%)	0
8	*tert*-Butyl peroxybenzoate	0

9[b]	Di-*tert*-amyl peroxide	20

[a]Yield was determined by ^{1}H NMR spectrum using 1,1,2,2-tetrachloroethane as an internal standard. [b]The corresponding carbamate (*tert*-amyl cyclohexyl(phenyl)carbamate) was obtained.

Di-*tert*-butyl peroxide (**3**) was the best oxidant for carbamation of cyclohexane.

3-3. Screening of Ligands

In a nitrogen-filled glove box, a flame-dried 10 mL screw cap test tube with a magnetic stir-bar was charged with tetrakis(acetonitrile)copper(I) tetrafluoroborate $[Cu(NCMe)_4]BF_4$ (15.7 mg, 0.050 mmol, 10 mol%) and ligand (0.0500 mmol, 10 mol%). The test tube was removed from the glove box, and cyclohexane (**1a**, 0.54 mL, 5.00 mmol, 10.0 equiv), phenyl isocyanate (**2a**, 59.6 mg, 54.3 μL, 0.500 mmol), di-*tert*-butyl peroxide (**3**, 146 mg, 0.18 mL 1.00 mmol, 2.0 equiv), and benzene (1.0 mL) were added under argon atmosphere. The test tube was placed in preheated oil bath and the mixture was stirred vigorously at 100 °C for 36 h. Cooled to room temperature, the reaction mixture was passed through a short pad of silica gel with the aid of dichloromethane, and concentrated. ^{1}H NMR using 1,1,2,2-tetrachloroethane as an internal standard quantified the yield of **4a**.

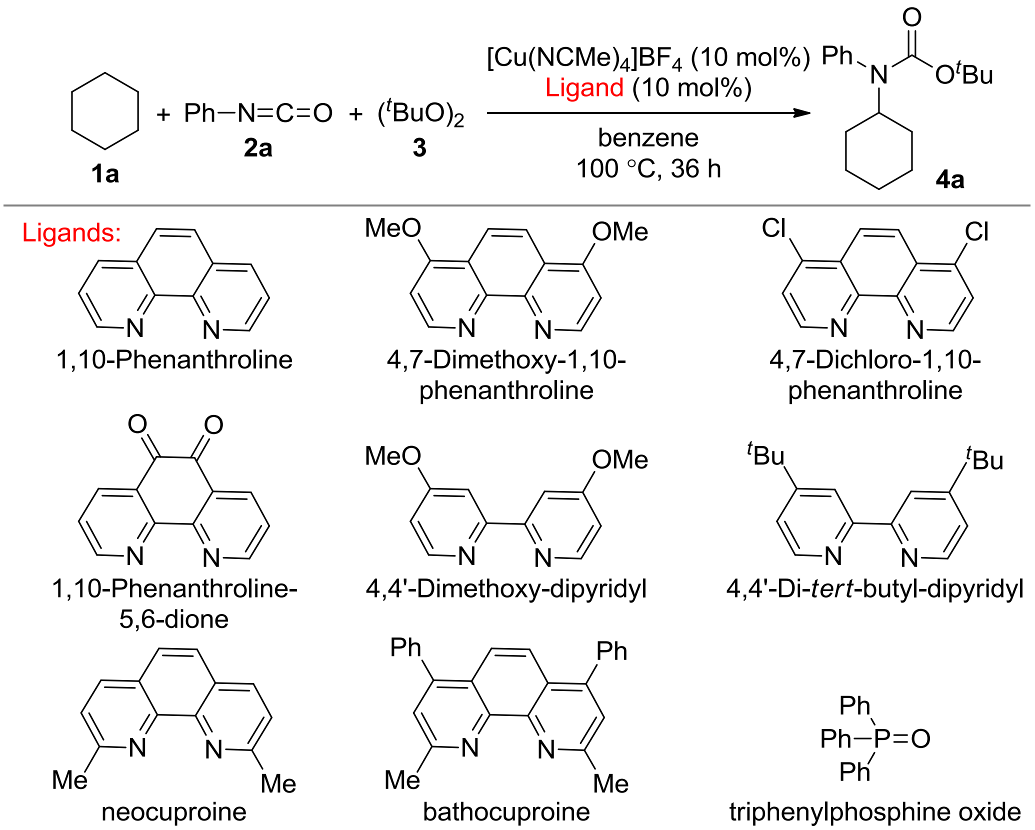

Entry	Ligand	Yield (%)[a]
1	-	32[b]
2	1,10-Phenanthroline	47
3	4,7-Dimethoxy-1,10- phenanthroline	60
4	4,7-Dichloro-1,10-phenanthroline	51
5	1,10-Phenanthroline-5,6-dione	53
6	4,4'-Dimethoxy-dipyridyl	50
7	4,4'-Di-*tert*-butyl-dipyridyl	55
**8**	**neocuproine**	**69**
9	bathocuproine	64

10	triphenylphosphine oxide	38

[a]Yield was determined by ^{1}H NMR spectrum using 1,1,2,2-tetrachloroethane as an internal standard. [b]Reaction was performed without a ligand.

Neocuproine was proved to be the best ligand for carbamation of cyclohexane.

3-4. Screening of Solvents

In a nitrogen-filled glove box, a flame-dried 10 mL screw cap test tube with magnetic stir-bar were charged with tetrakis(acetonitrile)copper(I) tetrafluoroborate $[Cu(NCMe)_4]BF_4$ (15.7 mg, 0.050 mmol, 10 mol%) and neocuproine (10.4 mg, 0.050 mmol, 10 mol%). The test tube was removed from the glove box, and cyclohexane (**1a**, 0.54-1.6 mL, 5.00-15.0 mmol, 10-30 equiv), phenyl isocyanate (**2a**, 59.6 mg, 54.3 μL, 0.500 mmol), di-*tert*-butyl peroxide (146 mg, 0.18 mL, 1.00 mmol, 2.0 equiv), and co-solvent (0.50-1.0 mL) were added under argon atmosphere. The test tube was placed in preheated oil bath and the mixture was stirred vigorously at 100 °C for 36 h. Cooled to room temperature, the reaction mixture was passed through a short pad of silica gel with the aid of dichloromethane, and concentrated. ^{1}H NMR using 1,1,2,2-tetrachloroethane as an internal standard quantified the yield of **4a**.

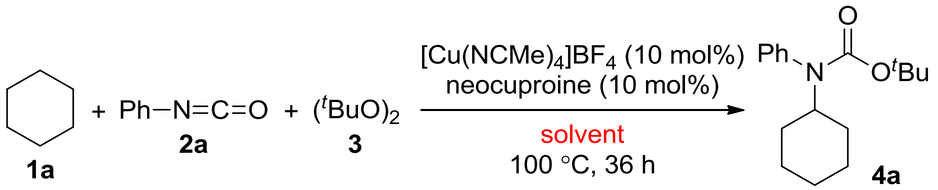

Entry	Alkane (equiv)	Co-solvent	Molarity	Yield (%)^a
1	cyclohexane (10)	-	0.90 M	60
**2**	**cyclohexane (30)**	**-**	**0.30 M**	**78**
3	cyclohexane (10)	benzene	0.50 M	68
**4**	**cyclohexane (10)**	**trifluorotoluene**	**0.50 M**	**69**
5	cyclohexane (10)	1,2-dichloroethane	0.50 M	57
6	cyclohexane (10)	acetonitrile	0.50 M	19
7	cyclohexane (10)	1,2-dichlrobenzene	0.50 M	57
8	cyclohexane (1.0)	benzene	0.50 M	19

^{a}Yield was determined by ^{1}H NMR spectrum using 1,1,2,2-tetrachloroethane as an internal standard.

Trifluorotoluene was proved to be the best solvent for carbamation of cyclohexane.

3-5. Quantities of Di-*tert*-butylperoxide

In an nitrogen-filled glove box, a flame-dried 10 mL screw cap test tube with magnetic stir-bar were charged with tetrakis(acetonitrile)copper(I) tetrafluoroborate $[Cu(NCMe)_4]BF_4$ (15.7 mg, 0.050 mmol, 10 mol%) and neocuproine (10 mg, 0.050 mmol, 10 mol%). The test tube was removed from the glove box, and cyclohexane (**1a**, 0.54 mL, 5.00 mmol, 10.0 equiv), phenyl isocyanate (**2a**, 59.6 mg, 54.3 μL, 0.500 mmol), di-*tert*-butyl peroxide (X equiv), and trifluorotoluene (0.50 mL) were added under argon atmosphere. The test tube was placed in preheated oil bath and the mixture was stirred vigorously at 100 °C for 36 h. Cooled to room temperature, the reaction mixture was passed through a short pad of silica gel with the aid of dichloromethane, and concentrated. ^{1}H NMR using 1,1,2,2-tetrachloroethane as an internal standard quantified the yield of **4a**.

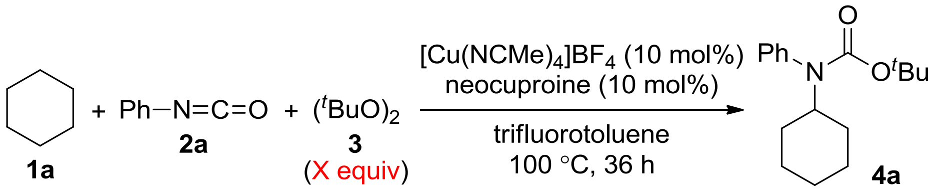

Entry	**3** (**X** equiv)	Yield (%)[a]
1	1.0	35
2	1.5	54
3	2.0	69
**4**	**2.5**	**71**
5	3.0	67
6	4.0	62
7	5.0	58

[a]Yield was determined by ^{1}H NMR spectrum using 1,1,2,2-tetrachloroethane as an internal standard.

2.5 equiv of **3** was proved to be the best amount for carbamation of cyclohexane.

3-6. Screening of Catalyst Loadings

In an nitrogen-filled glove box, a flame-dried 10 mL screw cap test tube with magnetic stir-bar were charged with tetrakis(acetonitrile)copper(I) tetrafluoroborate [Cu(NCMe)]BF_4 (X mol%) and neocuproine (Y mol%). The test tube was removed from the glove box, and cyclohexane (**1a**, 0.54 mL, 5.00 mmol, 10.0 equiv), phenyl isocyanate (**2a**, 59.6 mg, 54.3 μL, 0.500 mmol), di-*tert*-butyl peroxide (183 mg, 0.23 mL, 1.25 mmol, 2.5 equiv), and trifluorotoluene (0.50 mL) were added under argon atmosphere. The test tube was placed in preheated oil bath and the mixture was stirred vigorously at 100 °C for 36 h. Cooled to room temperature, the reaction mixture was passed through a short pad of silica gel with the aid of dichloromethane, and concentrated. ^{1}H NMR using 1,1,2,2-tetrachloroethane as an internal standard quantified the yield of **4a**.

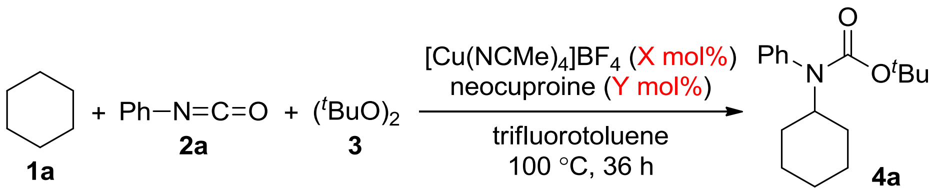

Entry	Cu[(NCMe)$_4$]BF_4 (**X** mol%)	neocuproine (**Y** mol%)	Yield (%)[a]
1	2.0	2.0	63
**2**	**5.0**	**5.0**	**70**
3	10	10	71
4	15	15	72
5	20	20	66
6	100	100	15
7	5.0	7.5	63
8	5.0	10	-
9	10	5.0	49

[a]Yield was determined by ^{1}H NMR spectrum using 1,1,2,2-tetrachloroethane as an internal standard.

5.0 mol% of [Cu(NCMe)$_4$]BF_4 and 5.0 mol% of neocuproine were proved to be the best amounts for carbamation of cyclohexane.

3-7. Screening of Concentrations

In an nitrogen-filled glove box, a flame-dried 10 mL screw cap test tube with magnetic stir-bar were charged with tetrakis(acetonitrile)copper(I) tetrafluoroborate $[Cu(NCMe)_4]BF_4$ (7.9 mg, 0.0250 mmol, 5.0 mol%) and neocuproine (5.0 mg, 0.0250 mmol, 5.0 mol%). The test tube was removed from the glove box, and cyclohexane (**1a**, 0.54 mL, 5.00 mmol, 10.0 equiv), phenyl isocyanate (**2a**, 59.6 mg, 54.3 μL, 0.500 mmol), di-*tert*-butyl peroxide (183 mg, 0.23 mL, 1.25 mmol, 2.5 equiv), and trifluorotoluene were added under argon atmosphere. The test tube was placed in preheated oil bath and the mixture was stirred vigorously at 100 °C for 36 h. Cooled to room temperature, the reaction mixture was passed through a short pad of silica gel with the aid of dichloromethane, and concentrated. ^{1}H NMR using 1,1,2,2-tetrachloroethane as an internal standard quantified the yield of **4a**.

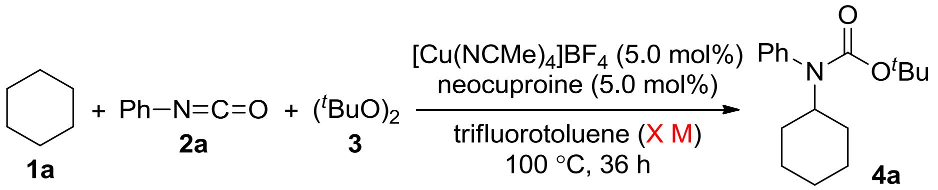

Entry	(**X** M)	Yield (%)[a]
1	0.10 M	47
2	0.30 M	60
**3**	**0.50 M**	**69**
4	0.70 M	67
5[b]	0.90 M	63

[a]Yield was determined by ^{1}H NMR spectrum using 1,1,2,2-tetrachloroethane as an internal standard. [b]Reaction was performed in neat cyclohexane (10 equiv) without adding trifluorotoluene.

0.50 M was proved to be the best concentration for carbamation of cyclohexane.

3-8. Screening of Reaction Temperatures

In an nitrogen-filled glove box, a flame-dried 10 mL screw cap test tube with magnetic stir-bar were charged with tetrakis(acetonitrile)copper(I) tetrafluoroborate $[Cu(NCMe)_4]BF_4$ (7.9 mg, 0.0250 mmol, 5.0 mol%) and neocuproine (5.0 mg, 0.0250 mmol, 5.0 mol%). The test tube was removed from the glove box, and cyclohexane (**1a**, 0.54 mL, 5.00 mmol, 10.0 equiv), phenyl isocyanate (**2a**, 59.6 mg, 54.3 μL, 0.500 mmol), di-*tert*-butyl peroxide (183 mg, 0.23 mL, 1.25 mmol, 2.5 equiv), and trifluorotoluene (0.50 mL) were added under argon atmosphere. The test tube was placed in preheated oil bath and the mixture was stirred vigorously at X °C for 36 h. Cooled to room temperature, the reaction mixture was passed through a short pad of silica gel with the aid of dichloromethane, and concentrated. ^{1}H NMR using 1,1,2,2-tetrachloroethane as an internal standard quantified the yield of **4a**.

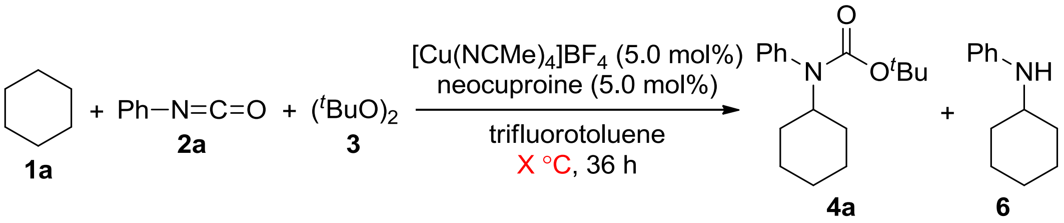

Entry	Temperature (X °C)	Yield (%)[a]	
		**4a**	**6**
1	50	-	-
2	75	16	-
**3**	**100**	**69**	**-**
4	125	42	24
**5**	**150**	**-**	**64**

[a]Yield was determined by ^{1}H NMR spectrum using 1,1,2,2-tetrachloroethane as an internal standard.

(i) Reaction at 100 °C gave tertiary carbamate (**4a**) selectively.
(ii) Reaction at 150 °C underwent thermal cleavage of a Boc group in product **4a** to give *N*-cyclohexylaniline selectively.

3-9. Screening of Reaction Times

In an nitrogen-filled glove box, a flame-dried 10 mL screw cap test tube with magnetic stir-bar were charged with tetrakis(acetonitrile)copper(I) tetrafluoroborate $[Cu(NCMe)_4]BF_4$ (7.9 mg, 0.0250 mmol, 5.0 mol%) and neocuproine (5.0 mg, 0.0250 mmol, 5.0 mol%). The test tube was removed from the glove box, and cyclohexane (**1a**, 0.54 mL, 5.00 mmol, 10.0 equiv), phenyl isocyanate (**2a**, 59.6 mg, 54.3 μL, 0.500 mmol), di-*tert*-butyl peroxide (183 mg, 0.23 mL, 1.25 mmol, 2.5 equiv), and trifluorotoluene (0.50 mL) were added under argon atmosphere. The test tube was placed in preheated oil bath and the mixture was stirred vigorously at 100 °C for X h. Cooled to room temperature, the reaction mixture was passed through a short pad of silica gel with the aid of dichloromethane, and concentrated. ^{1}H NMR using 1,1,2,2-tetrachloroethane as an internal standard quantified the yield of **4a**.

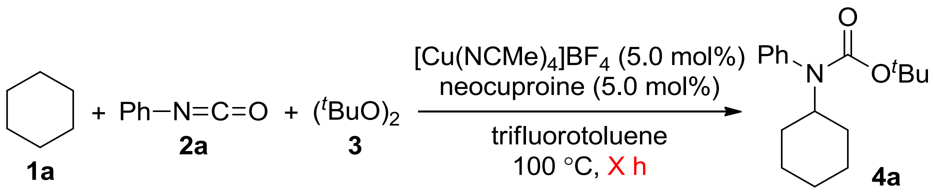

Entry	*Time (X h)*	Yield (%)[a]
1	6 h	54
2	12 h	67
**3**	**24 h**	**75**
4	36 h	69
5	72 h	65

[a]Yield was determined by ^{1}H NMR spectrum using 1,1,2,2-tetrachloroethane as an internal standard.

24 h was proved to be the best reaction time for carbamation of cyclohexane.

4. General Procedure

Procedure A: In a nitrogen-filled glove box, a flame-dried 10 mL screw cap test tube with a magnetic stir-bar was charged with tetrakis (acetonitrile) copper(I) tetrafluoroborate $[Cu(NCMe)_4]BF_4$ (0.0250 mmol, 5.0 mol%) and neocuproine (0.0250 mmol, 5.0 mol%). The test tube was removed from the glove box, an alkane (**1**, 5.0 mmol, 10 equiv), trifluorotoluene (0.50 mL), and di-*tert*-butylperoxide (**3**, 1.25 mmol, 2.5 equiv) were added sequentially under argon atmosphere, and the mixture was stirred at room temperature for 10 minutes. An isocyanate (**2**, 0.500 mmol) was added to the mixture, and the test tube was placed in a preheated oil bath. Then, the mixture was stirred vigorously at 100 °C for 24 h, cooled to room temperature, concentrated under reduced pressure, and purified by column chromatography on silica gel to give the desired tertiary carbamate.

Procedure B: In a nitrogen-filled glove box, a flame-dried 10 mL screw cap test tube with a magnetic stir-bar was charged with tetrakis(acetonitrile)copper(I) tetrafluoroborate $[Cu(NCMe)_4]BF_4$ (0.0250 mmol, 5.0 mol%) and neocuproine (0.0250 mmol, 5.0 mol%). The test tube was removed from the glove box, an alkane (**1**, 15.0 mmol, 30 equiv) and di-*tert*-butylperoxide (**3**, 1.25 mmol, 2.5 equiv) were added under argon atmosphere and the mixture was stirred at room temperature for 10 minutes. An isocyanate (**2**, 0.500 mmol) was added to the reaction mixture and the test tube was placed in a preheated oil bath. Then the mixture was stirred vigorously at 100 °C for 24 h, cooled to room temperature, concentrated under reduced pressure, and purified by column chromatography on silica gel to give the desired tertiary carbamate.

5. Characteristic Data

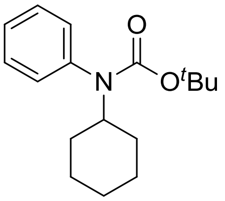

***tert*-Butyl cyclohexyl(phenyl)carbamate (4a).** Purified by silica gel column chromatography (hexane/ethyl acetate = 19:1); R_f = 0.37 (UV active, hexane/ethyl acetate = 9:1); 75% (procedure A), 76% (procedure B); white solid; ^{1}H NMR ($CDCl_3$, 500 MHz) δ 7.26 (dd, J = 7.5, 7.5 Hz, 2H), 7.20 (t, J = 7.5 Hz, 1H), 7.01 (d, J = 7.5 Hz, 2H), 4.09-4.00 (m, 1H), 1.84-1.82 (m, 2H), 1.69-1.66 (m, 2H), 1.54-1.46 (m, 1H), 1.40-1.21 (m, 11H), 1.12-1.04 (m, 2H), 0.91-0.82 (m, 1H); ^{13}C NMR ($CDCl_3$, 125 MHz) δ 154.7, 139.3, 129.8, 128.1, 126.6, 79.2, 56.2, 31.9, 28.1, 25.8, 25.2; IR (thin film) ν 3014, 2933, 1683, 1321, 1215, 756 cm^{-1}; HRMS (ESI) calcd for $C_{17}H_{25}NO_2$ (*m/z*) 298.1783 $[M+Na]^+$ Found 298.1779.

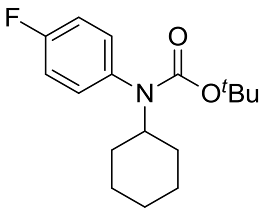

***tert*-Butyl cyclohexyl(4-fluorophenyl)carbamate (4b).** Purified by silica gel column chromatography (hexane/ethyl acetate = 19:1); R_f = 0.47 (UV active, hexane/ethyl acetate = 9:1); 75% (procedure A), 68% (procedure B); white solid; ^{1}H NMR ($CDCl_3$, 500 MHz) δ 7.00 (d, J = 6.9 Hz, 4H), 4.07 (brs, 1H), 1.85 (m, 2H), 1.74 (m, 2H), 1.57 (d, J = 13.2 Hz, 1H), 1.45-1.15 (m, 11H), 1.14-1.03 (m, 2H), 0.95-0.87 (m, 1H); ^{13}C NMR ($CDCl_3$, 125 MHz) δ 161.4 ($^{1}J_{CF}$ = 246 Hz), 154.8, 135.3 ($^{4}J_{CF}$ = 2.4 Hz), 131.5 ($^{3}J_{CF}$ = 8.4 Hz), 115.1 ($^{2}J_{CF}$ = 21.5 Hz), 79.6, 56.1, 32.0, 28.2, 25.8, 25.3; IR (thin film) ν 3018, 2934, 1684, 1508 cm^{-1}; HRMS (ESI) calcd for $C_{17}H_{24}FNO_2$ (*m/z*) 316.1688 $[M+Na]^+$ Found 316.1683.

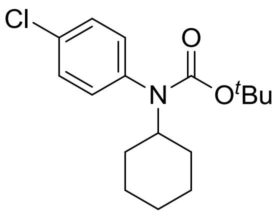

***tert*-Butyl (4-chlorophenyl)(cyclohexyl)carbamate (4c).** Purified by silica gel column chromatography gave white solid (hexane/ethyl acetate = 19:1); R_f = 0.46 (UV active, hexane/ethyl acetate = 9:1); 75% (procedure A), 83% (procedure B); white solid; ^{1}H NMR ($CDCl_3$, 500 MHz) δ 7.28-7.24 (m, 2H), 6.98-6.94 (m, 2H), 4.15-3.95 (m, 1H), 1.85-1.83 (m, 2H), 1.74-1.68 (m, 2H), 1.60-1.50 (m, 1H), 1.40-1.25 (m, 11H), 1.15-1.04 (m, 2H), 0.95-0.86 (m, 1H); ^{13}C NMR ($CDCl_3$, 125 MHz) δ 154.5, 138.0, 132.5, 131.1, 128.4, 79.7, 56.2, 31.9, 28.1, 25.7, 25.2; IR (thin film) ν 3018, 2934, 1684, 1215, 756 cm^{-1}; HRMS (ESI) calcd for $C_{17}H_{24}ClNO_2$ (*m/z*) 332.1393 $[M+Na]^+$ Found 332.1395.

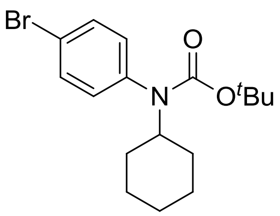

***tert*-Butyl (4-bromophenyl)(cyclohexyl)carbamate (4d).** Purified by silica gel column chromatography (hexane/ethyl acetate = 19:1); R_f = 0.43 (UV active, hexane/ethyl acetate = 9:1); 71% (procedure A), 77% (procedure B); white solid; ^{1}H NMR ($CDCl_3$, 500 MHz) δ 7.43-7.40 (m, 2H), 6.92 (d, J = 8.6 Hz, 2H), 4.09-4.01 (m, 1H), 1.83-1.81 (m, 2H), 1.72-1.69 (m, 2H), 1.55-1.52 (m, 1H), 1.39-1.27 (m, 11H), 1.11-1.03 (m, 2H), 0.94-0.84 (m, 1H); ^{13}C NMR ($CDCl_3$, 125 MHz) δ 154.4, 138.5, 131.6, 131.4, 120.5, 79.7, 56.2, 32.0, 28.2, 25.8, 25.2; IR (thin film) ν 3019, 2935, 1683, 1215, 756 cm^{-1}; HRMS (ESI) calcd for $C_{17}H_{24}BrNO_2$ (*m/z*) 376.0888 $[M+Na]^+$ Found 376.0876.

***tert*-Butyl (2-bromophenyl)(cyclohexyl)carbamate (4e).** Purified by silica gel column chromatography (hexane/ethyl acetate = 49:1); R_f = 0.44 (UV active, hexane/ethyl acetate = 9:1); 62% (procedure A); light yellow solid; ^{1}H NMR ($CDCl_3$, 500 MHz) δ 7.60 (d, *J* = 8.0 Hz, 1H), 7.29-7.27 (m, 1H), 7.16-7.10 (m, 2H), 4.13-3.86 (m, 1H), 2.20-2.05 (m, 1H), 2.00-1.85 (m, 1H), 1.76-1.66 (m, 2H), 1.58-1.20 (m, 13H), 1.05-0.80 (m, 2H); ^{13}C NMR ($CDCl_3$, 125 MHz) δ 153.7, 139.1, 132.9, 130.9, 128.3, 127.4, 126.0, 79.7, 57.1, 32.9, 28.1, 25.9, 25.4; IR (thin film) ν 2933, 1697, 1476, 1320, 1174 cm^{-1}; HRMS (ESI) calcd for $C_{17}H_{24}BrNO_2$ (*m/z*) 376.08881[M+Na]$^+$ Found 376.0903.

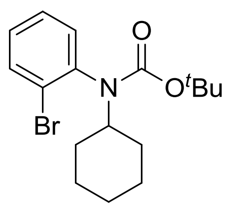

***tert*-Butyl cyclohexyl(4-(trifluoromethyl)phenyl)carbamate (4f).** Purified by silica gel column chromatography (hexane/ethyl acetate = 19:1); R_f = 0.43 (UV active, hexane/ethyl acetate = 9:1); 77% (procedure A), 85% (procedure B); white solid; ^{1}H NMR ($CDCl_3$, 500 MHz) δ 7.56 (d, *J* = 8.0 Hz, 2H), 7.16 (d, *J* = 8.0 Hz, 2H), 4.10-4.03 (m, 1H), 1.86-1.83 (m, 2H), 1.71-1.69 (m, 2H), 1.54-1.50 (m, 1H), 1.45-1.20 (m, 11H), 1.13-1.05 (m, 2H), 0.94-0.85 (m, 1H); ^{13}C NMR ($CDCl_3$, 125 MHz) δ 154.2, 143.0, 130.2, 128.9 (q, $^2J_{CF}$ = 32.0 Hz), 123.9 (q, $^1J_{CF}$ = 272 Hz), 125.4 (q, $^3J_{CF}$ = 3.6 Hz), 80.0, 56.6, 32.0, 28.0, 25.8, 25.1; IR (thin film) ν 3019, 2935, 1686,1324, 1215, 757 cm^{-1}; HRMS (ESI) calcd for $C_{18}H_{24}F_3NO_2$ (*m/z*) 366.1656 [M+Na]$^+$ Found 366.1654.

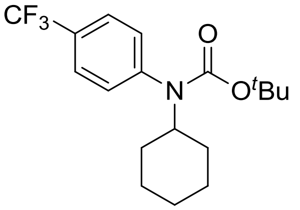

***tert*-Butyl cyclohexyl(2-(trifluoromethyl)phenyl)carbamate (4g).** Purified by silica gel column chromatography (hexane/ethyl acetate = 19:1); R_f = 0.43 (UV active, hexane/ethyl acetate = 9:1); 76% (procedure A), 87% (procedure B); white solid; ^{1}H NMR ($CDCl_3$, 500 MHz) δ 7.52 (d, *J* = 7.5 Hz, 1H), 7.43 (dd, *J* = 7.5, 7.5 Hz, 1H), 7.30 (s, 1H), 7.26 (d, *J* = 7.5 Hz, 1H), 4.11-4.0 (m, 1H), 1.88-1.86 (m, 2H), 1.74-1.72 (m, 2H), 1.57-1.52 (m, 1H), 1.4-1.2 (m, 11H), 1.13-1.05 (m, 2H), 0.93-0.87 (m, 1H); ^{13}C NMR ($CDCl_3$, 125 MHz) δ 154.4, 140.3, 133.3, 130.9 (q, $^2J_{CF}$ = 32.4 Hz), 128.8, 126.9 (q, $^3J_{CF}$ = 3.6 Hz), 123.7 (q, $^1J_{CF}$ = 272 Hz), 123.6 (q, $^3J_{CF}$ = 3.6 Hz), 80.0, 56.6, 32.1, 28.1, 25.8, 25.2; IR (thin film) ν 3019, 2934, 1685, 1317, 1215, 757 cm^{-1}; HRMS (ESI) calcd for $C_{18}H_{24}F_3NO_2$ (*m/z*) 366.1656 [M+Na]$^+$ Found 366.1649.

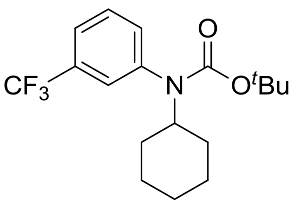

***tert*-Butyl cyclohexyl(4-methoxyphenyl)carbamate (4h).** Purified by silica gel column chromatography (hexane/ethyl acetate = 13.3:1); R_f = 0.4 (UV active, hexane/ethyl acetate = 9:1); 54% (procedure A), 64% (procedure B); white solid; ^{1}H NMR ($CDCl_3$, 500 MHz) δ 6.93 (d, *J* = 6.9 Hz, 2H), 6.82-6.79 (m, 2H), 4.05 (brs, 1H), 3.77 (s, 3H, rotamer), 3.76 (s, 3H, rotamer), 1.83-1.81 (m, 2H), 1.70-1.68 (m, 2H), 1.53-1.20 (m, 12H), 1.11-1.03 (m, 2H), 0.93-0.86 (m, 1H); ^{13}C NMR ($CDCl_3$, 125 MHz) δ 158.1, 155.1, 132.0, 130.7, 113.3, 79.1, 55.1 (2C), 31.9, 28.2, 25.8, 25.2; IR (thin film) ν 3007, 2932, 2857, 1682, 1512,

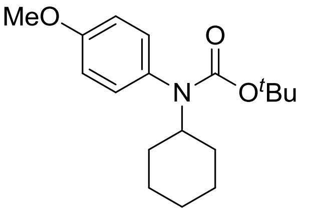

1247, 757 cm^{-1}; HRMS (ESI) calcd for $C_{18}H_{27}NO_3$ (*m/z*) 328.1888 [M+Na]$^+$ Found 328.1905.

***tert*-Butyl cyclohexyl(*p*-tolyl)carbamate (4i).** Purified by silica gel column chromatography (hexane/ethyl acetate = 19:1); R_f = 0.44 (UV active, hexane/ethyl acetate = 9:1); 63% (procedure A), 70% (procedure B); white solid; ^{1}H NMR ($CDCl_3$, 500 MHz) δ 7.10 (d, *J* = 8.6 Hz, 2H), 6.92 (d, *J* = 8.0 Hz, 2H), 4.15-3.95 (m, 1H), 2.33 (s, 3H), 1.86-1.83 (m, 2H), 1.72-1.70 (m, 2H), 1.55-1.50 (m, 1H), 1.38-1.28 (m, 11H), 1.15-1.07 (m, 2H), 0.95-0.89 (m, 1H); ^{13}C NMR ($CDCl_3$, 125 MHz) δ 155.0, 136.7, 136.3, 129.6, 128.9, 79.2, 56.2, 32.0, 28.2, 25.8, 25.3, 20.9; IR (thin film) ν 2934, 1683, 1215, 756 cm^{-1}; HRMS (ESI) calcd for $C_{19}H_{27}NO_2$ (*m/z*) 312.1939 [M+Na]$^+$ Found 312.1925.

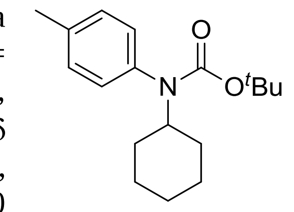

***tert*-Butyl (4-butylphenyl)(cyclohexyl)carbamate (4j).** Purified by silica gel column chromatography (hexane/ethyl acetate = 19:1); R_f = 0.46 (UV active, hexane/ethyl acetate = 9:1); 61% (procedure A), 70% (procedure B); colorless liquid; ^{1}H NMR ($CDCl_3$, 500 MHz) δ 7.10 (d, *J* = 8.3 Hz, 2H), 6.93 (d, *J* = 8.3 Hz, 2H), 4.15-3.90 (m, 1H), 2.59 (t, *J* = 8.0 Hz, 2H), 1.86-1.84 (m, 2H), 1.76-1.70 (m, 2H), 1.62-1.53 (m, 3H), 1.45-1.25 (m, 13H), 1.17-1.09 (m, 2H), 0.95-0.88 (m, 4H); ^{13}C NMR ($CDCl_3$, 125 MHz) δ 155.0, 141.3, 136.9, 129.5, 128.2, 79.2, 56.3, 35.1, 33.4, 32.0, 28.2, 25.9, 25.3, 22.2, 13.8; IR (thin film) ν 2931, 2857, 1683, 1321, 1153, 756 cm^{-1}; HRMS (ESI) calcd for $C_{21}H_{33}NO_2$ (*m/z*) 354.2409 [M+Na]$^+$ Found 354.2414.

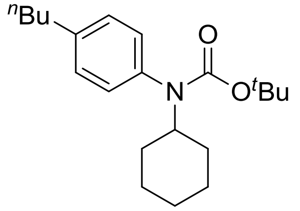

***tert*-Butyl (4-acetylphenyl)(cyclohexyl)carbamate (4k).** Purified by silica gel column chromatography (hexane/ethyl acetate = 9:1); R_f = 0.18 (UV active, hexane/ethyl acetate = 9:1); 68% (procedure A), 47% (procedure B); yellow liquid; ^{1}H NMR ($CDCl_3$, 500 MHz) δ 7.88 (d, *J* = 8.6 Hz, 2H), 7.12 (d, *J* = 8.6 Hz, 2H), 4.07-4.01 (m, 1H), 2.56 (s, 3H), 1.85-1.82 (m, 2H), 1.71-1.68 (m, 2H), 1.51-1.49 (m, 1H), 1.35-1.25 (m, 11H), 1.15-1.07 (m, 2H), 0.93-0.84 (m, 1H); ^{13}C NMR ($CDCl_3$, 125 MHz) δ 197.2, 154.2, 144.3, 135.4, 129.9, 128.4, 79.9, 56.7, 32.0, 28.1, 26.4, 25.8, 25.2; IR (thin film) ν 3007, 2932, 2857, 1685, 1602, 1174, 754 cm^{-1}; HRMS (ESI) calcd for $C_{19}H_{27}NO_3$ (*m/z*) 340.1888 [M+Na]$^+$ Found 340.1883.

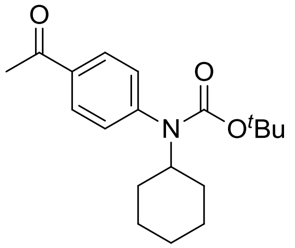

Methyl 3-((*tert*-butoxycarbonyl)(cyclohexyl)amino)benzoate (4l). Purified by silica gel column chromatography (hexane/ethyl acetate = 13.3:1); R_f = 0.3 (UV active, hexane/ethyl acetate = 9:1); 73% (procedure A), 70% (procedure B); white solid; ^{1}H NMR ($CDCl_3$, 500 MHz) δ 7.89 (d, *J* = 8.0 Hz, 1H), 7.68 (s, 1H), 7.34 (t, *J* = 8.0 Hz, 1H), 7.20 (d, *J* = 7.5 Hz, 1H), 4.06-3.99 (m, 1H), 3.85 (s, 3H), 1.84-1.81 (m, 2H), 1.68-1.65 (m, 2H), 1.50-1.41 (m, 1H), 1.31-1.15 (m, 11H), 1.08-1.00 (m, 2H), 0.89-0.80 (m, 1H); ^{13}C NMR

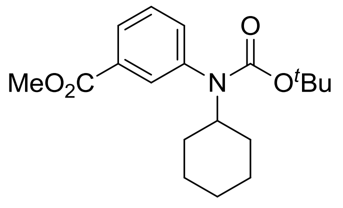

($CDCl_3$, 125 MHz) δ 166.3, 154.4, 139.6, 134.5, 130.8, 130.4, 128.2, 127.9, 79.6, 56.2, 51.9, 31.9, 28.0, 25.7, 25.1; IR (thin film) ν 3019, 2934, 1718, 1685, 1215 cm^{-1}; HRMS (ESI) calcd for $C_{19}H_{27}NO_4$ (*m/z*) 356.1837 $[M+Na]^+$ Found 356.1849.

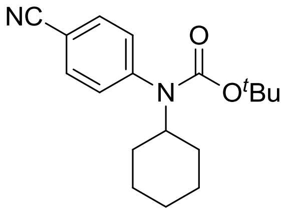

***tert*-Butyl (4-cyanophenyl)(cyclohexyl)carbamate (4m).** Purified by silica gel column chromatography (hexane/ethyl acetate = 9:1); R_f = 0.28 (UV active, hexane/ethyl acetate = 9:1); 70% (procedure A); light yellow solid; ^{1}H NMR ($CDCl_3$, 500 MHz) δ 7.61 (d, *J* = 8.3 Hz, 2H), 7.17 (d, *J* = 8.3 Hz, 2H), 4.10-4.00 (m, 1H), 1.85-1.83 (m, 2H), 1.74-1.71 (m, 2H), 1.57-1.53 (m, 1H), 1.42-1.24 (m, 11H), 1.15-1.07 (m, 2H), 0.97-0.87 (m, 1H); ^{13}C NMR ($CDCl_3$, 125 MHz) δ 153.9, 144.2, 132.3, 130.6, 118.4, 110.6, 80.4, 57.0, 32.0, 28.1, 25.8, 25.2; IR (thin film) ν 2934, 2225, 1686, 1367, 1318, 769 cm^{-1}; HRMS (ESI) calcd for $C_{18}H_{24}N_2O_2$ (*m/z*) 323.1735 $[M+Na]^+$ Found 323.1730.

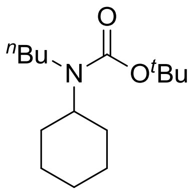

***tert*-Butyl butyl(cyclohexyl)carbamate (4n).** Purified by silica gel column chromatography (hexane/ethyl acetate = 32.3:1); R_f = 0.46 (iodine stain, hexane/ethyl acetate = 9:1); 33% (procedure A), 42% (procedure B); yellow liquid; ^{1}H NMR ($CDCl_3$, 500 MHz) δ 3.82-3.51 (m, 1H), 3.15-2.85 (m, 2H), 1.74-1.57 (m, 5H), 1.46-1.22 (m, 17H), 1.08-0.99 (m, 1H), 0.87 (t, *J* = 7.5 Hz, 3H); ^{13}C NMR ($CDCl_3$, 125 MHz) δ 155.4, 78.7, 54.8, 42.8, 32.8, 31.2, 28.4, 26.0, 25.5, 20.2, 13.8; IR (thin film) ν 3008, 2932, 2857, 1679, 1412, 1156, 755 cm^{-1}; HRMS (ESI) calcd for $C_{15}H_{29}NO_2$ (*m/z*) 278.2096 $[M+Na]^+$ Found 278.2086.

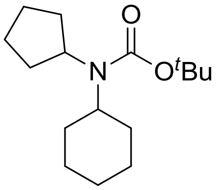

***tert*-Butyl cyclohexyl(cyclopentyl)carbamate (4o).** Purified by silica gel column chromatography (hexane/ethyl acetate = 19:1); R_f = 0.44 (Iodine stain, hexane/ethyl acetate = 9:1); 34% (procedure A), 34% (procedure B); yellow liquid; ^{1}H NMR ($CDCl_3$, 500 MHz) δ 4.28-3.10 (m, 2H), 1.85-1.55 (m, 13H), 1.50-1.38 (m, 11H), 1.31-1.24 (m, 2H), 1.08-0.99 (m, 1H); ^{13}C NMR ($CDCl_3$, 125 MHz) δ 155.1, 79.0, 55.2, 54.9, 31.3, 29.9, 28.6, 26.1, 25.5, 24.6; IR (thin film) ν 3008, 2931, 2856, 1670, 1453, 1159, 755 cm^{-1}; HRMS (ESI) calcd for $C_{15}H_{29}NO_2$ (*m/z*) 290.2096 $[M+Na]^+$ Found 290.2098.

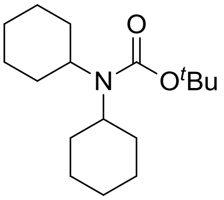

***tert*-Butyl dicyclohexylcarbamate (4p).** Purified by silica gel column chromatography (hexane/ethyl acetate = 19:1); R_f = 0.42 (iodine stain, hexane/ethyl acetate = 9:1); 30% (procedure A), 29% (procedure B); yellow liquid; ^{1}H NMR ($CDCl_3$, 500 MHz) δ 3.97-3.61 (m, 1H), 3.20-2.80 (m, 1H) 1.71-1.42 (m, 22H), 1.29-1.16 (m, 5H), 1.05-0.97 (m, 2H); ^{13}C NMR ($CDCl_3$, 125 MHz) δ 155.3, 78.8, 54.7, 31.2, 28.5, 26.2, 25.5; IR (thin film) ν 3009, 2932, 2855, 1670, 1439, 1159, 757 cm^{-1}; HRMS (ESI) calcd for $C_{15}H_{29}NO_2$ (*m/z*) 304.2252 $[M+Na]^+$ Found 304.2268.

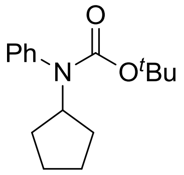

***tert*-Butyl cyclopentyl(phenyl)carbamate (4q).** Purified by silica gel column chromatography (hexane/ethyl acetate = 19:1); R_f = 0.34 (UV active, hexane/ethyl acetate = 9:1); 51% (procedure A, cyclopentane (30 equiv)); yellow solid; ^{1}H NMR ($CDCl_3$, 500 MHz) δ 7.34-7.30 (m, 2H), 7.25-7.23 (m, 1H), 7.08-7.06 (m, 2H), 4.50-4.43 (m, 1H), 1.90-1.84 (m, 2H), 1.52-1.47 (m, 3H), 1.43-1.33 (m, 12H); ^{13}C NMR ($CDCl_3$, 125 MHz) δ; 155.2, 140.0, 129.7, 128.4, 126.7, 79.6, 58.9, 30.0, 28.3, 22.9; IR (thin film) ν 3019, 2360, 1683, 1215, 768 cm^{-1}; HRMS (ESI) calcd for $C_{16}H_{23}NO_2$ (*m/z*) 284.1626 $[M+Na]^+$ Found 284.1640.

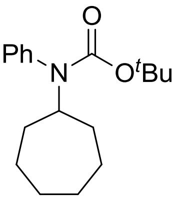

***tert*-Butyl cycloheptyl(phenyl)carbamate (4r).** Purified by silica gel column chromatography (hexane/ethyl acetate = 19:1); R_f = 0.4 (UV active, hexane/ethyl acetate = 9:1); 66% (procedure A); white solid; ^{1}H NMR ($CDCl_3$, 500 MHz) δ 7.31 (dd, *J* = 7.5, 7.5 Hz, 2H), 7.23 (t, *J* = 7.5 Hz, 1H), 7.10 (d, *J* = 7.5 Hz, 2H), 4.30-3.90 (m, 1H), 1.97-1.93 (m, 2H), 1.69-1.37 (m, 19H); ^{13}C NMR ($CDCl_3$, 125 MHz) δ 154.7, 140.7, 129.3, 128.3, 126.5, 79.5, 59.6, 34.4, 28.3, 27.2, 25.0; IR (thin film) ν 2930, 1684, 1167, 757 cm^{-1}; HRMS (ESI) calcd for $C_{18}H_{27}NO_2$ (*m/z*) 312.1939 $[M+Na]^+$ Found 312.1949.

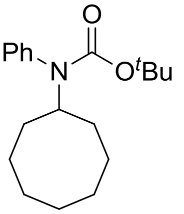

***tert*-Butyl cyclooctyl(phenyl)carbamate (4s).** Purified by silica gel column chromatography (hexane/ethyl acetate = 19:1); R_f = 0.4 (UV active, hexane/ethyl acetate = 9:1); 63% (procedure A); white solid; ^{1}H NMR ($CDCl_3$, 500 MHz) δ 7.31 (dd, *J* = 7.5, 7.5 Hz, 2H), 7.23 (t, *J* = 7.5 Hz, 1H), 7.10 (d, *J* = 7.5 Hz, 2H), 4.30-4.10 (m, 1H), 1.90-1.86 (m, 2H), 1.69-1.63 (m, 4H), 1.56-1.38 (m, 17H); ^{13}C NMR ($CDCl_3$, 125 MHz) δ 154.7, 141.1, 129.1, 128.3, 126.4, 58.5, 33.3, 28.3, 26.4, 26.0, 25.0; IR (thin film) ν 3019, 1682, 1215, 756 cm^{-1}; HRMS (ESI) calcd for $C_{19}H_{29}NO_2$ (*m/z*) 326.2096 $[M+Na]^+$ Found 326.2108.

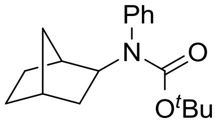

***tert*-Butyl (bicyclo[2.2.1]heptan-2-yl)(phenyl)carbamate (4t).** Purified by silica gel column chromatography (hexane/ethyl acetate = 32.3:1); R_f = 0.4 (UV active, hexane/ethyl acetate = 9:1); 46% (*exo* : *endo* = 87:13) (procedure A, trifluorotoluene (1.5 mL) as a co-solvent); white solid; ^{1}H NMR ($CDCl_3$, 500 MHz) (mixture of *exo*- and *endo*-products, *exo*:*endo* = 6.7:1.0) δ 7.34-7.29 (m, 2H), 7.27-7.24 (m, 1H), 7.09-7.04 (m, 2H), 4.19-4.17 (m, 0.13H), 4.16-4.10 (m, 0.87 H), 2.25-2.24 (m, 1H), 2.10-2.05 (m, 1H), 1.76-1.72 (m, 1H), 1.50-1.25 (m, 13H), 1.13-1.09 (m, 1H), 0.89-0.81 (m, 2H); ^{13}C NMR ($CDCl_3$, 125 MHz) (mixture of *exo*- and *endo*-products, *exo*:*endo* = 6.7:1.0) δ 155.6, 142.5 (140.1), 130.6 (129.4), 128.4 (128.5), 126.6 (126.3), 79.5 (79.6), 59.9 (60.4), 41.8 (41.3), 38.7 (37.9), 35.7 (36.5), 35.7 (34.6), 28.5 (29.7), 28.3 (27.0), 28.2 (21.7); IR (thin film) ν 2960, 1684, 1367, 1166, 755 cm^{-1}; HRMS (ESI) calcd for $C_{18}H_{25}NO_2$ (*m/z*) $[M+Na]^+$ Found 310.1779.

tert-Butyl ((3s,5s,7s)-adamantan-1-yl)(phenyl)carbamate (4ua). Purified by silica gel column chromatography (hexane/ethyl acetate = 32.3:1); R_f = 0.37 (UV active, hexane/ethyl acetate = 9:1); 26% (procedure A, trifluorotoluene (2 mL) as a co-solvent); white solid; ^{1}H NMR ($CDCl_3$, 500 MHz) δ; 7.30-7.26 (m, 2H), 7.24-7.21 (m, 1H), 7.04-7.02 (m, 2H), 2.03 (s, 9H), 1.65-1.61 (m, 6H), 1.31 (s, 9H); ^{13}C NMR ($CDCl_3$, 125 MHz) δ 154.6, 141.4, 130.2 127.9, 126.5, 79.1, 57.2, 41.4, 36.3, 30.1, 28.3; IR (thin film) ν 3019, 1685, 1215, 761 cm^{-1}; HRMS (ESI) calcd for $C_{21}H_{29}NO_2$ (*m/z*) 350.2096 [M+Na]$^+$ Found 350.2083.

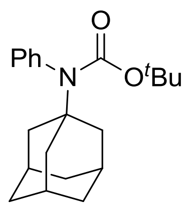

tert-Butyl ((1r,3r,5r,7r)-adamantan-2-yl)(phenyl)carbamate (4ub). Purified by silica gel column chromatography (hexane/ethyl acetate = 32.3:1); R_f = 0.46 (UV active, hexane/ethyl acetate = 9:1); 16% (procedure A, trifluorotoluene (2 mL) as a co-solvent); white solid; ^{1}H NMR ($CDCl_3$, 500 MHz) δ; 7.31 (dd, *J* = 7.5, 7.5 Hz, 2H), 7.26-7.23 (m, 1H), 7.19-7.16 (m, 2H), 4.25-4.23 (brs, 1H), 2.30-2.20 (m, 2H), 1.90-1.78 (m, 5H), 1.67-1.60 (m, 3H), 1.48-1.46 (m, 2H), 1.33-1.26 (m, 11H); ^{13}C NMR ($CDCl_3$, 125 MHz) (rotamers) δ 155.7, 140.9, 130.7, 128.1, 126.7, 79.4, 61.6, 38.6 (37.8), 31.9, 31.1, 28.2 (27.5), 26.9; IR (thin film) ν 2911, 1682, 1338, 1165, 755 cm^{-1}; HRMS (ESI) calcd for $C_{21}H_{29}NO_2$ (*m/z*) 350.2096 [M+Na]$^+$ Found 350.2101.

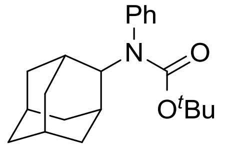

tert-Butyl benzyl(phenyl)carbamate (4v). Purified by silica gel column chromatography (hexane/ethyl acetate = 19:1); R_f = 0.37 (UV active, hexane/ethyl acetate = 9:1); 44% (procedure A); colorless liquid; ^{1}H NMR ($CDCl_3$, 500 MHz) δ 7.32-7.23 (m, 7H), 7.17-7.15 (m, 3H), 4.85 (s, 2H), 1.44 (s, 9H); ^{13}C NMR ($CDCl_3$, 125 MHz) δ 154.8, 142.8, 138.6, 128.5, 128.3, 127.3, 127.0, 126.5, 125.7, 80.4, 53.9, 28.2; IR (thin film) ν 3017, 1688, 1391, 1215, 1165, 756 cm^{-1}; HRMS (ESI) calcd for $C_{18}H_{21}NO_2$ (*m/z*) 306.1470 [M+Na]$^+$ Found 306.1462.

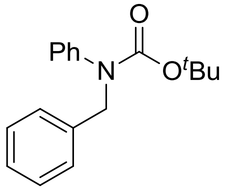

tert-Butyl phenyl(1-phenylethyl)carbamate (4w). Purified by silica gel column chromatography (hexane/ethyl acetate = 32.3:1); R_f = 0.41 (UV active) (hexane/ethyl acetate = 9:1); 65% (procedure A); colorless liquid; ^{1}H NMR ($CDCl_3$, 500 MHz) δ 7.37-7.30 (m, 5H), 7.29-7.24 (m, 3H), 6.89 (tt, *J* = 6.3, 6.3 Hz, 2H), 5.85-5.70 (m, 1H), 1.54 (d, *J* = 6.9 Hz, 3H), 1.43 (s, 9H); ^{13}C NMR ($CDCl_3$, 125 MHz) δ 155.0, 142.2, 139.3, 129.6, 128.1, 128.0, 127.3, 127.0, 126.6, 79.9, 54.9, 28.2, 18.0; IR (thin film) ν 3063, 2977, 1692, 1496, 1322, 1168, 757 cm^{-1}; HRMS (ESI) calcd for $C_{19}H_{23}NO_2$ (*m/z*) 320.1626 [M+Na]$^+$ Found 320.1635.

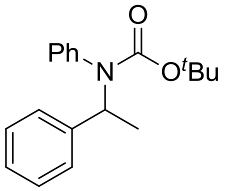

Amidation of *n*-hexane.

The reaction of *n*-hexane (**1x**) with phenyl isocyanate (**2a**) and di-*tert*-butyl peroxide (**3**) was performed by procedure A. The crude reaction mixture was purified by column chromatography on silica gel to give a colorless liquid as a mixture of regioisomers in the ratio (3.2:1.4:1) (hexane/ethyl acetate = 32.3:1). The regioisomers were separated by recycling preparative HPLC [Japan Analytical Industry Co., Ltd. LC9210NEXT equipped with JAIGEL-1H and JAIGEL-2H columns, and $CHCl_3$ was used as an eluent]

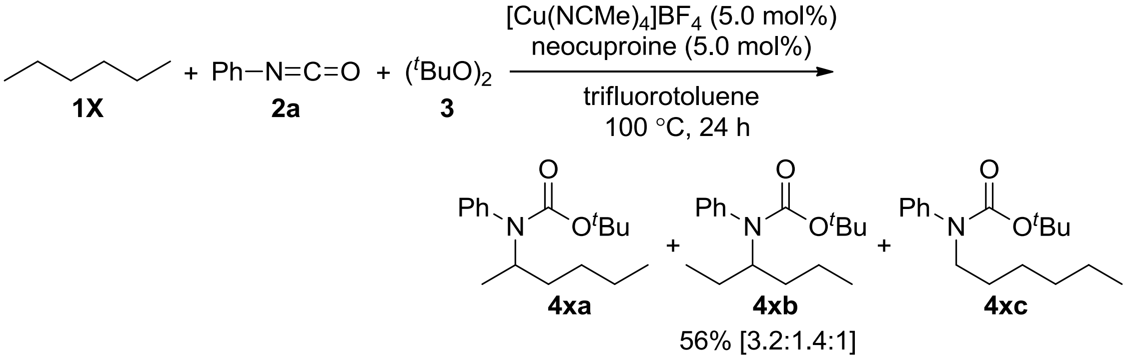

***tert*-Butyl hexan-2-yl(phenyl)carbamate (4xa).** R_f = 0.41 (UV active, hexane/ethyl acetate = 9:1); 32%; colorless liquid; ^{1}H NMR ($CDCl_3$, 500 MHz) δ 7.32 (dd, *J* = 7.5, 7.5 Hz, 2H), 7.26-7.23 (m, 1H), 7.08 (d, *J* = 8.0 Hz, 2H), 4.40-4.22 (m, 1H), 1.57-1.52 (m, 1H), 1.37-1.25 (m, 14H), 1.09 (d, *J* = 6.9 Hz, 3H), 0.89 (t, *J* = 7.5 Hz, 3H); ^{13}C NMR ($CDCl_3$, 125 MHz) δ 155.1, 139.6, 129.7, 128.4, 126.7, 79.5, 53.1, 35.2, 29.0, 28.3, 22.6, 19.7, 14.0; IR (thin film) ν 2973, 2872, 1697, 1336, 1176, 756 cm^{-1}; HRMS (ESI) calcd for $C_{17}H_{27}NO_2$ (*m/z*) 300.1939 [M+Na]$^+$ Found 300.1927.

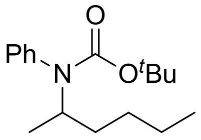

***tert*-Butyl hexan-2-yl(phenyl)carbamate (4xb).** R_f = 0.41 (hexane/ethyl acetate = 9:1); 14%; colorless liquid; ^{1}H NMR ($CDCl_3$, 500 MHz) δ 7.32 (dd, *J* = 7.5, 7.5 Hz, 2H), 7.26-7.23 (m, 1H), 7.09 (d, *J* = 8.0 Hz, 2H), 4.35-4.00 (m, 1H), 1.50-1.26 (m, 15H), 1.00 (t, *J* = 7.5 Hz, 3H), 0.92 (t, *J* = 7.5 Hz, 3H); ^{13}C NMR ($CDCl_3$, 125 MHz) (Mixture of rotamers) δ 155.6, 139.9 (143.7), 129.4 (129.7), 128.4 (127.4), 126.6 (126.7), 79.5 (80.3), 59.5, 35.7 (35.2), 28.9 (29.0), 28.3 (26.7), 20.0 (22.6), 14.06 (14.08), 11.5; IR (thin film) ν 2965, 1685, 1366, 1166, 755 cm^{-1}; HRMS (ESI) calcd for $C_{17}H_{27}NO_2$ (*m/z*) 300.1939 [M+Na]$^+$ Found 300.1945.

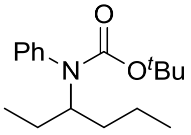

***tert*-Butyl hexyl(phenyl)carbamate (4xc).** R_f = 0.41 (UV active, hexane/ethyl acetate = 9:1); 10%; colorless liquid; ^{1}H NMR ($CDCl_3$, 500 MHz) δ 7.32 (dd, *J* = 7.5, 7.5 Hz, 2H), 7.20-7.17 (m, 3H), 3.60 (t, *J* = 7.5 Hz, 2H), 1.55-1.38 (m, 11H), 1.32-1.20 (m, 6H), 0.86 (t, *J* = 6.9 Hz, 3H); ^{13}C NMR ($CDCl_3$, 125 MHz) δ 154.7, 142.6, 128.6, 127.1, 125.8, 79.8, 50.0, 31.4, 28.4, 28.3, 26.3, 22.5, 13.9; IR (thin film) ν 2930, 2361, 1697, 1392, 1151 cm^{-1}; HRMS (ESI) calcd for $C_{17}H_{27}NO_2$ (*m/z*) 300.1939 [M+Na]$^+$ Found 300.1945.

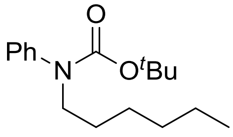

6. Evaluation of Reactivity Difference between Carbamate and Isocyanate

Procedure C. In a nitrogen-filled glove box, a flame-dried 10 mL screw cap test tube with a magnetic stir-bar was charged with tetrakis(acetonitrile)copper(I) tetrafluoroborate $[Cu(NCMe)_4]BF_4$ (7.9 mg, 0.0250 mmol, 5.0 mol%) and neocuproine (5.0 mg, 0.0250 mmol, 5.0 mol%). The test tube was removed from the glove box. Cyclohexane (**1a**, 420 mg, 0.54 mL, 5.00 mmol, 10 equiv), trifluorotoluene (0.50 mL), di-*tert*-butylperoxide (**3**, 183 mg, 0.23 mL, 1.25 mmol, 2.5 equiv), and *n*-butyl isocyanate (**2n**, 49.6 mg, 56.3 μL, 0.500 mmol) or *t*-butyl butylcarbamate (**5**, 86.6 mg, 0.500 mmol) were added sequentially under argon atmosphere. The test tube was placed in a preheated oil bath and the mixture was stirred vigorously at 100 °C for 24 h. Cooled to room temperature, the reaction mixture was concentrated under reduced pressure, and purified by column chromatography on silica gel (hexane/ethyl acetate = 32.3:1) to give the desired tertiary carbamate **4n** as a yellow liquid.

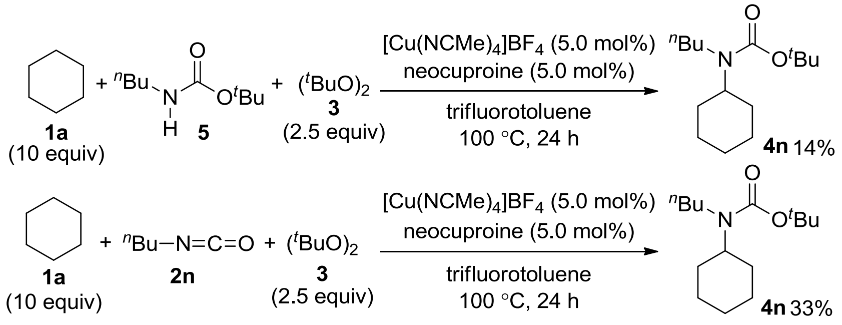

Procedure D. In a nitrogen-filled glove box, a flame-dried 10 mL screw cap test tube with a magnetic stir-bar was charged with tetrakis(acetonitrile)copper(I) tetrafluroborate $[Cu(NCMe)_4]BF_4$ (7.9 mg, 0.0250 mmol, 5.0 mol%) and neocuproine (5.0 mg, 0.0250 mmol, 5.0 mol%). The test tube was removed from the glove box. Cyclohexane (**1a**, 1.26 g, 1.6 mL, 15.0 mmol, 30 equiv), di-*tert*-butyl peroxide (**3**, 183 mg, 0.23 mL, 1.25 mmol, 2.5 eq.), and *n*-butyl isocyanate (**2n**, 49.6 mg, 56.3 μL, 0.500 mmol) or *t*-butyl butylcarbamate (86.6 mg, 0.500 mmol) were added sequentially under argon atmosphere. The test tube was placed in a preheated oil bath and the mixture was stirred vigorously at 100 °C for 24 h. Cooled to room temperature, the reaction mixture was concentrated under reduced pressure, and purified by column chromatography on silica gel (hexane/ethyl acetate = 32.3:1) to give the desired tertiary carbamate **2n** as a yellow liquid.

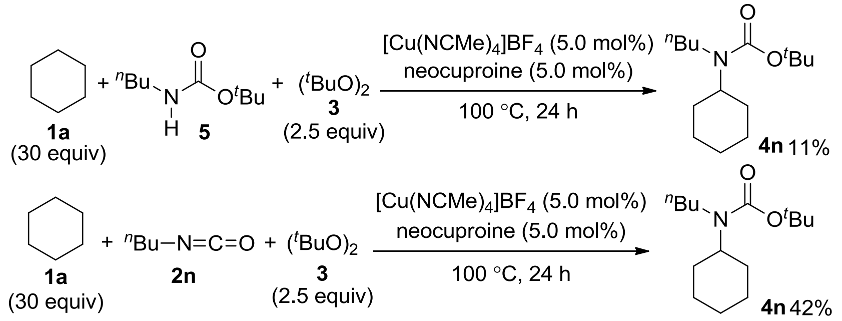

7. Several Experiments for understanding the reaction mechanism:

(1) Reaction of Cyclohexane (1a), Isocyanate (2a), and *tert*-Butyl peroxide (3)

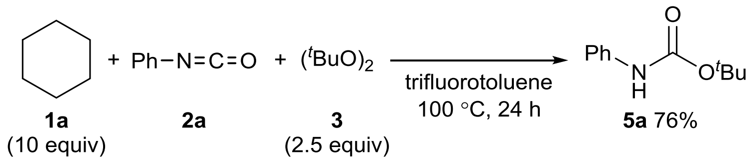

Experimental procedure: In a flame-dried 10 mL screw cap test tube with a magnetic stir-bar was charged with cyclohexane (**1a**, 0.42 g, 0.54 mL, 5.0 mmol, 10 equiv), di-*tert*-butyl peroxide (**3**, 183 mg, 0.23 mL, 1.25 mmol, 2.5 equiv), phenyl isocyanate (**2a**, 59.6 mg, 54.3 μL, 0.500 mmol), and triflurotoluene (1.0 mL) under argon atmosphere. The test tube was placed in a preheated oil bath and the mixture was stirred vigorously at 100 °C for 24 h. The reaction mixture was cooled to room temperature, concentrated under reduced pressure, and purified by column chromatography on silica gel (hexane/ethyl acetate = 9/1) to give the desired *tert*-butyl phenylcarbamate (**5a**) as a white solid (73.5 mg, 76%). All the spectral data were in accordance with the reported data.[3] ^{1}H NMR ($CDCl_3$, 500 MHz) δ 7.33 (d, *J* = 8.0 Hz, 2H), 7.26-7.22 (m, 2H), 6.99 (t, *J* = 7.5 Hz, 1H), 6.45 (brs, 1H), 1.49 (s, 9H); ^{13}C NMR ($CDCl_3$, 125 MHz) δ 152.7, 138.3, 128.9, 122.9, 118.5, 80.4, 28.3.

(2) Reaction between Phenyl isocyanate (2a) and peroxide (3) without addition of cyclohexane (1a)

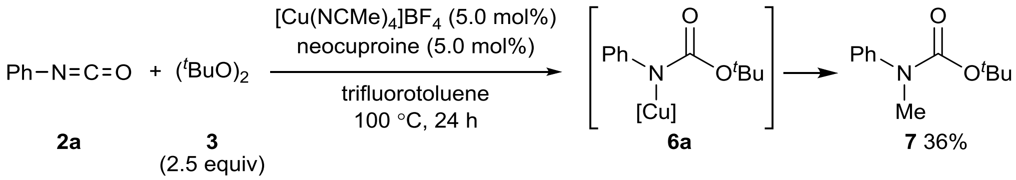

In a nitrogen-filled glove box, a flame-dried 10 mL screw cap test tube with a magnetic stir-bar was charged with tetrakis(acetonitrile)copper(I) tetrafluoroborate $[Cu(NCMe)_4]BF_4$ (15.7 mg, 0.0500 mmol, 10 mol%) and neocuproine (10.4 mg, 0.0500 mmol, 10 mol%). The test tube was removed from the glove box, and trifluorotoluene (1.5 mL), di-*tert*-butylperoxide (**3**, 183 mg, 0.23 mL, 1.25 mmol, 2.5 equiv), and phenyl isocyanate (**2a**, 59.6 mg, 54.3 μL, 0.500 mmol) were added sequentially under argon atmosphere. The test tube was placed in a preheated oil bath and the mixture was stirred vigorously at 100 °C for 24 h. The reaction mixture was cooled to room temperature, concentrated under reduced pressure, and purified by column chromatography on silica gel (hexane/ethyl acetate = 9:1) to give tertiary carbamate **7** as a colorless liquid (23.0 mg, 36%). All the obtained spectral data were in accordance with the literature reported data.[4]

---

[3] U. Streit, F. Birbaum, A. Quattropani, C. G. Bochet, *J. Org. Chem.*, 2013, **78**, 6890.
[4] Y. Basel, A. Hassner, *J. Org. Chem.*, 2000, **65**, 6368.

^{1}H NMR ($CDCl_3$, 500 MHz) δ 7.32 (dd, *J* = 8.0, 8.0 Hz, 2H), 7.23 (d, *J* = 8.0 Hz, 2H), 7.16 (t, *J* = 7.5 Hz, 1H), 3.26 (s, 3H), 1.45 (s, 9H); ^{13}C NMR ($CDCl_3$, 125 MHz) δ 154.8, 143.8, 128.5, 125.5, 125.3, 80.2, 37.3, 28.3.

(3) Investigation of the formation of copper-carbamate species 6n:

a) Reaction of isocyanate 2n with peroxide 3 and copper(I) complex:

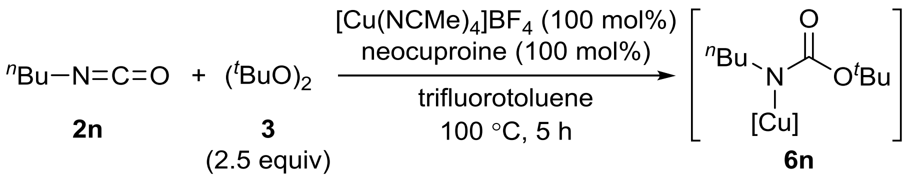

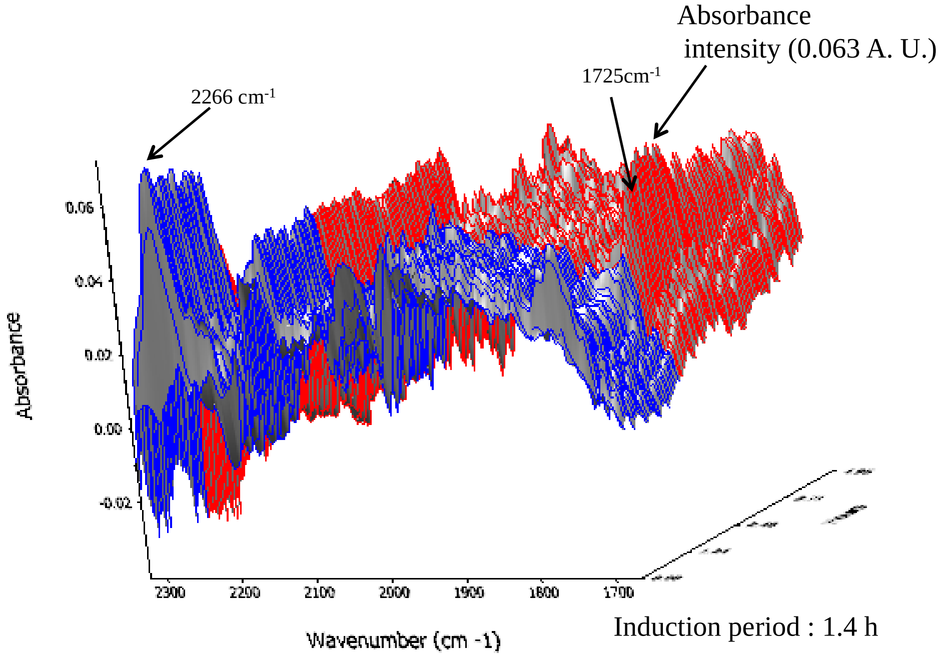

Figure S3. IR spectra (FT-IR waterfall plots) for the reaction between *n*-butyl isocyanate (**2n**) and di-*tert*-butyl peroxide (**3**) in the presence of [Cu(NCMe)]BF_4 (100 mol%) and neocuproine (100 mol%) in trifluorotoluene at 100 °C.

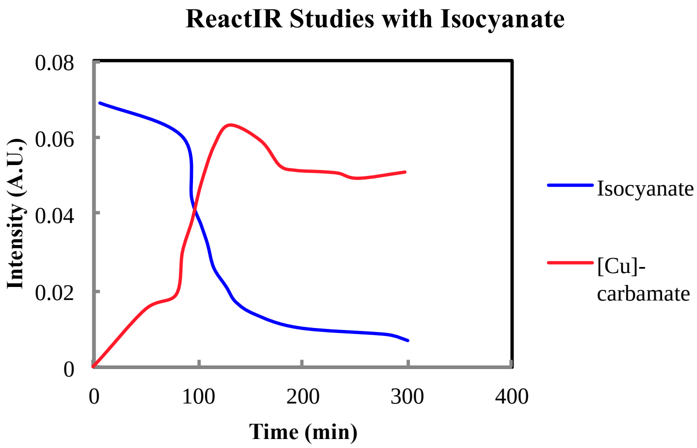

Figure S4. Two-dimentional graphs of the consumption of isocyanate **2n** (blue line) at 2266 cm^{-1} and formation of [Cu]-carbamate species **6n** (red line) at 1725 cm^{-1}.

Experimental procedure: To an oven dried two-necked 20 mL round bottom-flask, tetrakis(acetonitrile)copper(I) tetrafluoroborate $[Cu(NCMe)_4]BF_4$ (157 mg, 0.500 mmol, 100 mol%) and neocuproine (104 mg, 0.500 mmol, 100 mol%) were added in a glove box. The flask was removed from the glove box, di-*tert*-butyl peroxide (**3**, 182 mg, 0.23 mL 1.25 mmol, 2.5 equiv) and triflurotoluene (3.0 mL) were added under argon atmosphere. *n*-Butyl isocyanate (**2n**, 49.6 mg, 56.3 μL, 0.500 mmol) was added to the reaction mixture, and allowed to stir at room temperature with continuous recording by React IR. The flask was placed in a preheated oil bath and the mixture was stirred at 100 °C for 5 h with continuous recording by FT-IR (30 seconds/scan). An absorption band at 2266 cm^{-1} (assigned for a carbonyl group of isocyanate **2n**) gradually disappeared after 1.4 h (induction period), and a new absorbance band was observed at 1725 cm^{-1}. The maximum absorbance intensity at 1725 cm^{-1} was 0.063 (A.U.).

b) Reaction of *n*-butyl carbamate (5n) with peroxide (3) and copper(I) complex:

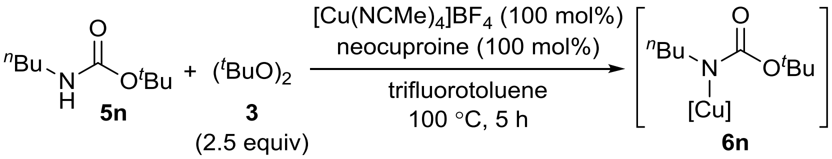

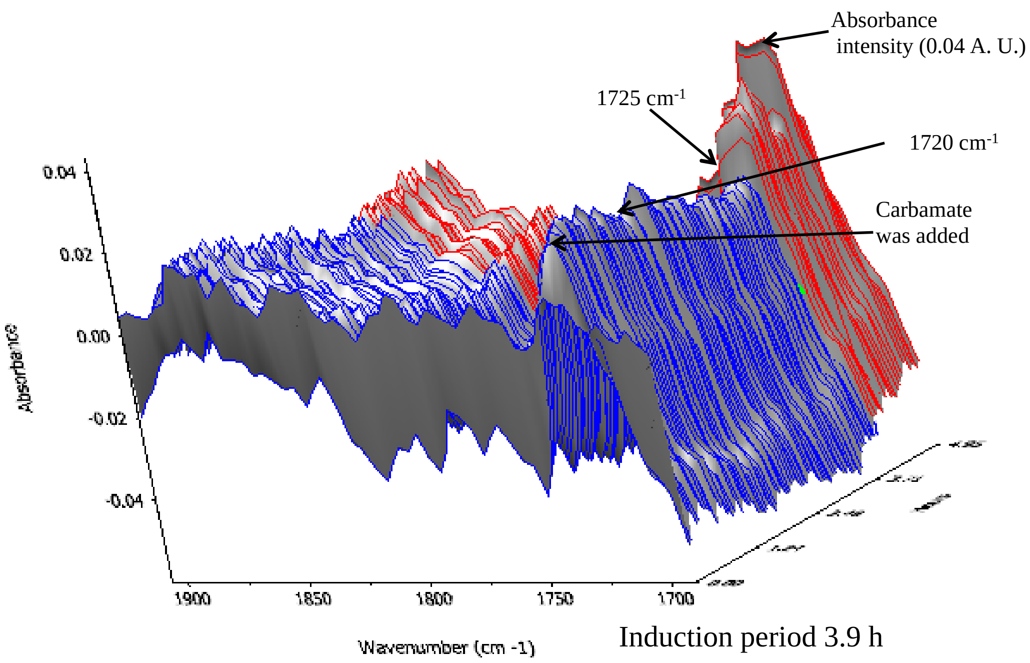

Figure S5. IR spectra (FT-IR waterfall plots) for the reaction between *n*-butyl carbamate (**5n**) and *tert*-butyl peroxide (**3**) in the presence of [Cu(NCMe)]BF_4 and neocuproine in trifluorotoluene at 100 °C

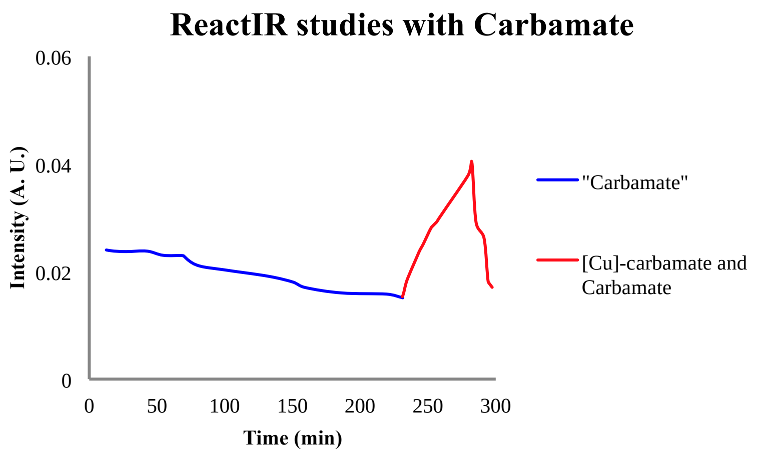

Figure S6. Two-dimentional graphs of the consumption of carbamate **5n** (blue line) and formation of [Cu]-carbamate species **6n** (red line) at 1725 cm^{-1}.

Experimental procedure: To an oven dried two-necked 20 mL round bottom-flask, tetrakis(acetonitrile)copper(I) tetrafluoroborate [Cu(NCMe)$_4$]BF_4 (157 mg, 0.500 mmol, 100 mol%) and neocuproine (104 mg, 0.500 mmol, 100 mol%) were added in a glove

box. The flask was removed from the glove box, di-*tert*-butyl peroxide (**3**, 182 mg, 0.23 mL 1.25 mmol, 2.5 equiv) and triflurotoluene (1.5 mL) were added under argon atmosphere. The reaction mixture was allowed to stir at room temperature followed by addition of *n*-butyl carbamate (**5**, 86.6 mg, 0.500 mmol) dissolved in trifluorotoluene (1.5 mL) and recorded by React IR. The flask was placed in a preheated oil bath and the mixture was stirred at 100 °C for 5 h with continuous recording by FT-IR (30 seconds/scan). An absorbance band for a carbonyl group of carbamate **5n** was observed at 1720 cm^{-1}. At the initial stage of the reaction, it was impossible to observe an absorbance peak of a carbonyl group of [Cu]-carbamate species **6n** at 1725 cm^{-1} because of the overlapping with the absorbance of carbamate **5n**. The absorbance of **6n** was clearly observed after 3.9 h (induction period). The observed maximum absorbance intensity at 1725 cm^{-1} was 0.040 (A.U., the absorbance intensity of **6n** must be less than 0.040 because of the overlapping with an absorbance of **5n**).

The differences of the induction periods and absorbance intensities of the bands at 1725 cm^{-1} for both reactions were correlated to the results that *n*-butyl isocyanate (**2n**) was more reactive compared with *n*-butyl carbamate **5n**.

(4) Difference of the reactivity between isocyanates and *n*-butyl carbamates towards carbamation of cyclohexane:

The difference of the reactivity between isocyanates and carbamates is possibly due to the different modes of the formation of [Cu]-carbamate species. The plausible reaction mechanisms are as shown below.

a) Formation of [Cu]-carbamate species from copper(I)-neocuproine complex, isocyanate, and *tert*-butyl peroxide:

A reaction of cyclohexane (**1a**) with phenylisocyanate (**2a**) was almost completely inhibited by 2,2,6,6-tetramethylpiperidine 1-oxyl (TEMPO, 2 equivalents) under the optimized conditions. This result indicated that the reaction would proceed in a radical pathway, and the active Cu-carbamate intermediate was formed via i) formation of a ^{t}BuO radical from (^{t}BuO)$_2$, ii) formation of a carbamate radical by addition of the *in situ* generated ^{t}BuO radical to an isocyanate, and iii) formation of the Cu(II)-carbamate species **6** by addition of the generated carbamate radical to Cu(I) catalyst.

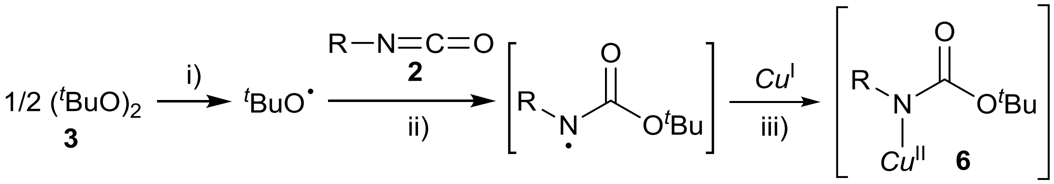

Scheme S1. Plausible mechanism for the formation of [Cu]-carbamate species from isocyanates.

b) Formation of [Cu]-carbamate species from copper(I)-neocuproine complex, secondary carbamate, and *tert*-butyl peroxide:

In the case of carbamates, the yield of the desired product was higher in *tert*-butyl phenylcarbamate (65%) than in *tert*-butyl butylcarbamate (11%). The difference of the yields may be due to the difference of acidities (NH proton of phenylcarbamate > NH proton of butylcarbamate).

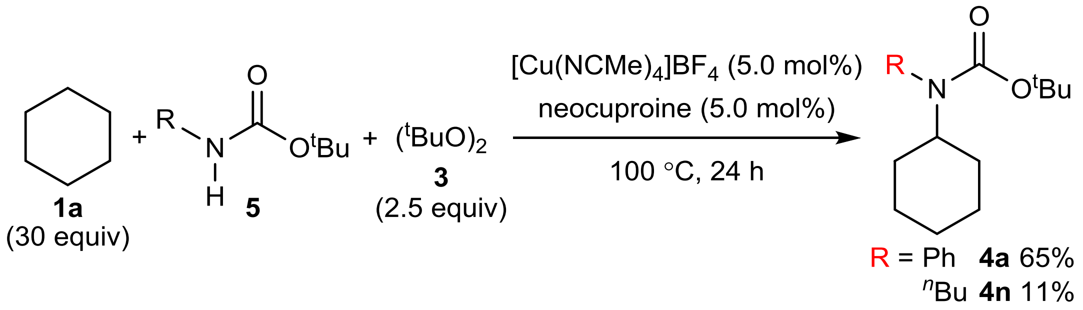

These results indicate the carbamate reaction proceeds via a Brønsted acid/base pathway, and the active Cu-carbamate intermediate was generated via i) formation of Cu(II)-O^{t}Bu species by oxidation of a Cu(I) catalyst with (^{t}BuO)$_2$ and ii) formation of the Cu(II)-carbamate intermediate **6** by deprotonation of a carbamate with the Cu(II)-O^{t}Bu species.

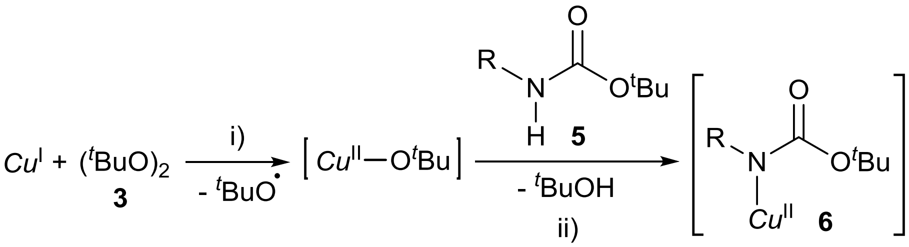

Scheme S2. Plausible mechanism for the formation of [Cu]-carbamate species from carbamates.

A possible reason for the observed difference in the induction period between the two reactions whether using isocyanates or carbamates might be due to the faster reaction rate in the addition of ^{t}BuO radical to isocyanates to generate presumed active copper(II)-carbamate than deprotonation of carbamates by Cu(II)-O^{t}Bu species.

8. Gram Scale Experiment

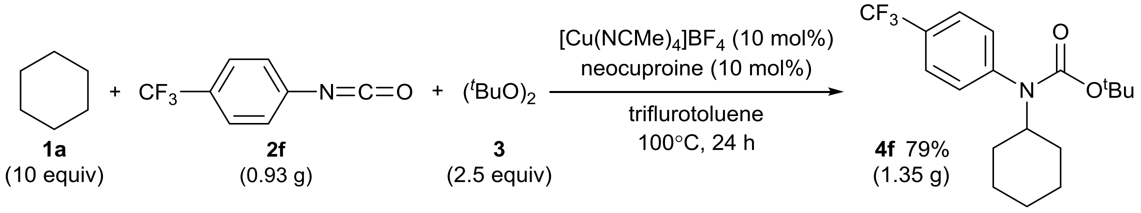

In a nitrogen-filled glove box, a flame-dried 50 mL screw cap test tube with a magnetic stir-bar was charged with tetrakis(acetonitrile)copper(I) tetrafluoroborate $[Cu(NCMe)_4]BF_4$ (157 mg, 0.500 mmol, 10 mol%) and neocuproine (104 mg, 0.500 mmol, 10 mol%). The test tube was removed from the glovebox, cyclohexane (**1a**, 4.2 g, 5.4 mL, 50.0 mmol, 10 equiv), trifluorotoluene (5.0 mL), and di-*tert*-butyl peroxide (**3**, 1.83 g, 2.3 mL, 12.5 mmol, 2.5 equiv) were added under argon atmosphere, and the mixture was stirred at room temperature for 10 minutes. 4-(Trifluoromethyl)phenyl isocyanate (**2f,** 0.935 g, 5.00 mmol) was added to the mixture and the reaction vessel was placed in a pre-heated oil bath. Then, the mixture was stirred at 100 °C for about 24 h. The reaction mixture was allowed to cool to room temperature, concentrated under reduced pressure, and purified by column chromatography on silica gel (hexane/ethyl acetate = 19:1) to give the desired tertiary carbamate **4f** as a white solid (1.35 g, 79%).

9. Kinetic Isotopic Effect (KIE) Experiments

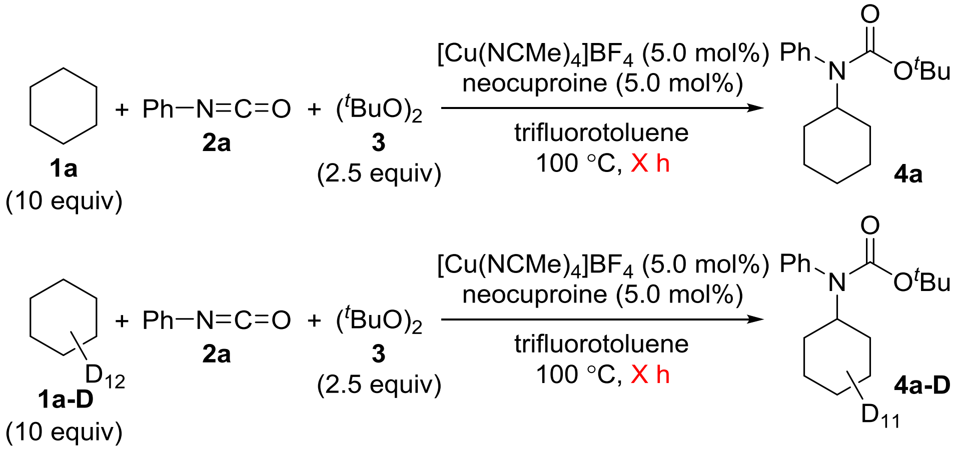

In a nitrogen-filled glove box, flame-dried 10 mL screw cap test tubes with a magnetic stir-bar were charged with tetrakis(acetonitrile) copper(I) tetrafluoroborate $[Cu(NCMe)_4]BF_4$ (7.9 mg, 0.0250 mmol, 5.0 mol%) and neocuproine (5.0 mg, 0.0250 mmol, 5.0 mol%). The test tubes were removed from the glove box, cyclohexane (**1a**, 420 mg, 5.00 mmol, 10 equiv), trifluorotoluene (0.50 mL), di-*tert*-butyl peroxide (**3**, 183 mg, 1.25 mmol, 2.5 equiv), and phenyl isocyanate (**2a**, 59.6 mg, 54.3 µL, 0.500 mmol) were added under argon atmosphere. All the test tubes were placed in a preheated oil bath and the mixtures were stirred vigorously at 100 °C. The vials were removed at different designated time interval (0, 30, 40, 70, and 90 min). The reaction mixture was cooled to room temperature, and passed through a short pad of silica gel with the aid of dichloromethane. The reaction mixture was concentrated and dodecane (85.2 mg, 0.500 mmol) was added as an internal standard. The desired product was quantified through gas chromatography.

By using above procedure, the similar sets of experiments were conducted using cyclohexane-d_{12} instead of cyclohexane.

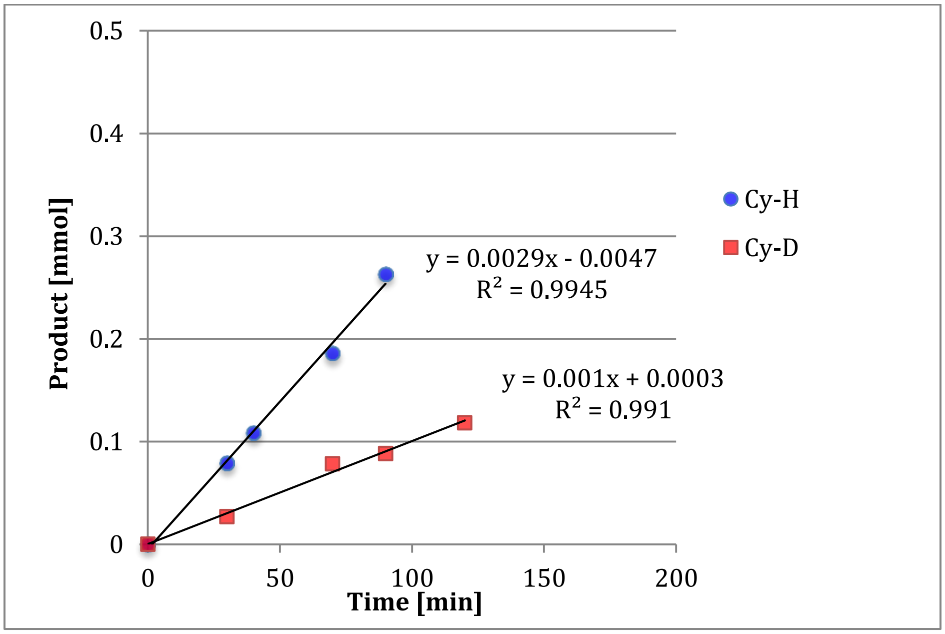

Figure S2. Rate of carbamation of cyclohexane (Cy-H) and deuterated cyclohexane (Cy-D)

Based on the above results, the KIE value is k_H/k_D = 2.9, which suggested that C-H bond cleavage was the rate-determining step of the reaction.

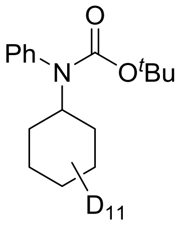

***tert*-Butyl (cyclohexyl-d_{11})(phenyl)carbamate (4a-D).** Purified by silica gel column chromatography (hexane/ethyl acetate = 19:1); R_f = 0.37 (UV active, hexane/ethyl acetate = 9:1); 50% (procedure A, 24 h); colorless liquid; ^{1}H NMR ($CDCl_3$, 500 MHz) δ 7.32 (dd, *J* = 7.5, 7.5 Hz, 2H), 7.27-7.24 (m, 1H), 7.05 (d, *J* = 7.5 Hz, 2H), 1.35 (s, 9H); ^{13}C NMR ($CDCl_3$, 125 MHz) δ 154.9, 139.5, 129.9, 128.3, 126.8, 79.4, 55.7 (t, *J* = 19.0 Hz), 30.9 (quint, *J* = 19.0 Hz), 28.3, 24.7 (quint, *J* = 19.0 Hz), 24.0 (quint, *J* = 19.0 Hz); IR (thin film) ν 3011, 2978, 2360, 2208, 2106, 1683, 1364, 1167 cm^{-1}; HRMS (ESI) calcd for $C_{17}H_{14}D_{11}NO_2$ (*m/z*) 309.2473 $[M+Na]^+$ Found 309.2464.

10. Copper-Catalyzed Amination of Cyclohexane

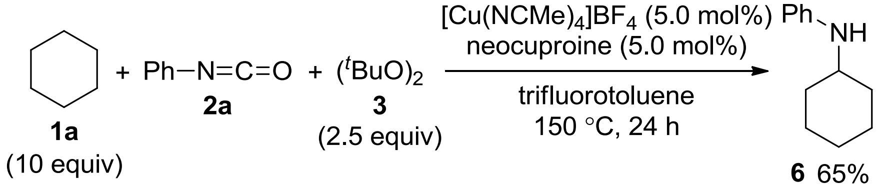

In a nitrogen-filled glove box, a flame-dried 10 mL screw cap test tube with a magnetic stir-bar was charged with tetrakis(acetonitrile)copper(I) tetrafluoroborate $[Cu(NCMe)_4]BF_4$ (7.9 mg, 0.0250 mmol, 5.0 mol%) and neocuproine (5.0 mg, 0.0250 mmol, 5.0 mol%). The test tube was removed from the glove box. Cyclohexane (**1a**, 420 mg, 5.00 mmol, 10 equiv), trifluorotoluene (1.0 mL), di-*tert*-butyl peroxide (**3**, 183 mg, 1.25 mmol, 2.5 equiv), and phenyl isocyanate (**2a**, 59.6 mg, 0.500 mmol) were added under argon atmosphere. The test tube was placed in a preheated oil bath, and the mixture was stirred vigorously at 150 °C for 24 h, cooled to room temperature, concentrated under reduced pressure, and purified by column chromatography on silica gel (hexane/ethyl acetate = 19:1) to give the desired *N*-cyclohexylaniline (**6)** as a light brown liquid. R_f = 0.46 (UV active, hexane/ethyl acetate = 9:1); 65%; The spectral data of **6** were in accord with the literature report.[5] ^{1}H NMR ($CDCl_3$, 500 MHz) δ 7.15 (t, J = 7.45 Hz, 2H), 6.66 (t, J = 7.45 Hz, 1H), 6.6 (d, J = 7.45 Hz, 2H), 3.52 (brs, 1H), 3.28-3.23 (m, 1H), 2.10-2.02 (m, 2H), 1.81-1.71 (m, 2H), 1.69-1.61 (m, 1H), 1.43-1.29 (m, 2H), 1.27-1.06 (m, 3H); ^{13}C NMR ($CDCl_3$, 125 MHz) δ 147.3, 116.7, 113.1, 51.6, 33.4, 25.9, 24.9; IR (thin film) ν 3402, 3050, 3017, 2360, 1698, 1601, 1505 cm^{-1}; HRMS (ESI) calcd for $C_{12}H_{17}N$ (*m/z*) 198.1258 $[M+Na]^+$ Found 198.1251.

[5] (*a*) M. L. Kantam, G. T. Venkanna, C. Sridhar, B. Sreedhar, B. M. Choudary, *J. Org. Chem.*, 2006, **71**, 9522; (*b*) C.-T. Yang, Y. Fu, Y.-B. Huang, J. Yi, Q.-X. Guo and L. Liu, *Angew. Chem. Int. Ed.*, 2009, **48**, 7398.

11. ^{1}H and ^{13}C NMR Spectra

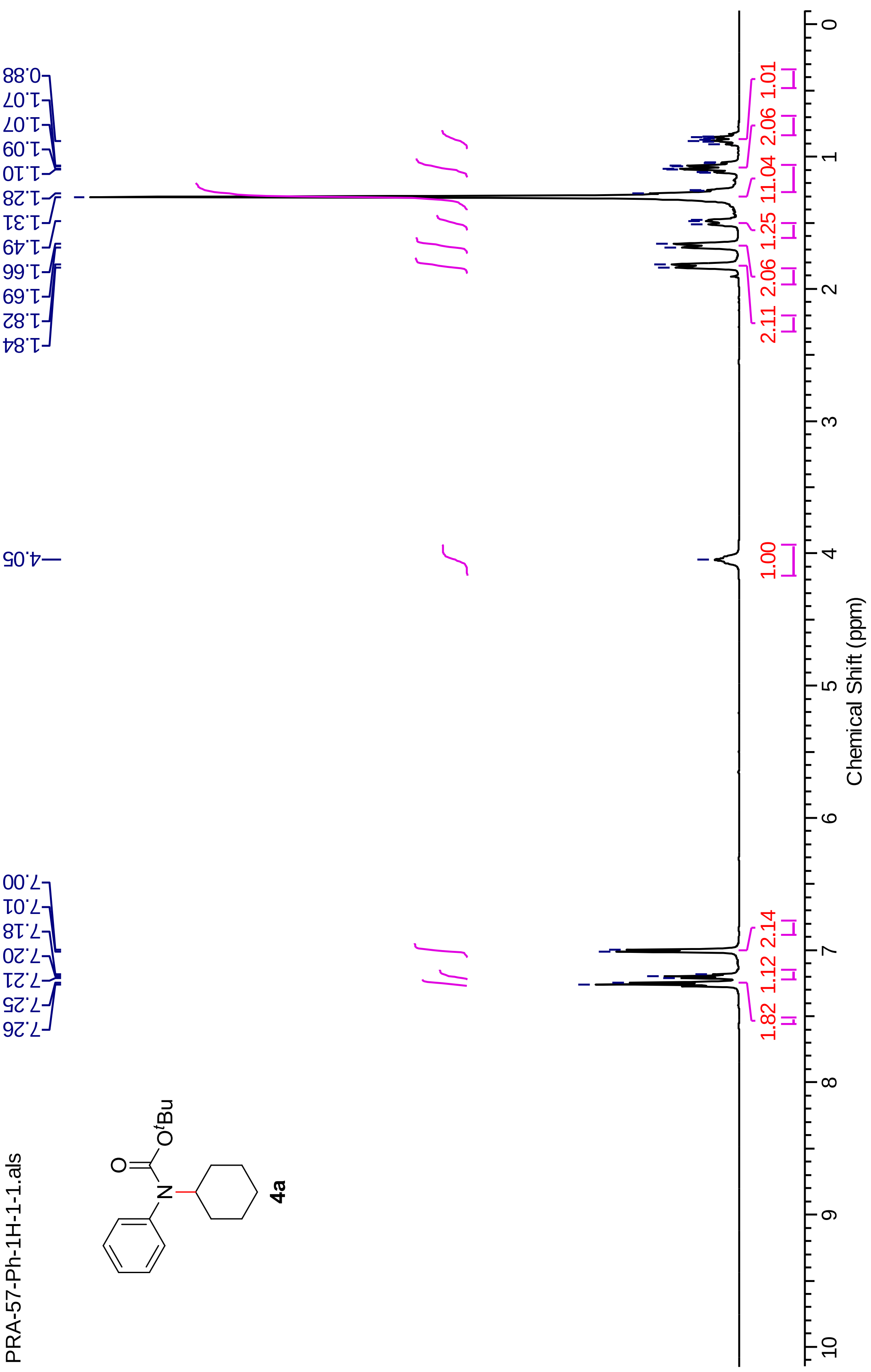
PRA-57-Ph-1H-1-1.als
O
N
O^{t}Bu
4a
7.26
7.25
7.21
7.20
7.18
7.01
7.00
4.05
1.84
1.82
1.69
1.66
1.49
1.31
1.28
1.10
1.09
1.07
1.07
0.88
1.82 1.12 2.14
1.00
2.11 2.06 1.25 11.04 2.06 1.01
Chemical Shift (ppm)
10 9 8 7 6 5 4 3 2 1 0

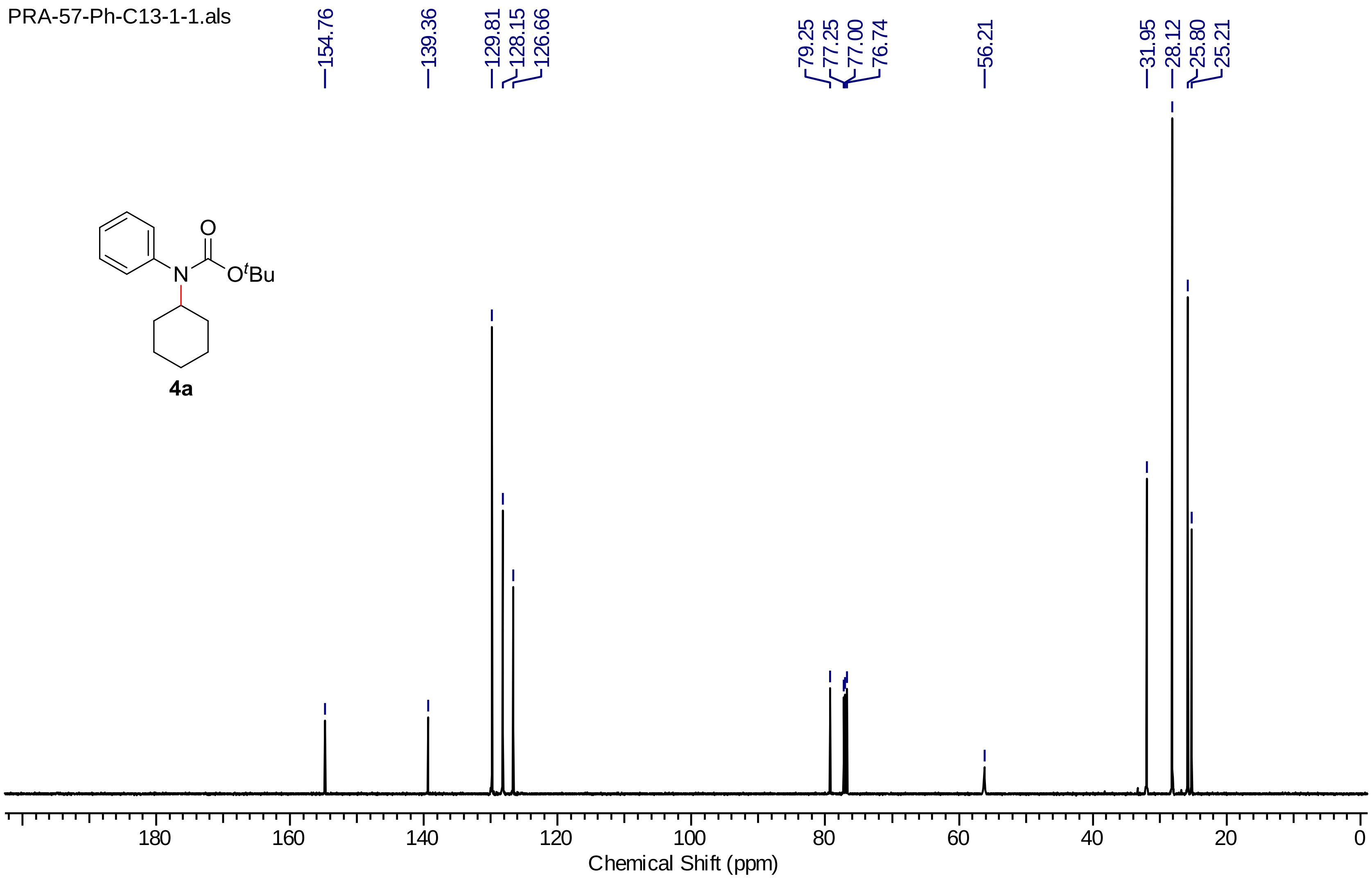
PRA-57-Ph-C13-1-1.als
154.76
139.36
129.81
128.15
126.66
79.25
77.25
77.00
76.74
56.21
31.95
28.12
25.80
25.21
O
N
O^{t}Bu
4a
180
160
140
120
100
80
60
40
20
0
Chemical Shift (ppm)

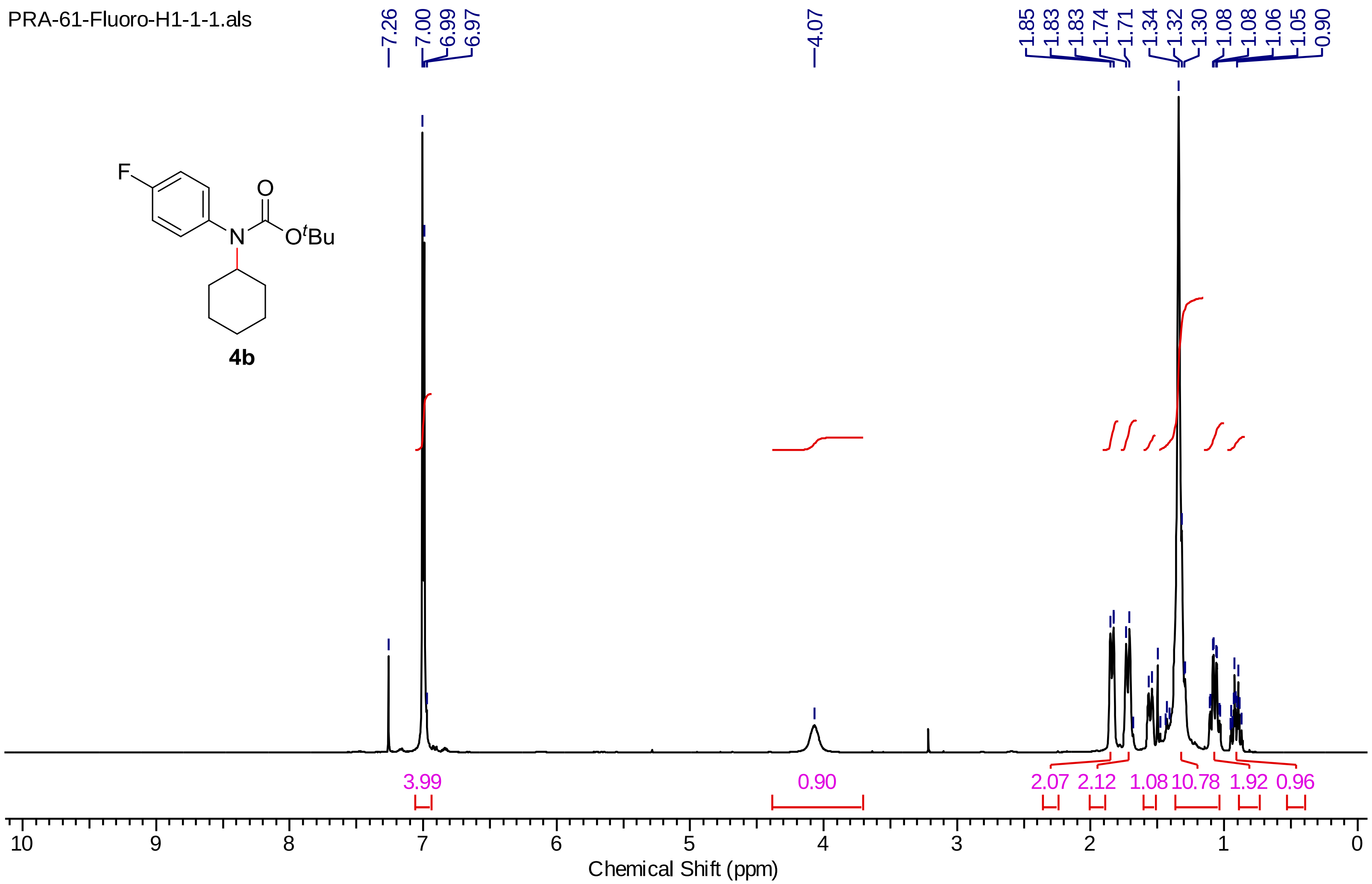
PRA-61-Fluoro-H1-1-1.als
F
N
O
O^{t}Bu
4b
7.26
7.00
6.99
6.97
4.07
1.85
1.83
1.83
1.74
1.71
1.34
1.32
1.30
1.08
1.08
1.06
1.05
0.90
3.99
0.90
2.07
2.12
1.08
10.78
1.92
0.96
10
9
8
7
6
5
4
3
2
1
0
Chemical Shift (ppm)

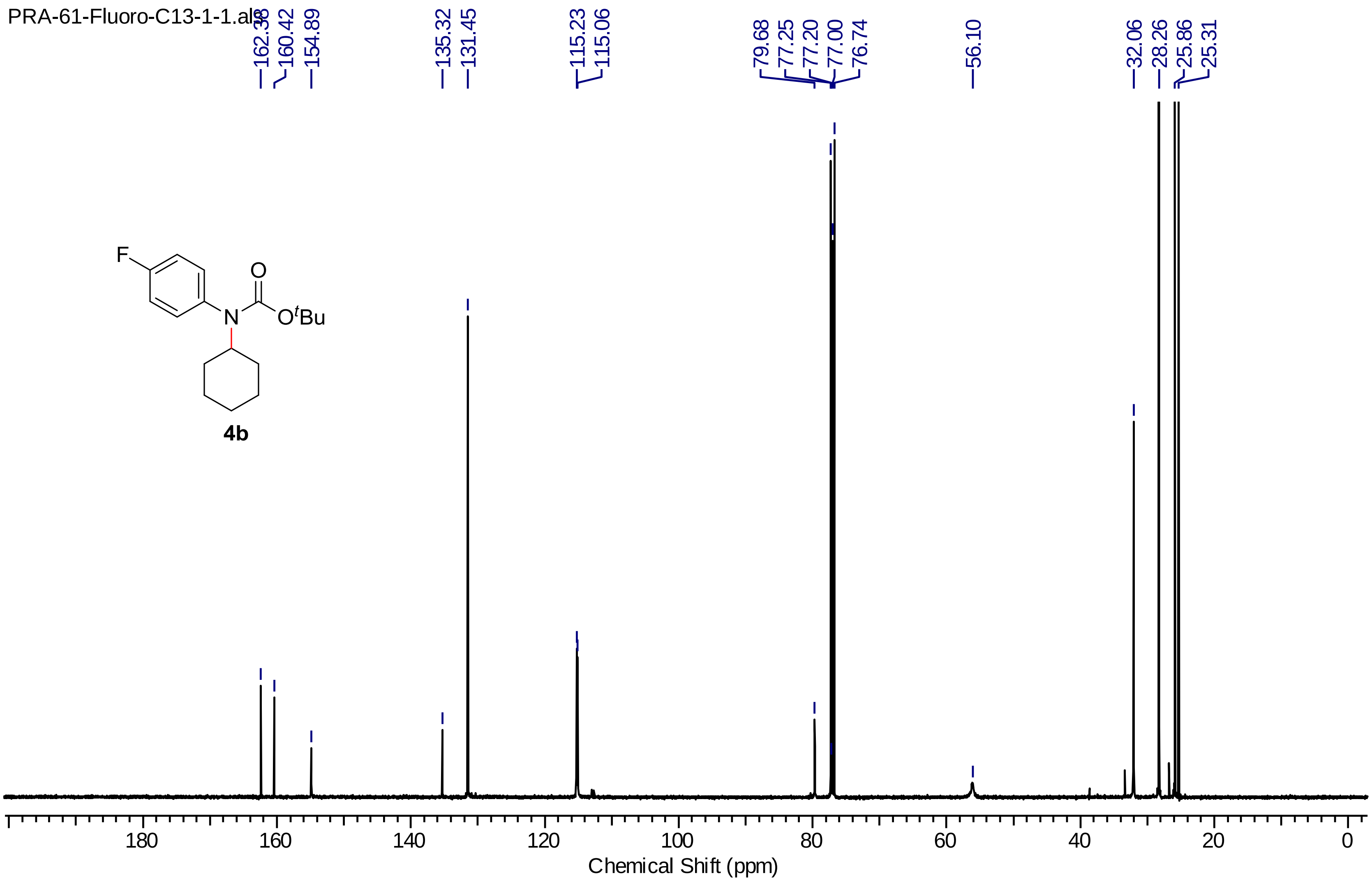

PRA-61-Fluoro-C13-1-1.als
162.38
160.42
154.89
135.32
131.45
115.23
115.06
79.68
77.25
77.20
77.00
76.74
56.10
32.06
28.26
25.86
25.31
F
N
O
O^{t}Bu
4b
180
160
140
120
100
80
60
40
20
0
Chemical Shift (ppm)

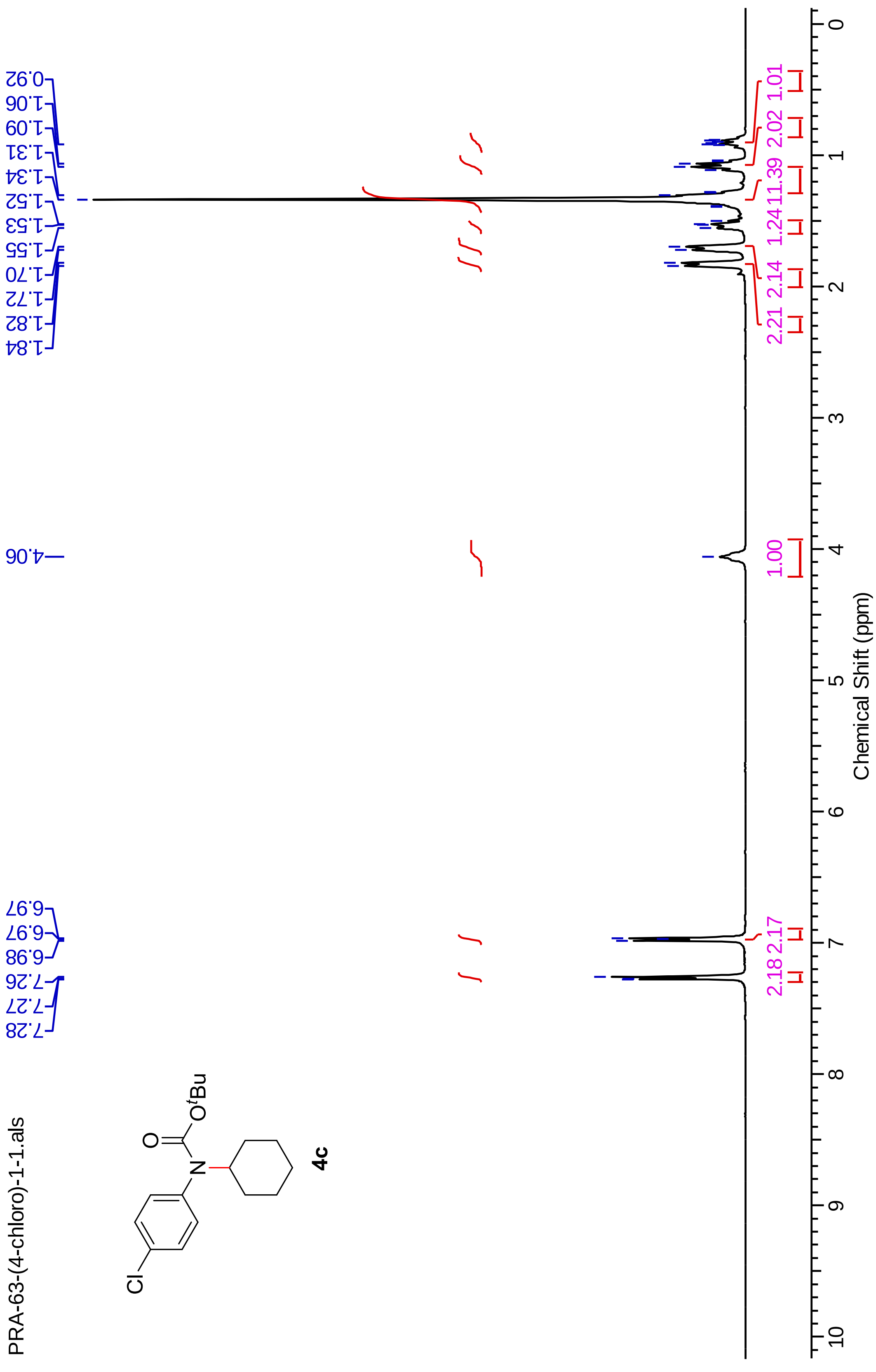
PRA-63-(4-chloro)-1-1.als
Cl
N
O
O^{t}Bu
4c
7.28
7.27
7.26
6.98
6.97
6.97
4.06
1.84
1.82
1.72
1.70
1.55
1.53
1.52
1.34
1.31
1.09
1.06
0.92
2.18
2.17
1.00
2.21
2.14
1.24
11.39
2.02
1.01
Chemical Shift (ppm)

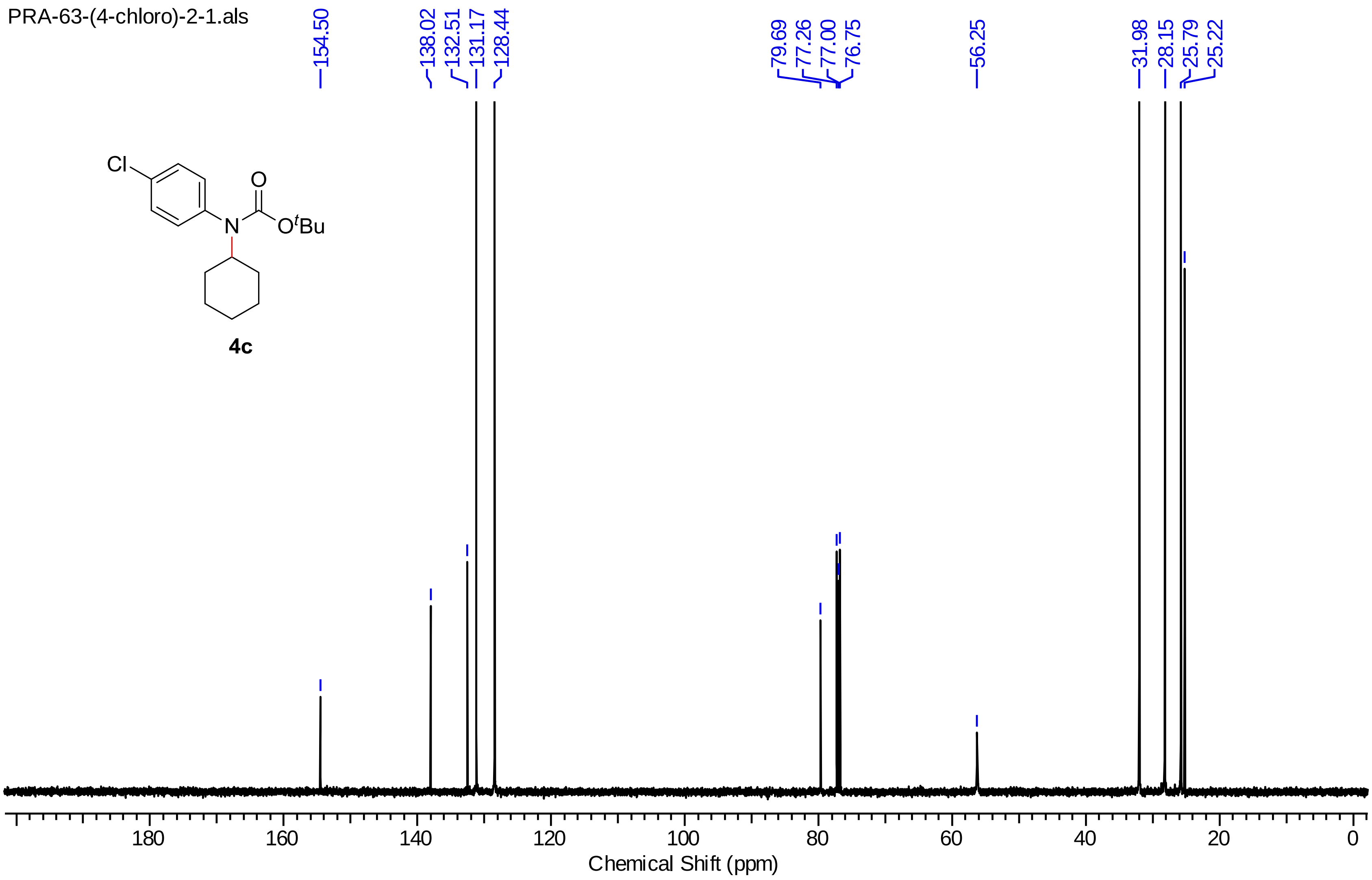
PRA-63-(4-chloro)-2-1.als
154.50
138.02
132.51
131.17
128.44
79.69
77.26
77.00
76.75
56.25
31.98
28.15
25.79
25.22
Cl
O
N
O^{t}Bu
4c
180
160
140
120
100
80
60
40
20
0
Chemical Shift (ppm)

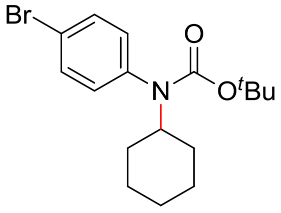
Br
O
N
O^{t}Bu

4d

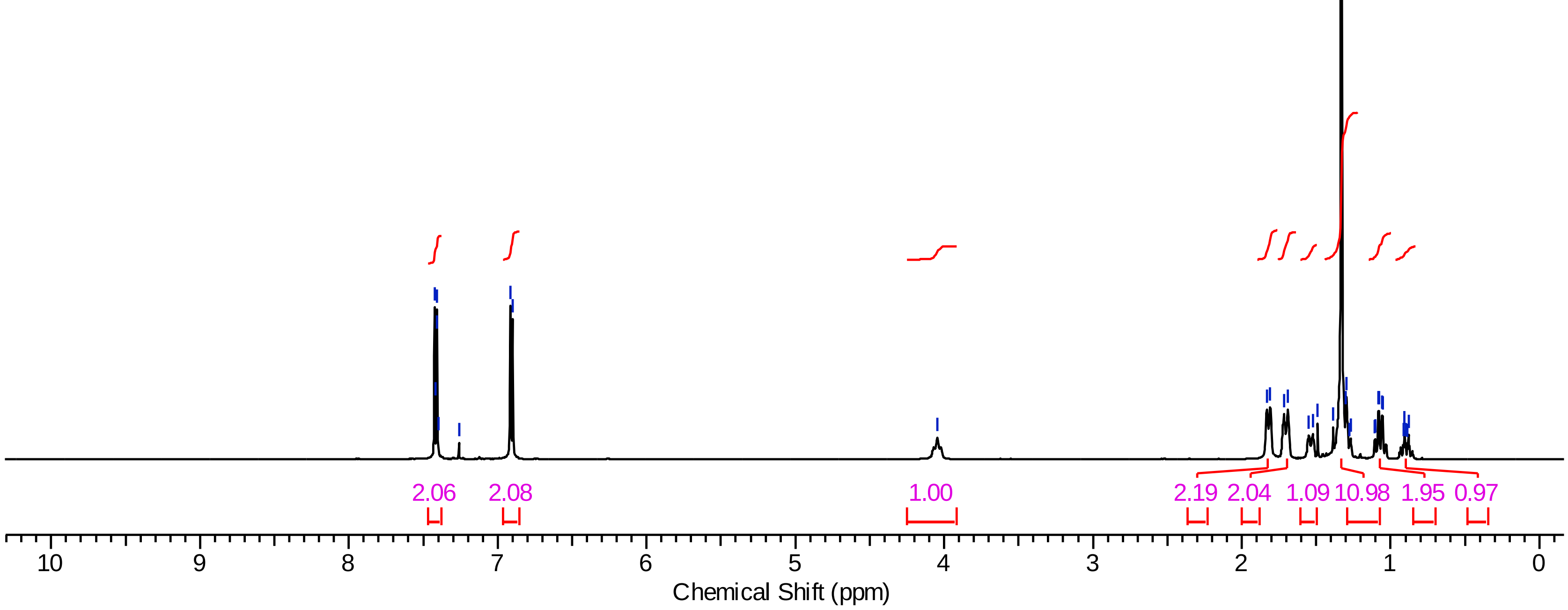
PRA-65(7-8)-p-bromophenyl-1-1.als
7.42
7.42
7.41
7.41
7.40
7.26
6.92
6.90
4.05
1.83
1.81
1.72
1.69
1.52
1.49
1.33
1.30
1.30
1.08
1.08
1.06
2.06
2.08
1.00
2.19
2.04
1.09
10.98
1.95
0.97
10
9
8
7
6
5
4
3
2
1
0
Chemical Shift (ppm)

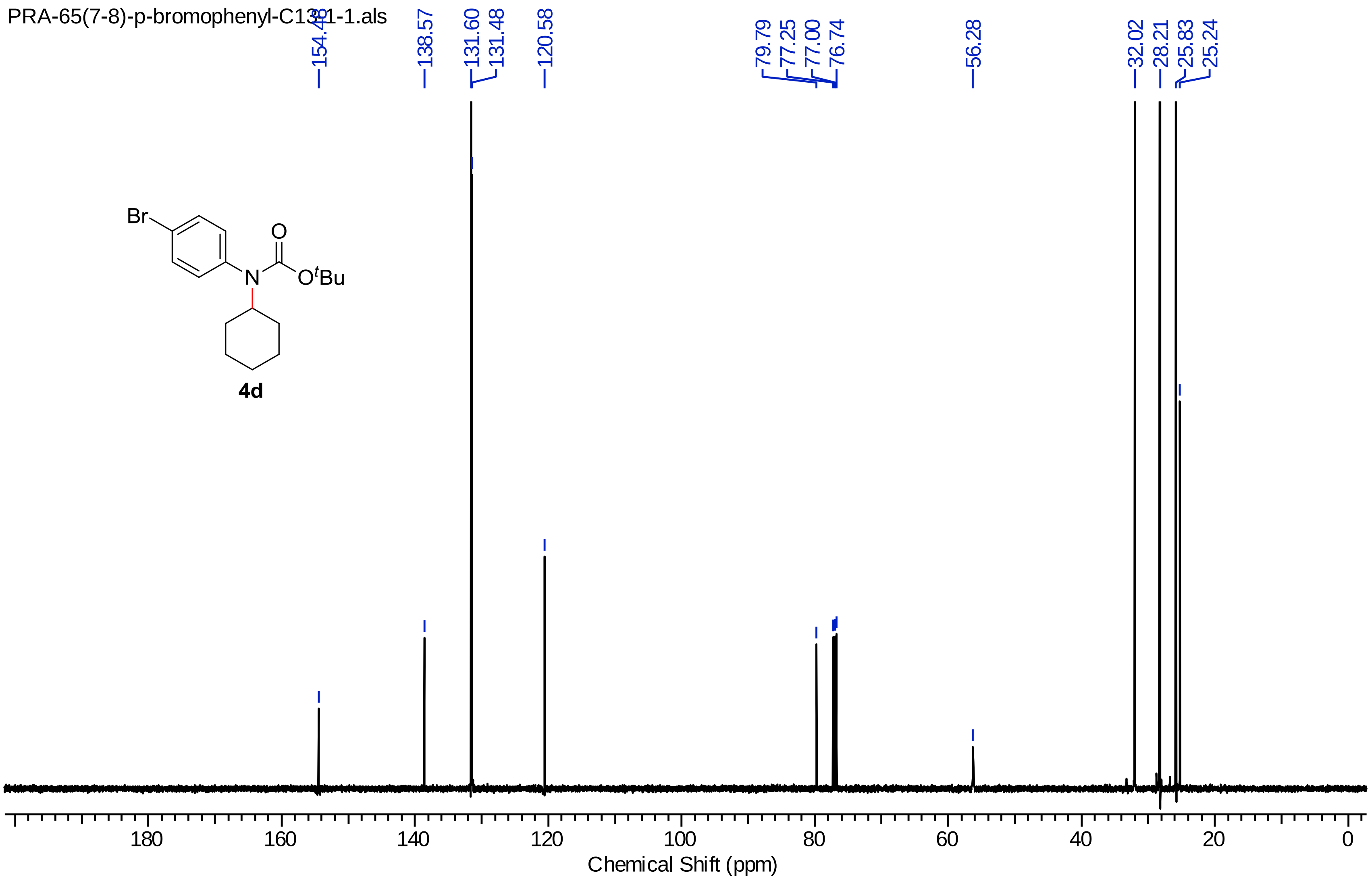
PRA-65(7-8)-p-bromophenyl-C13-1-1.als
154.48
138.57
131.60
131.48
120.58
79.79
77.25
77.00
76.74
56.28
32.02
28.21
25.83
25.24
Br
N
O
O^{t}Bu
4d
180
160
140
120
100
80
60
40
20
0
Chemical Shift (ppm)

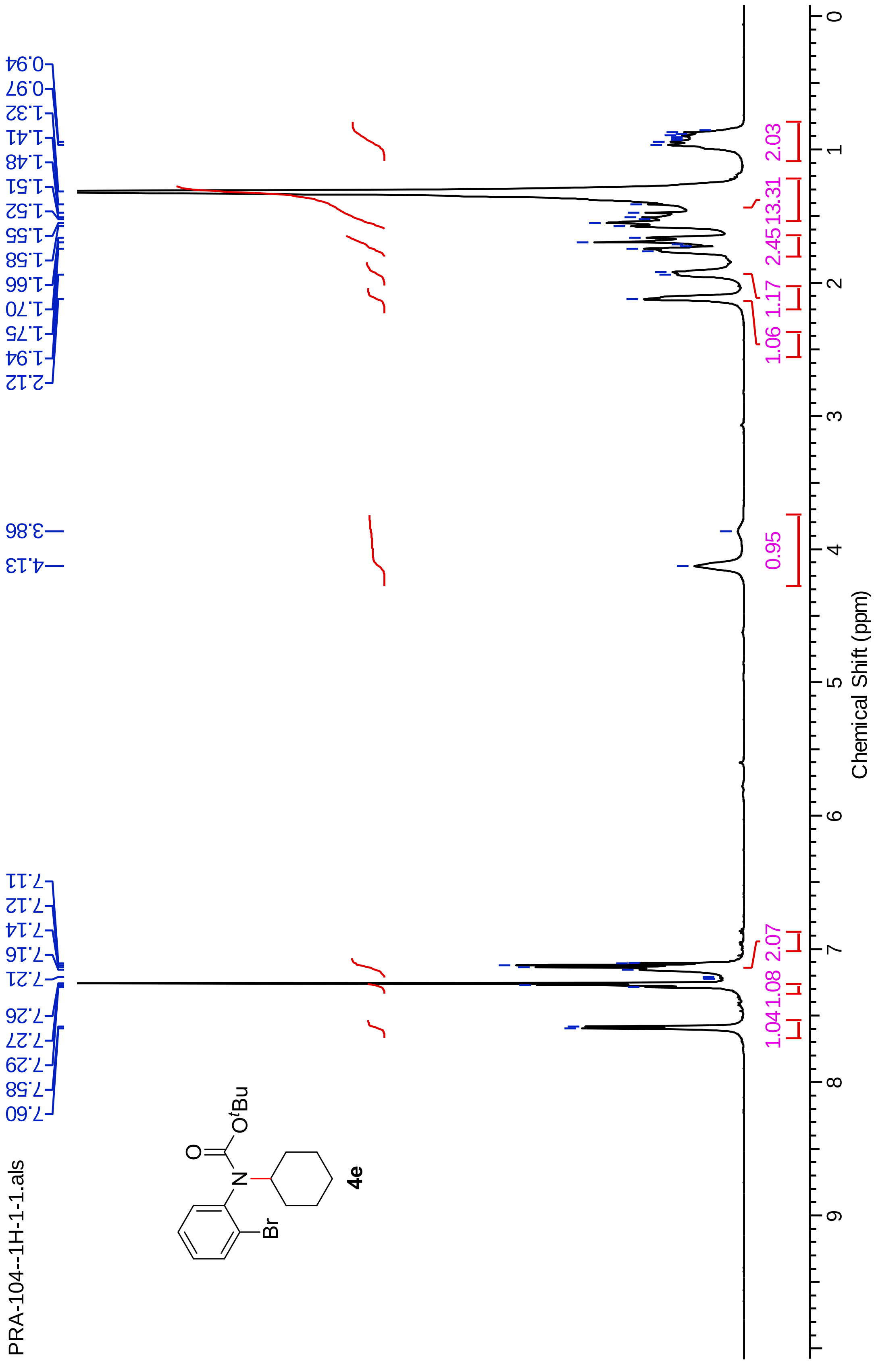

PRA-104--1H-1-1.als
4e
Br
N
O
O^{t}Bu
7.60
7.58
7.29
7.27
7.26
7.21
7.16
7.14
7.12
7.11
4.13
3.86
2.12
1.94
1.75
1.70
1.66
1.58
1.55
1.52
1.51
1.48
1.41
1.32
0.97
0.94
1.04
1.08
2.07
0.95
1.06
1.17
2.45
13.31
2.03
Chemical Shift (ppm)

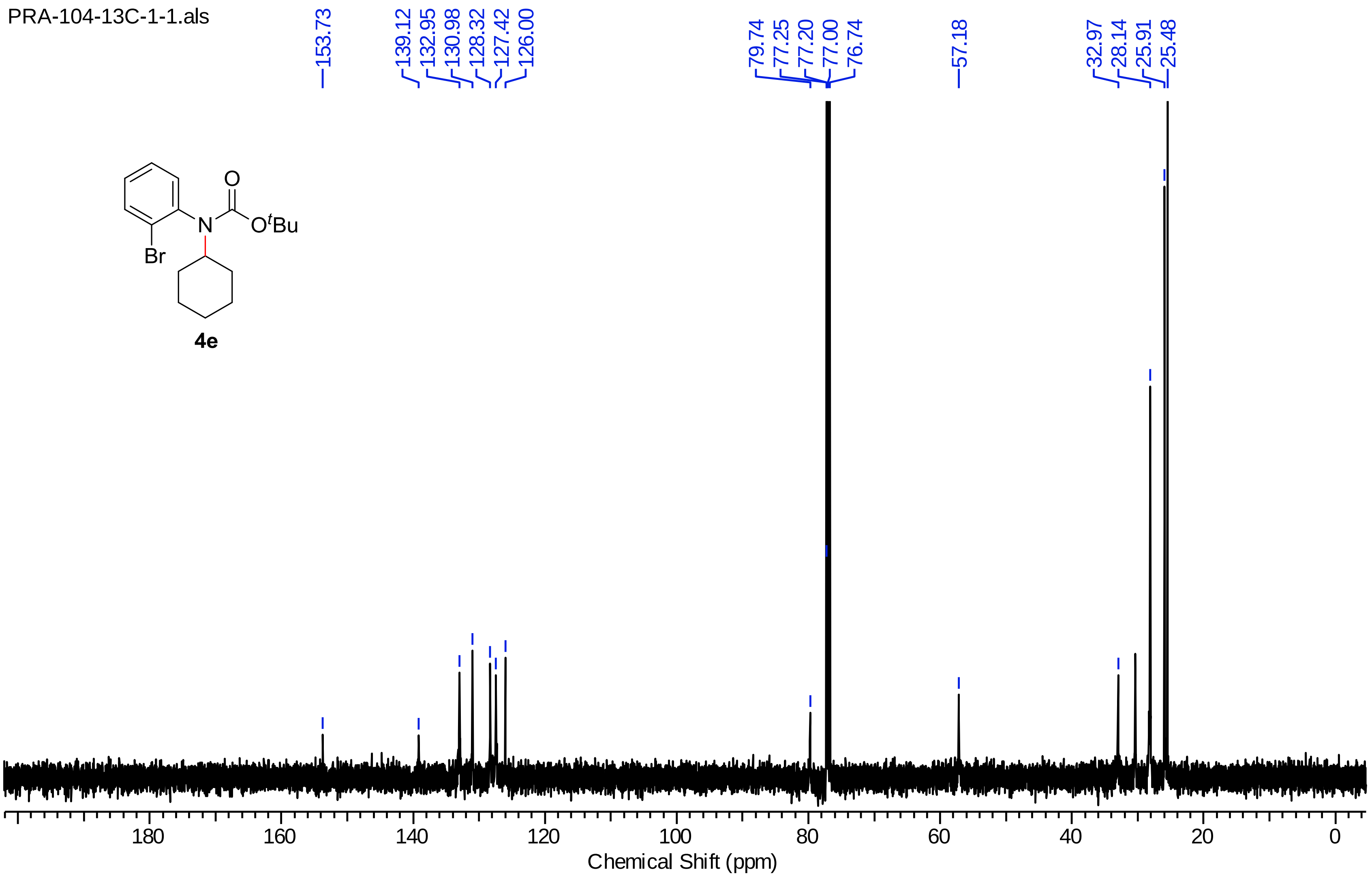

PRA-104-13C-1-1.als
153.73
139.12
132.95
130.98
128.32
127.42
126.00
79.74
77.25
77.20
77.00
76.74
57.18
32.97
28.14
25.91
25.48
O
N
O^{t}Bu
Br
4e
180
160
140
120
100
80
60
40
20
0
Chemical Shift (ppm)

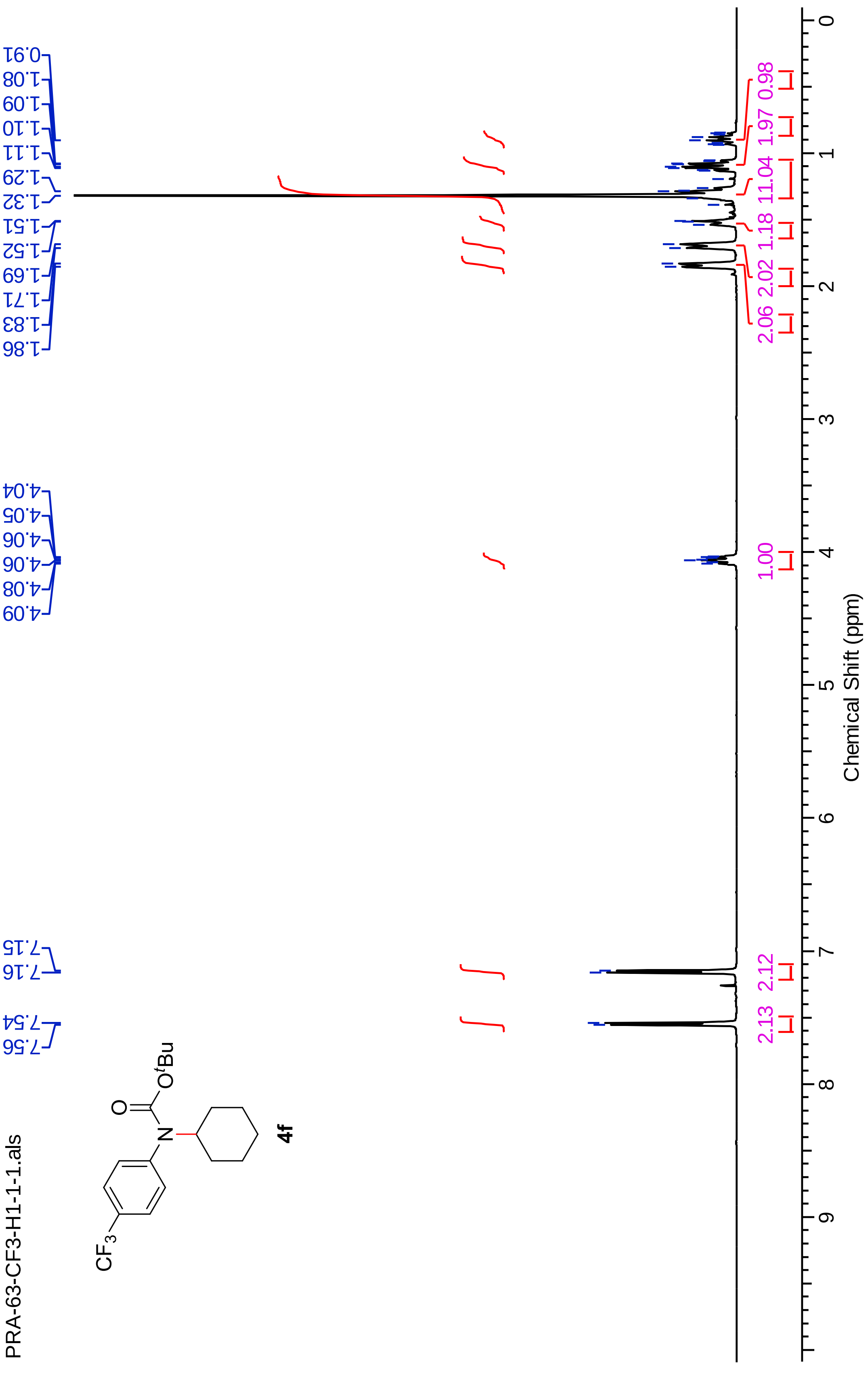

PRA-63-CF3-H1-1-1.als
CF3
N
O
O^tBu
4f
7.56
7.54
7.16
7.15
4.09
4.08
4.06
4.06
4.05
4.04
1.86
1.83
1.71
1.69
1.52
1.51
1.32
1.29
1.11
1.10
1.09
1.08
0.91
2.13
2.12
1.00
2.06
2.02
1.18
11.04
1.97
0.98
Chemical Shift (ppm)

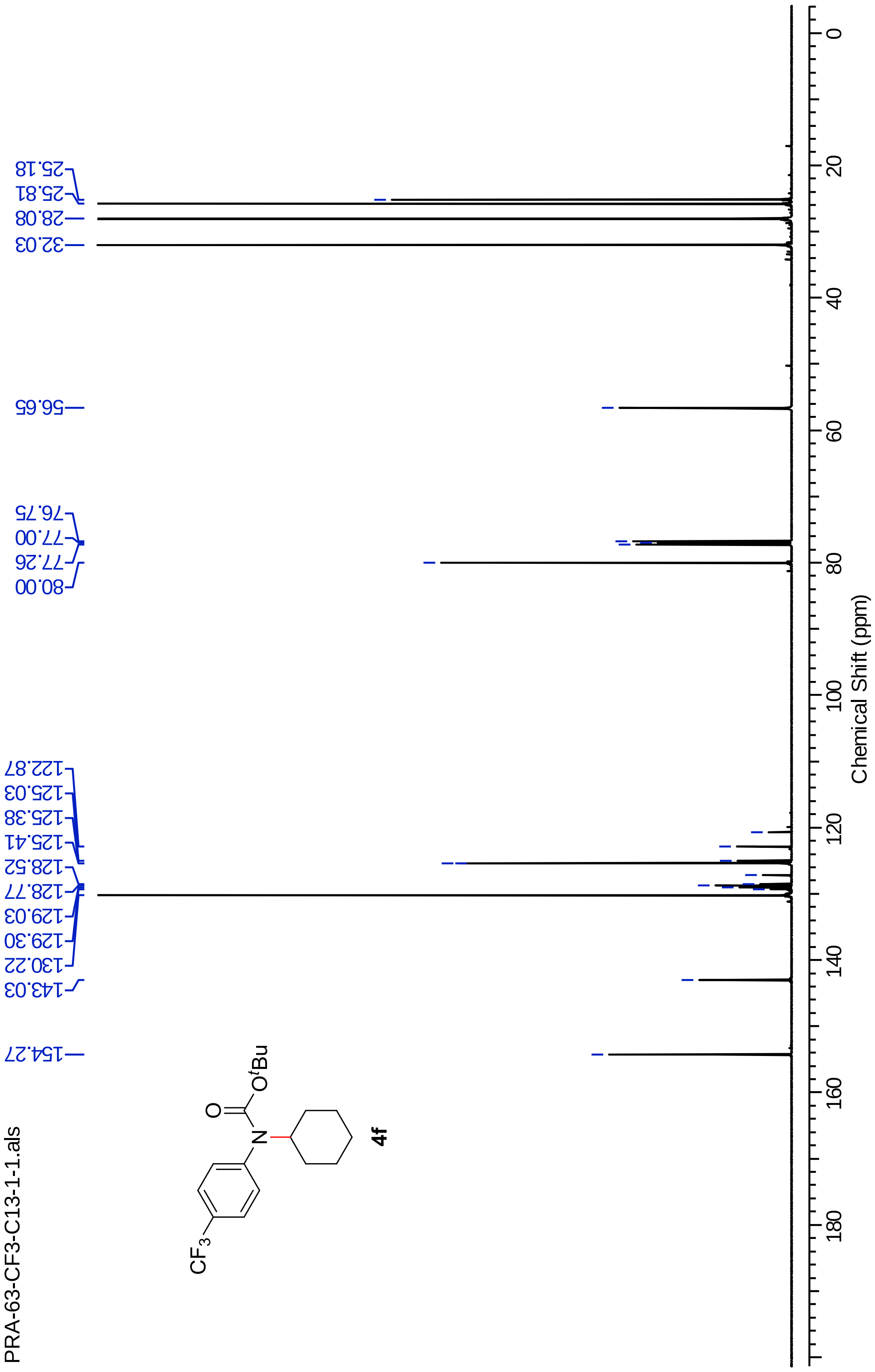
PRA-63-CF3-C13-1-1.als
CF3
N
O
O^tBu
4f
154.27
143.03
130.22
129.30
129.03
128.77
128.52
125.41
125.38
125.03
122.87
80.00
77.26
77.00
76.75
56.65
32.03
28.08
25.81
25.18
0
20
40
60
80
100
120
140
160
180
Chemical Shift (ppm)

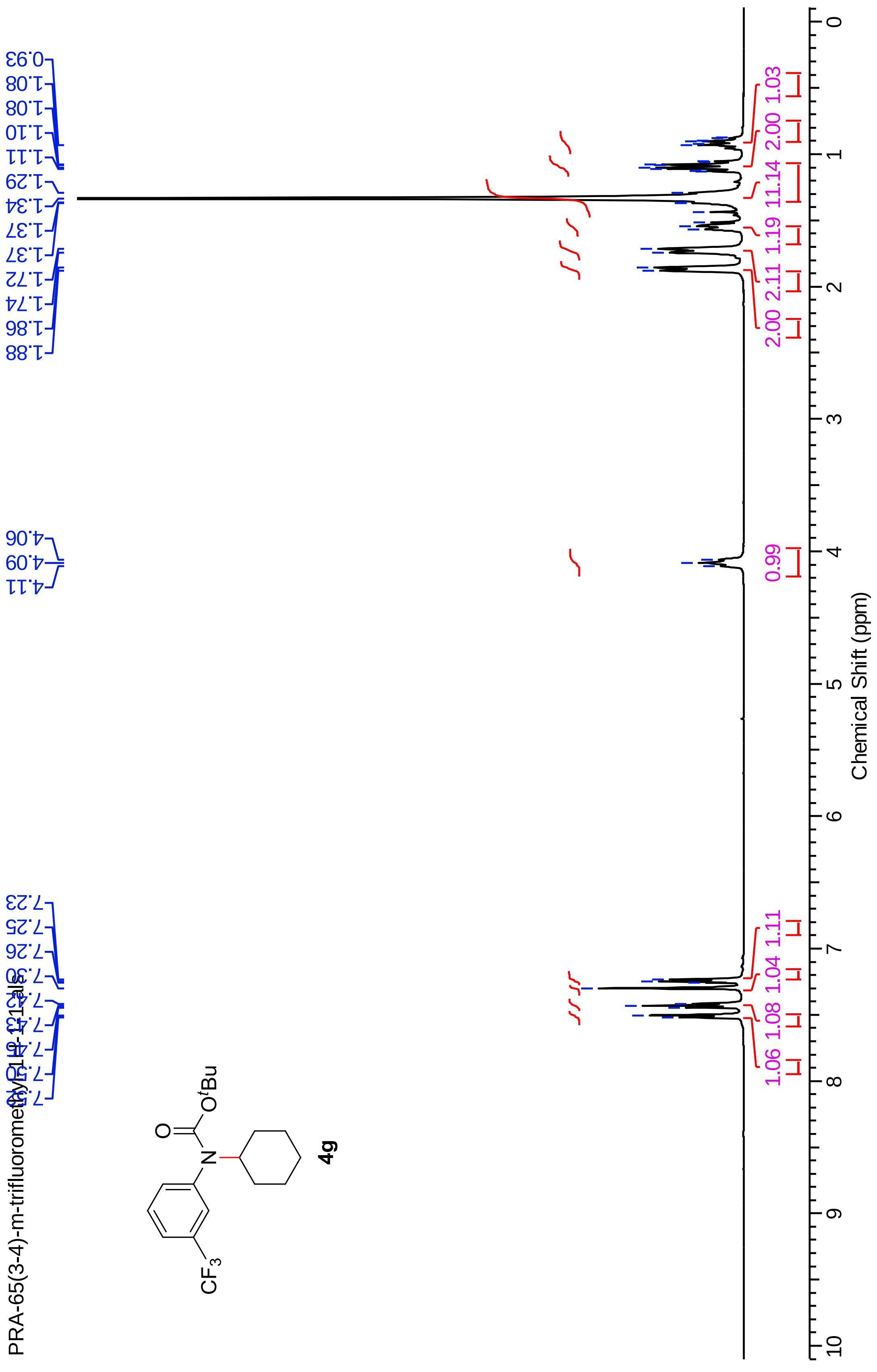
PRA-65(3-4)-m-trifluoromethyl
7.30
7.26
7.25
7.23
4.11
4.09
4.06
1.88
1.86
1.74
1.72
1.37
1.37
1.34
1.29
1.11
1.10
1.08
1.08
0.93
CF3
N
O
O^tBu
4g
1.06
1.08
1.04
1.11
0.99
2.00
2.11
1.19
11.14
2.00
1.03
10
9
8
7
6
5
4
3
2
1
0
Chemical Shift (ppm)

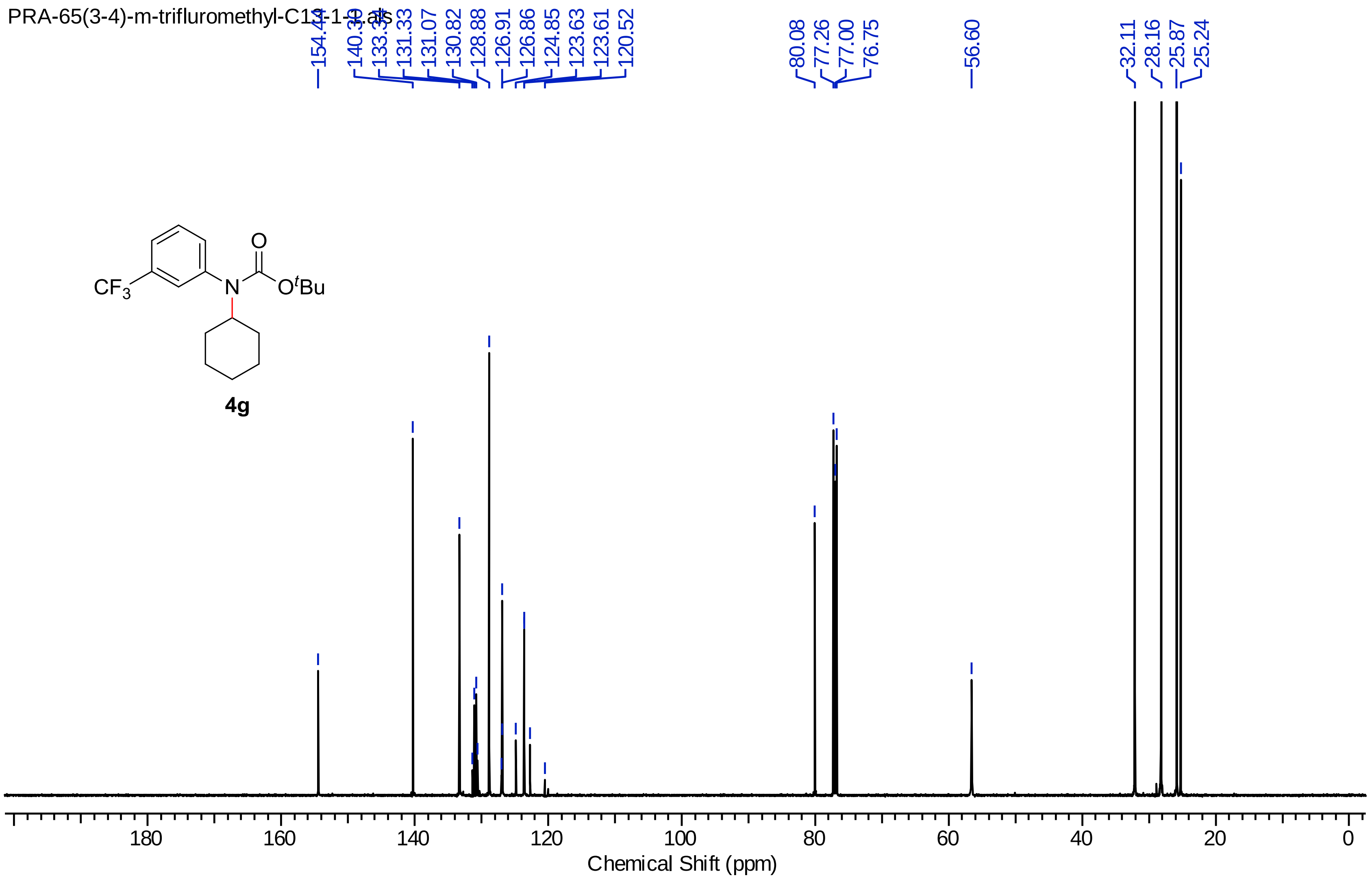
PRA-65(3-4)-m-triflurometh yl-C13-1-[illegible].als
154.44
140.30
133.3[illegible]
131.33
131.07
130.82
128.88
126.91
126.86
124.85
123.63
123.61
120.52
80.08
77.26
77.00
76.75
56.60
32.11
28.16
25.87
25.24
CF3
O
N
O^{t}Bu
4g
180
160
140
120
100
80
60
40
20
0
Chemical Shift (ppm)

PRA-61-OMe-1-1.als

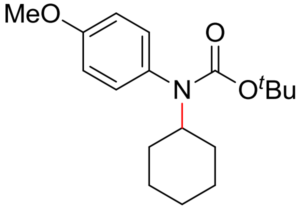

4h

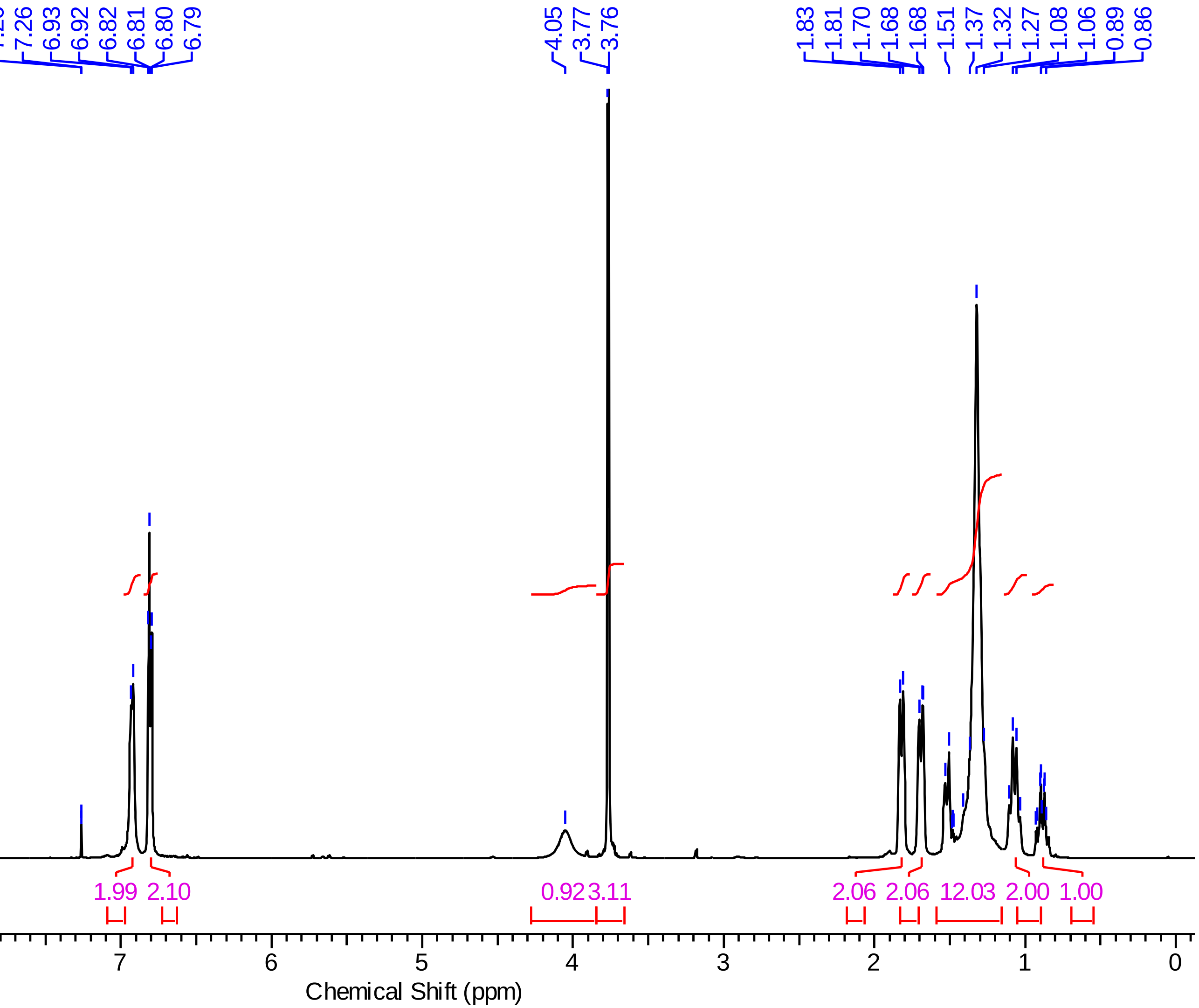

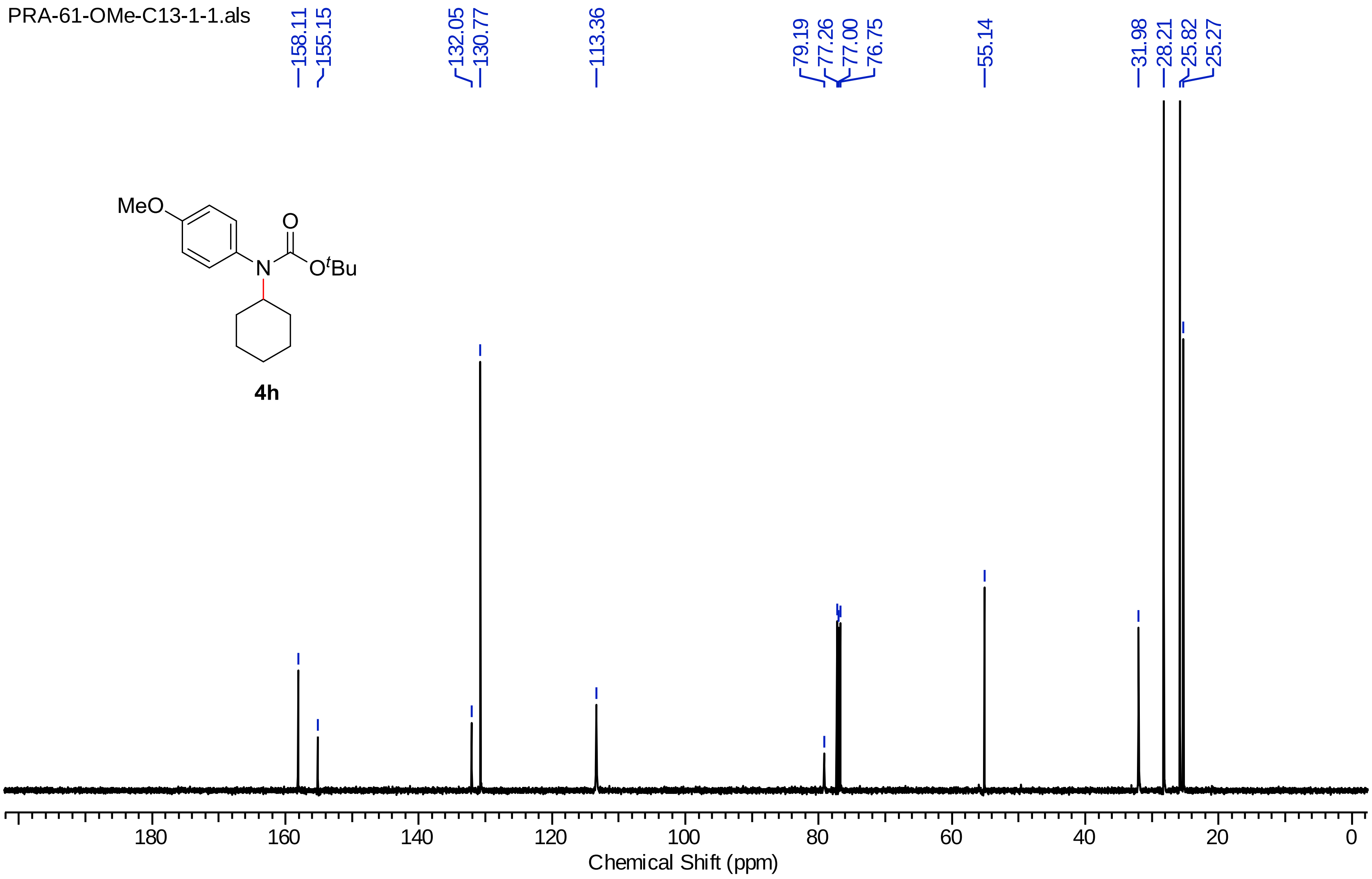
PRA-61-OMe-C13-1-1.als
158.11
155.15
132.05
130.77
113.36
79.19
77.26
77.00
76.75
55.14
31.98
28.21
25.82
25.27
MeO
O
N
O^{t}Bu
4h
180
160
140
120
100
80
60
40
20
0
Chemical Shift (ppm)

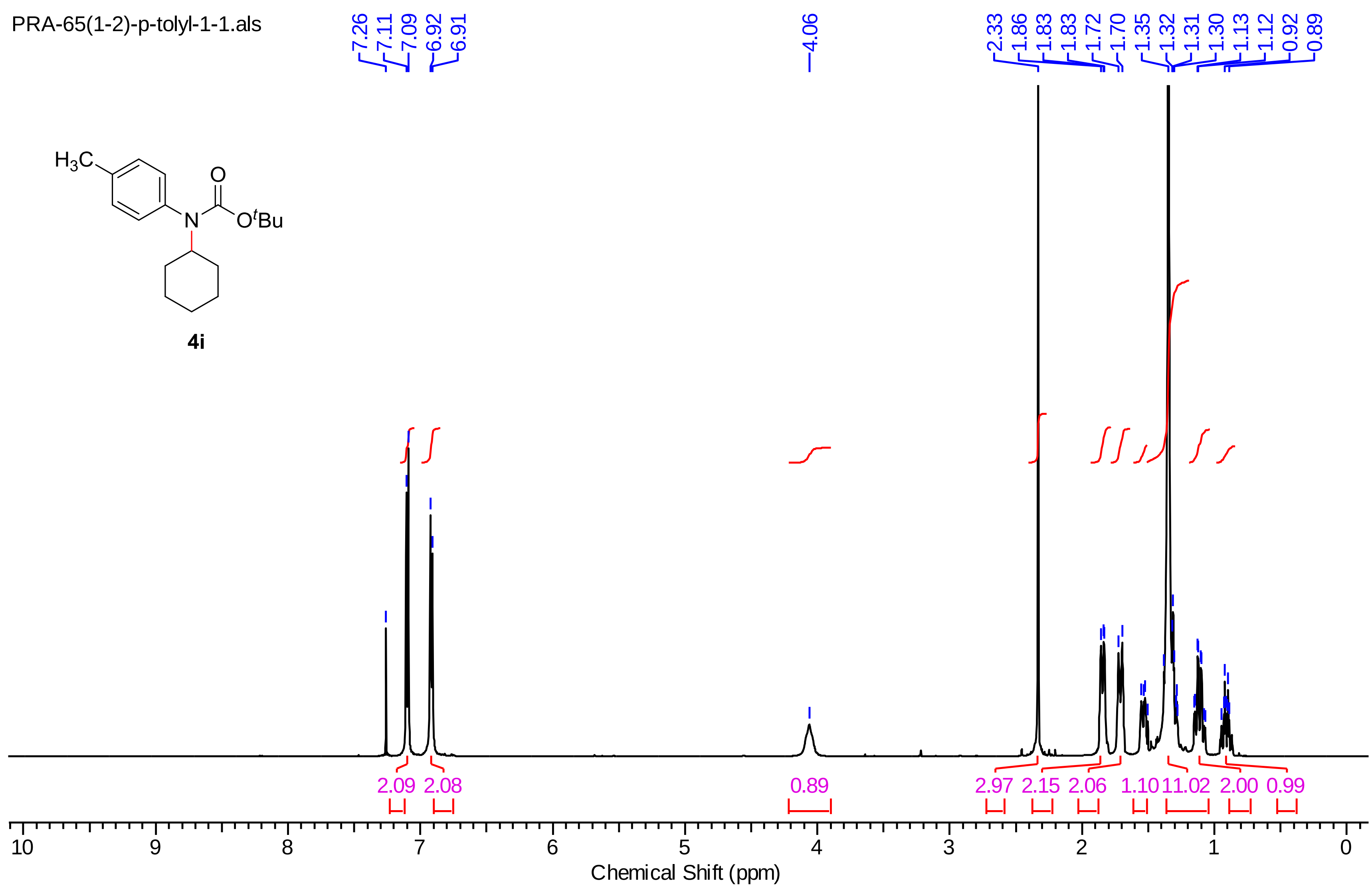
PRA-65(1-2)-p-tolyl-1-1.als
H3C
O
N
OtBu
4i
7.26
7.11
7.09
6.92
6.91
4.06
2.33
1.86
1.83
1.83
1.72
1.70
1.35
1.32
1.31
1.30
1.13
1.12
0.92
0.89
2.09
2.08
0.89
2.97
2.15
2.06
1.10
11.02
2.00
0.99
10
9
8
7
6
5
4
3
2
1
0
Chemical Shift (ppm)

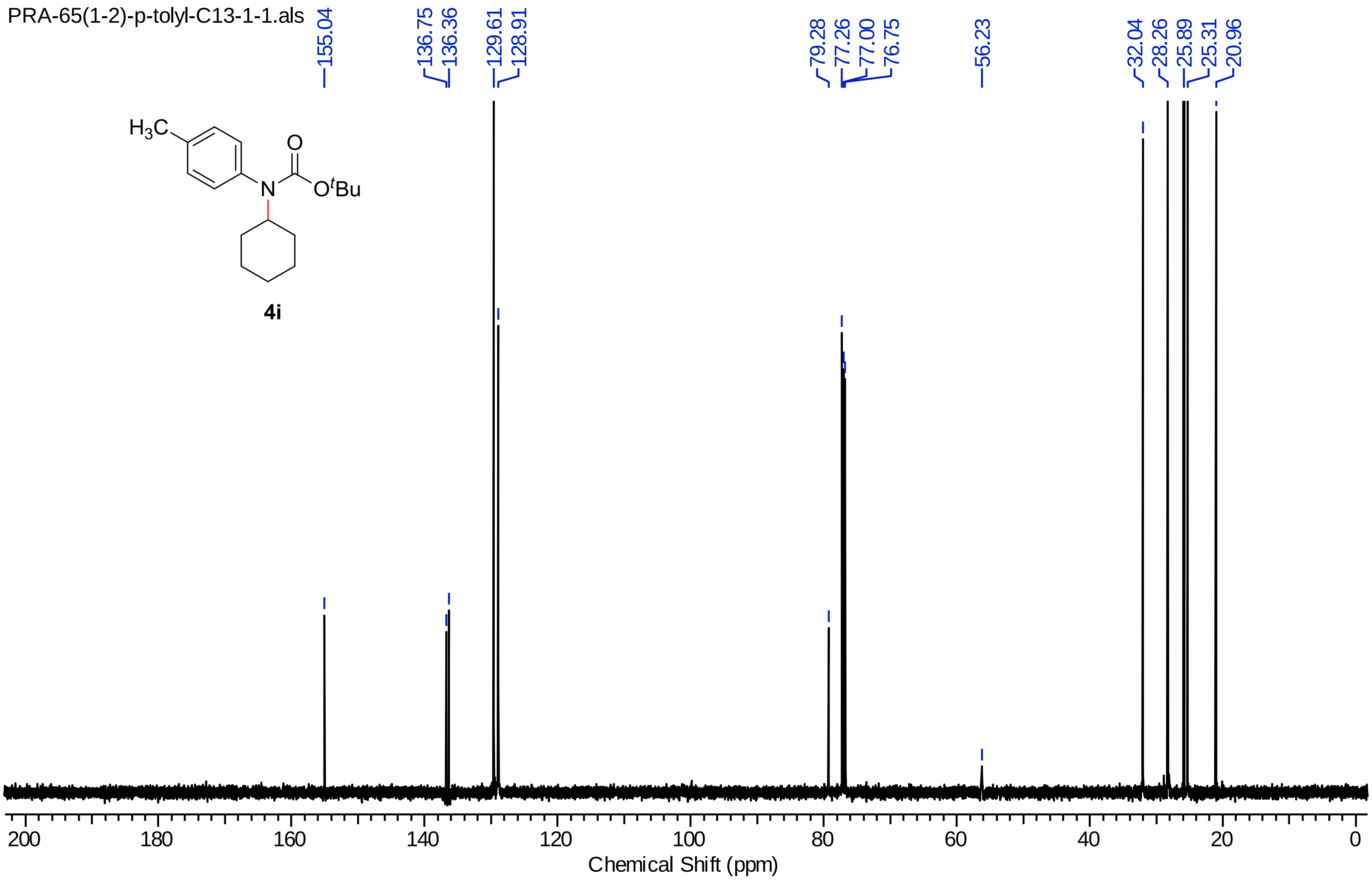
PRA-65(1-2)-p-tolyl-C13-1-1.als
155.04
136.75
136.36
129.61
128.91
79.28
77.26
77.00
76.75
56.23
32.04
28.26
25.89
25.31
20.96
H3C
O
N
OtBu
4i
200
180
160
140
120
100
80
60
40
20
0
Chemical Shift (ppm)

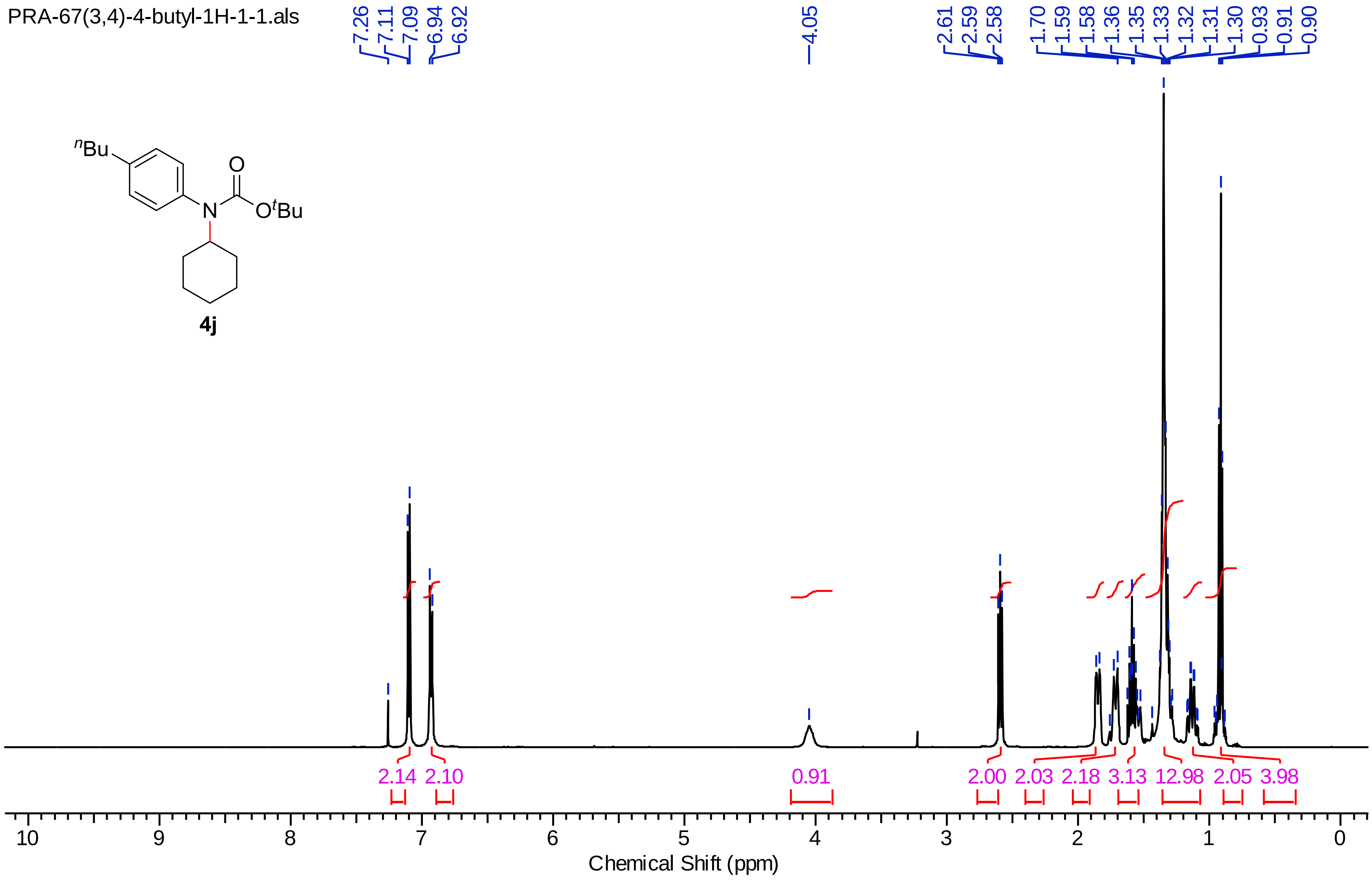

PRA-67(3,4)-4-butyl-1H-1-1.als
7.26
7.11
7.09
6.94
6.92
4.05
2.61
2.59
2.58
1.70
1.59
1.58
1.36
1.35
1.33
1.32
1.31
1.30
0.93
0.91
0.90
nBu
O
N
OtBu
4j
2.14
2.10
0.91
2.00
2.03
2.18
3.13
12.98
2.05
3.98
10
9
8
7
6
5
4
3
2
1
0
Chemical Shift (ppm)

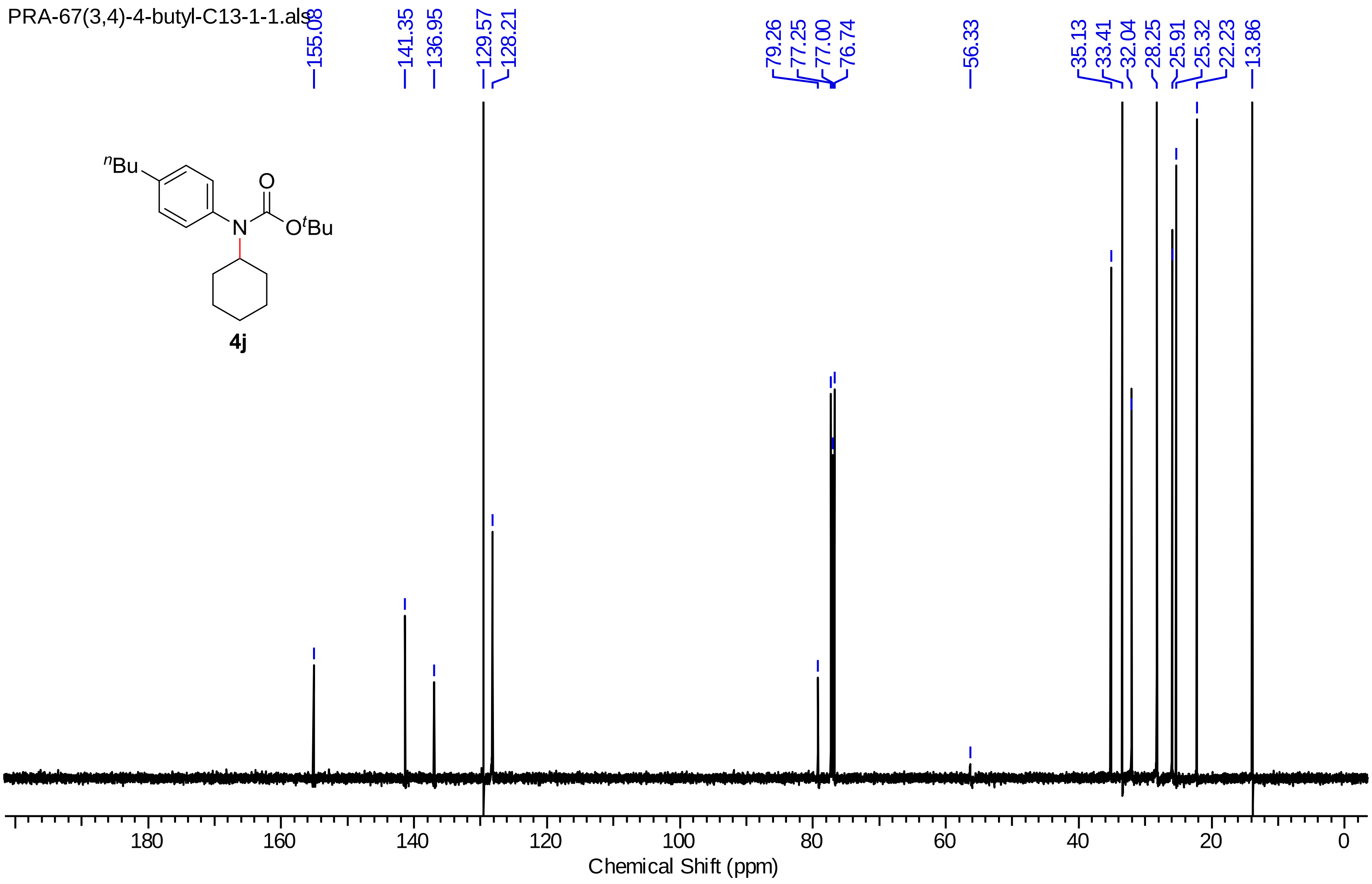
PRA-67(3,4)-4-butyl-C13-1-1.als
155.08
141.35
136.95
129.57
128.21
79.26
77.25
77.00
76.74
56.33
35.13
33.41
32.04
28.25
25.91
25.32
22.23
13.86
180
160
140
120
100
80
60
40
20
0
Chemical Shift (ppm)

4j

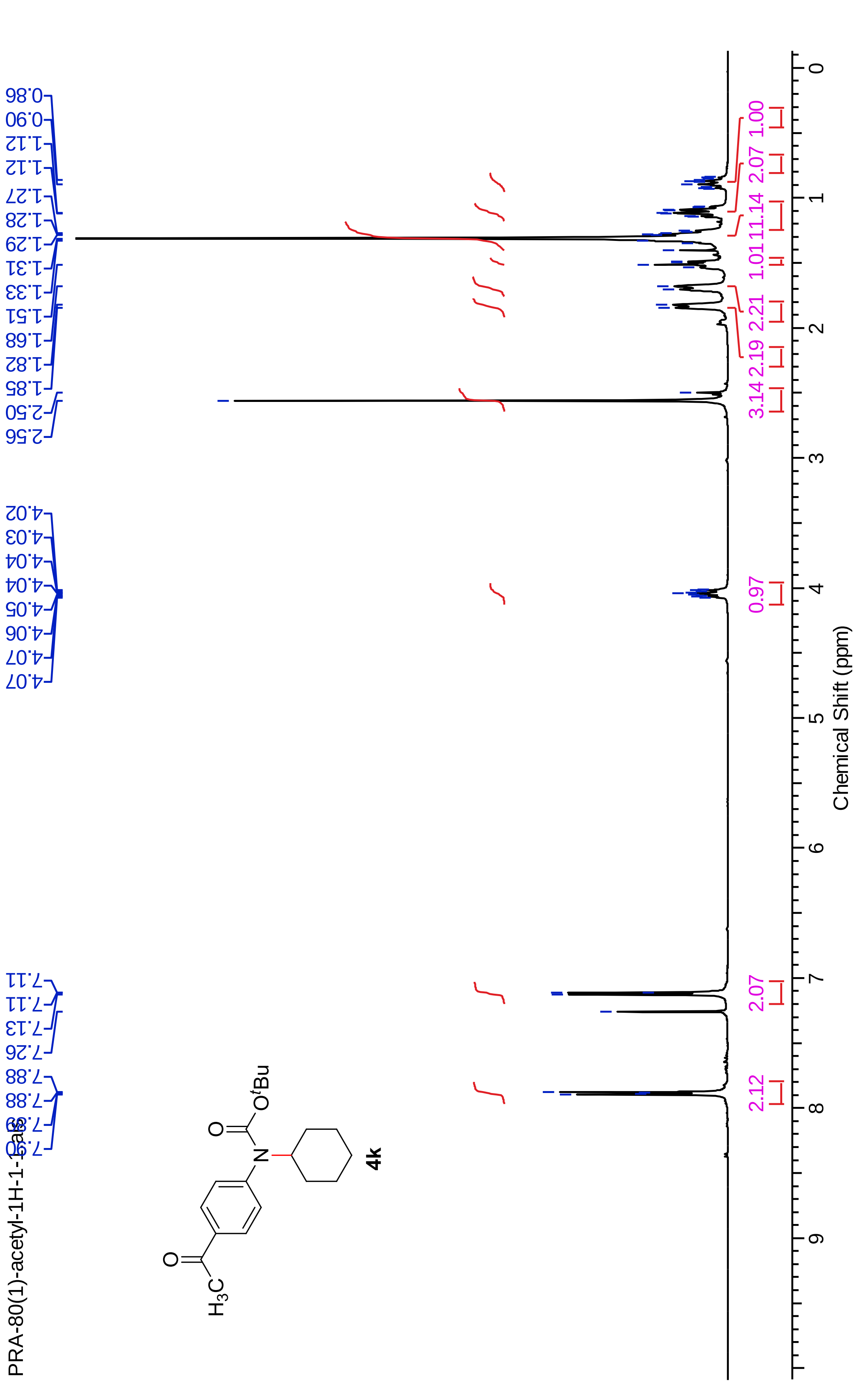
PRA-80(1)-acetyl-1H-1-1.als
4k
7.90
7.89
7.88
7.88
7.26
7.13
7.11
7.11
4.07
4.07
4.06
4.05
4.04
4.04
4.03
4.02
2.56
2.50
1.85
1.82
1.68
1.51
1.33
1.31
1.29
1.28
1.27
1.12
1.12
0.90
0.86
2.12
2.07
0.97
3.14
2.19
2.21
1.01
11.14
2.07
1.00
Chemical Shift (ppm)

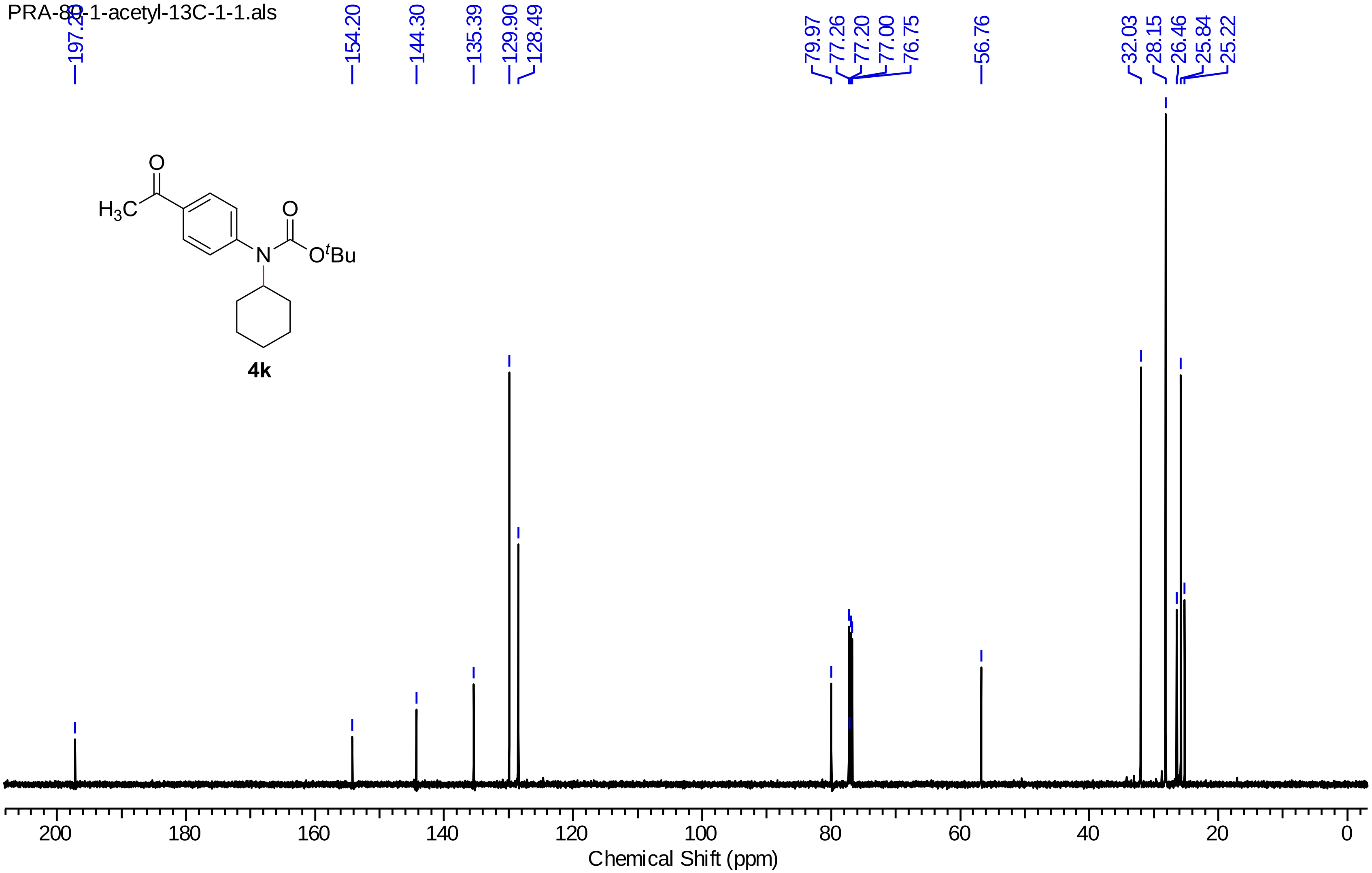
PRA-80-1-acetyl-13C-1-1.als
197.20
154.20
144.30
135.39
129.90
128.49
79.97
77.26
77.20
77.00
76.75
56.76
32.03
28.15
26.46
25.84
25.22
H3C
O
N
O
OtBu
4k
200 180 160 140 120 100 80 60 40 20 0
Chemical Shift (ppm)

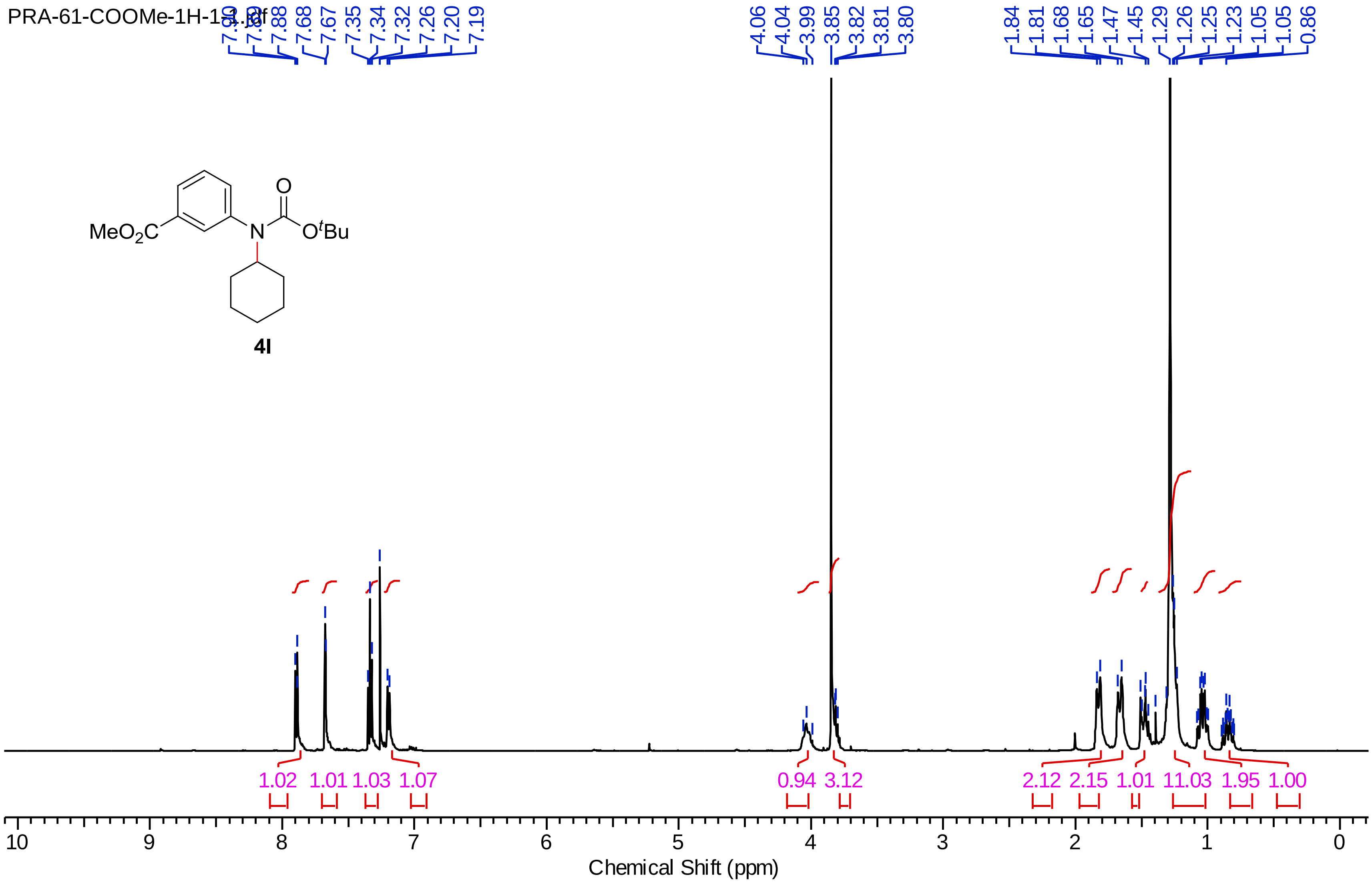
PRA-61-COOMe-1H-1-1.jdf
7.90
7.89
7.88
7.68
7.67
7.35
7.34
7.32
7.26
7.20
7.19
4.06
4.04
3.99
3.85
3.82
3.81
3.80
1.84
1.81
1.68
1.65
1.47
1.45
1.29
1.26
1.25
1.23
1.05
1.05
0.86
MeO2C
N
O
OtBu
4l
1.02 1.01 1.03 1.07
0.94 3.12
2.12 2.15 1.01 11.03 1.95 1.00
10 9 8 7 6 5 4 3 2 1 0
Chemical Shift (ppm)

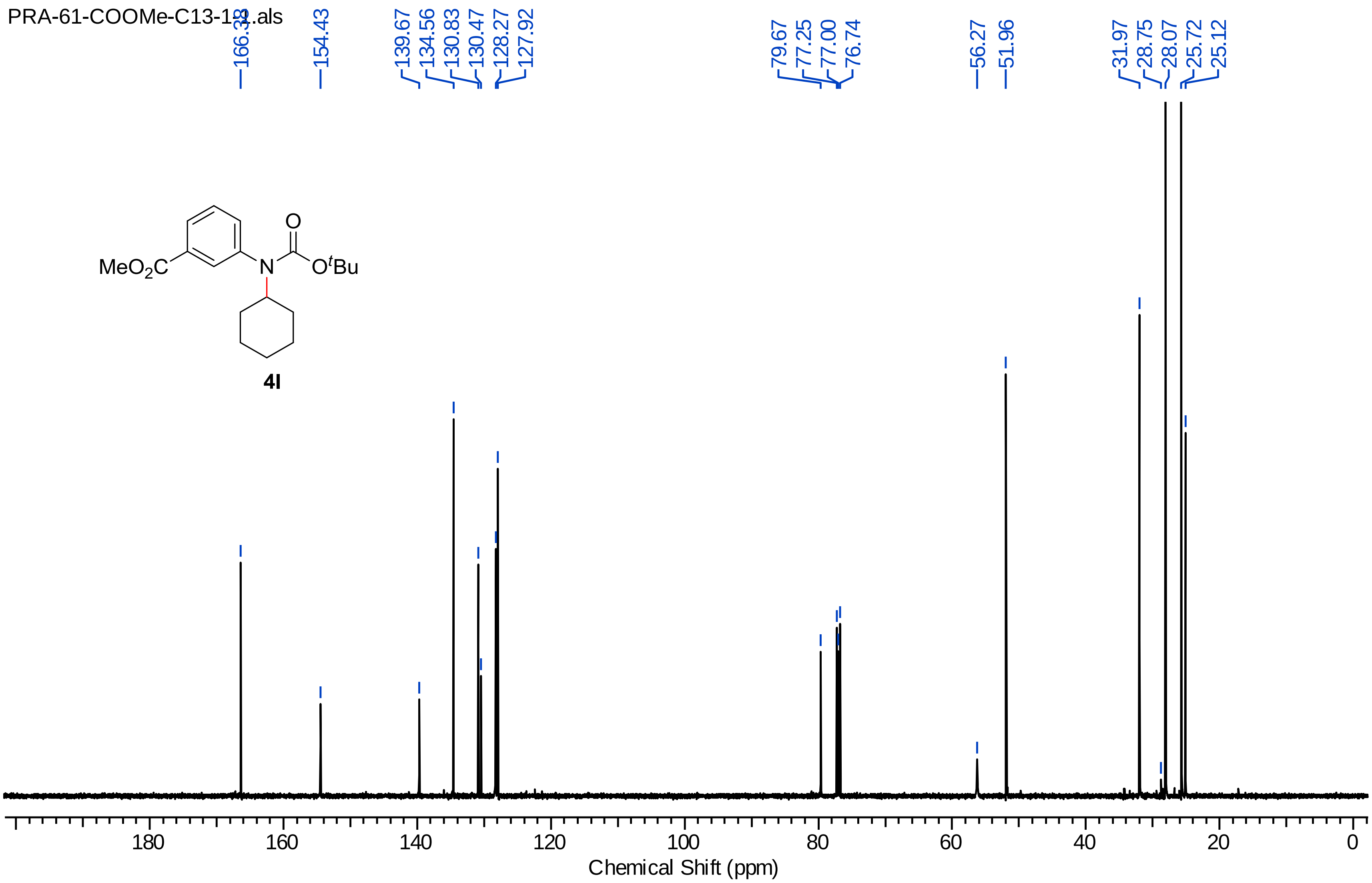

154.43
139.67
134.56
130.83
130.47
128.27
127.92
79.67
77.25
77.00
76.74
56.27
51.96
31.97
28.75
28.07
25.72
25.12
MeO2C
N
O
OtBu
180
160
140
120
100
80
60
40
20
0
Chemical Shift (ppm)

4l

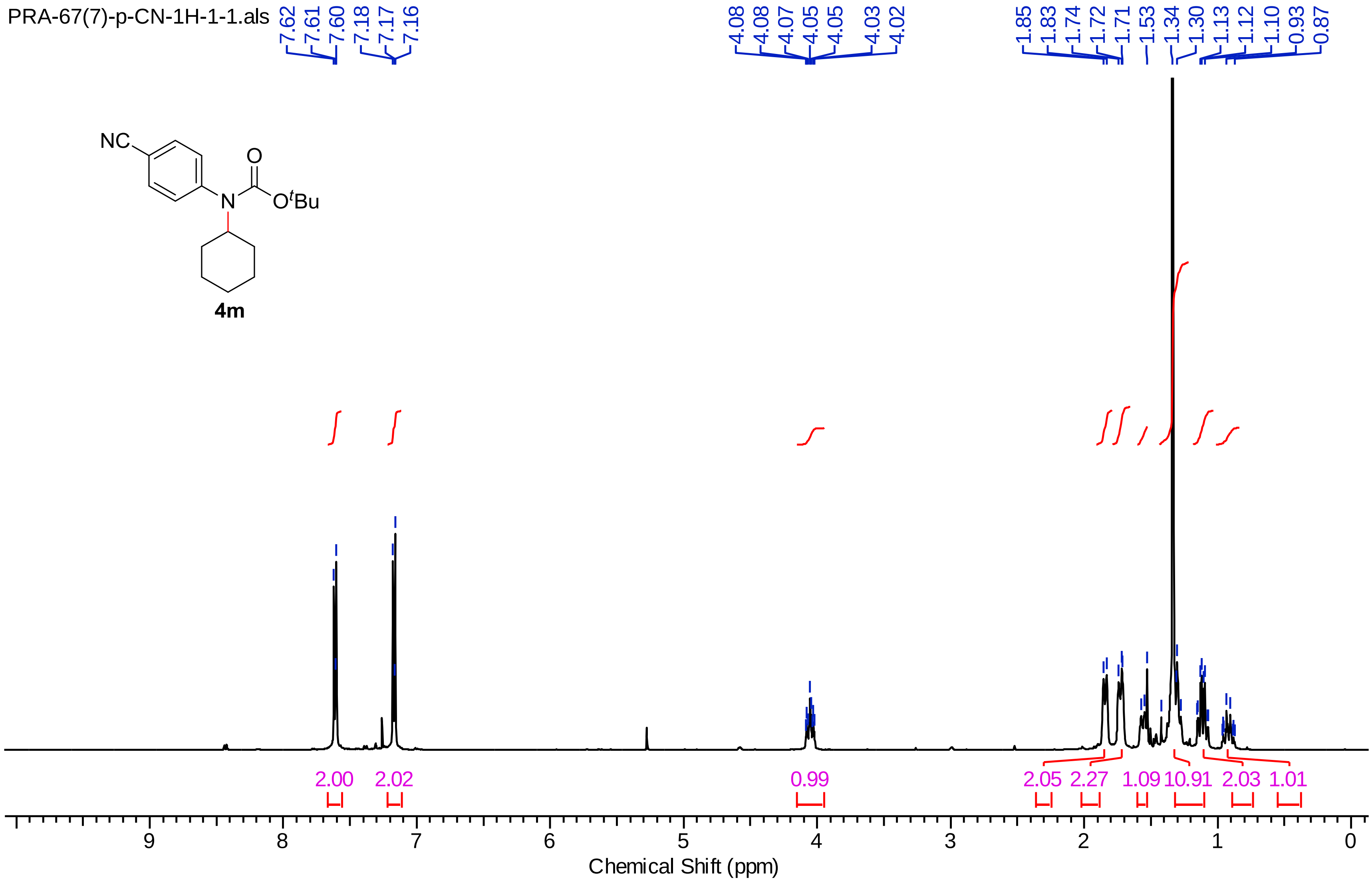
PRA-67(7)-p-CN-1H-1-1.als
7.62
7.61
7.60
7.18
7.17
7.16
4.08
4.08
4.07
4.05
4.05
4.03
4.02
1.85
1.83
1.74
1.72
1.71
1.53
1.34
1.30
1.13
1.12
1.10
0.93
0.87
NC
O
N
O^{t}Bu
4m
2.00
2.02
0.99
2.05
2.27
1.09
10.91
2.03
1.01
9
8
7
6
5
4
3
2
1
0
Chemical Shift (ppm)

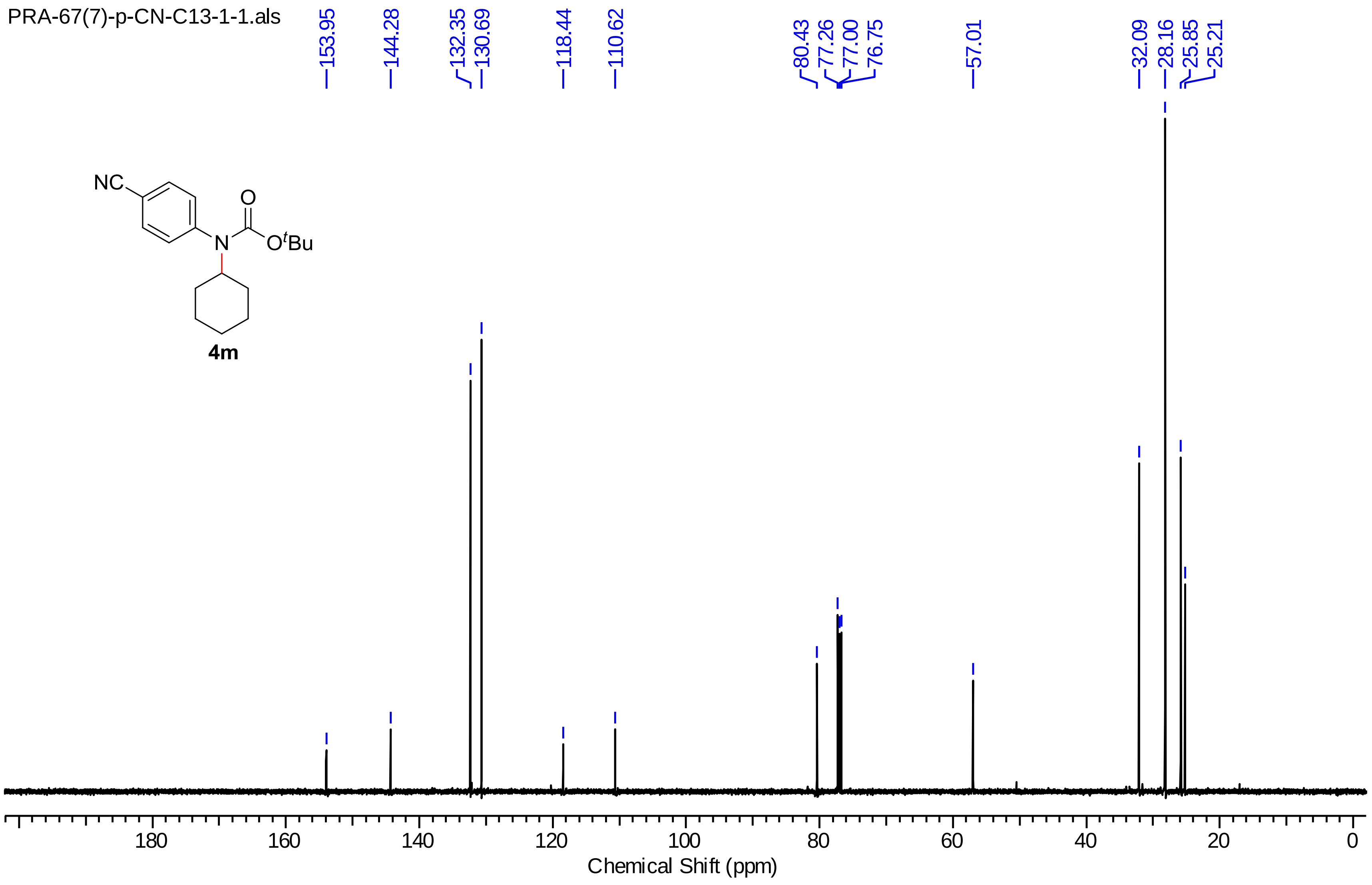
PRA-67(7)-p-CN-C13-1-1.als
153.95
144.28
132.35
130.69
118.44
110.62
80.43
77.26
77.00
76.75
57.01
32.09
28.16
25.85
25.21
NC
O
N
O^{t}Bu
4m
180
160
140
120
100
80
60
40
20
0
Chemical Shift (ppm)

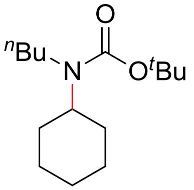

4n

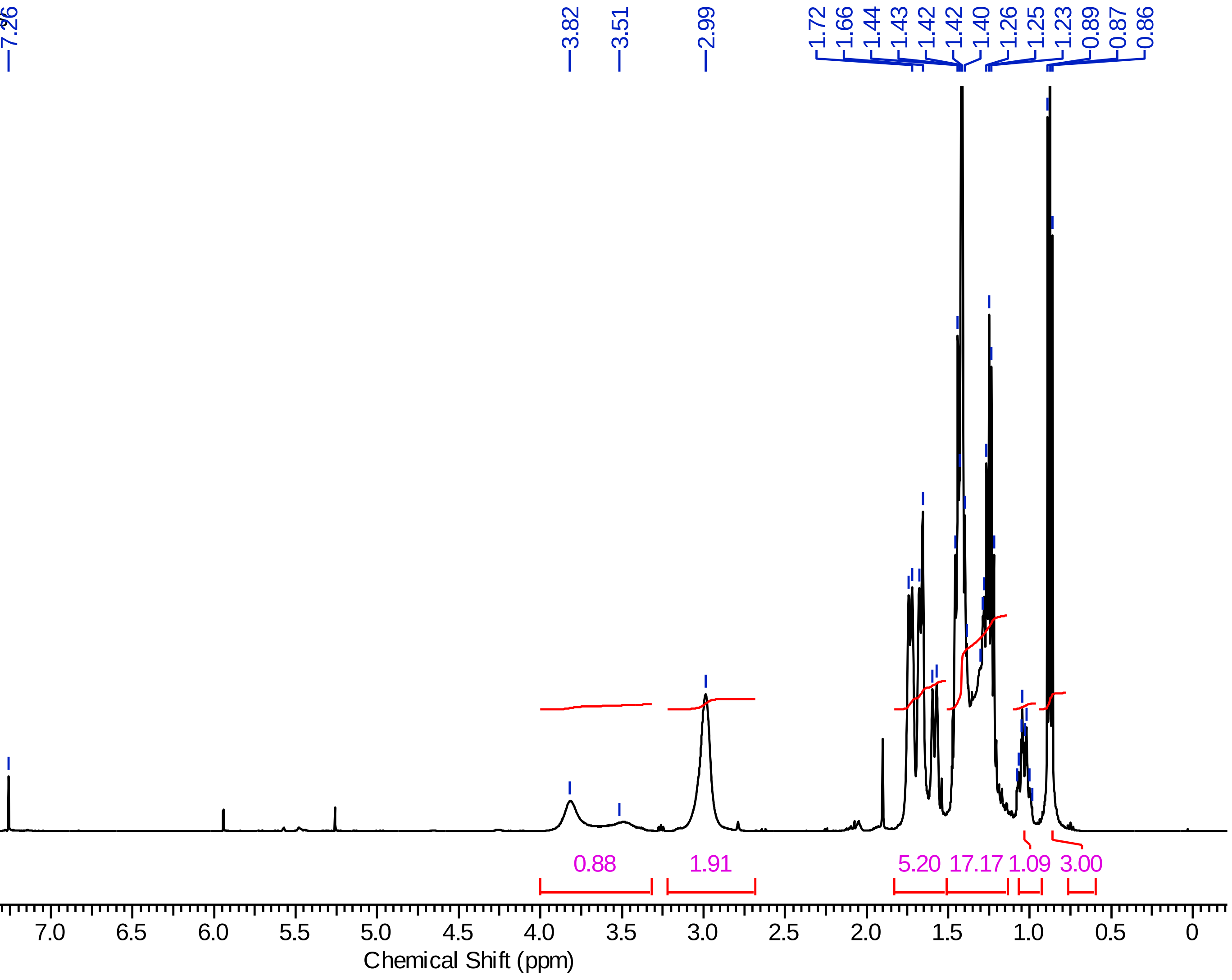
PRA-67(1,2)-n-Butyl-1-1.als
7.26
3.82
3.51
2.99
1.72
1.66
1.44
1.43
1.42
1.42
1.40
1.26
1.25
1.23
0.89
0.87
0.86
0.88
1.91
5.20
17.17
1.09
3.00
8.5
8.0
7.5
7.0
6.5
6.0
5.5
5.0
4.5
4.0
3.5
3.0
2.5
2.0
1.5
1.0
0.5
0
Chemical Shift (ppm)

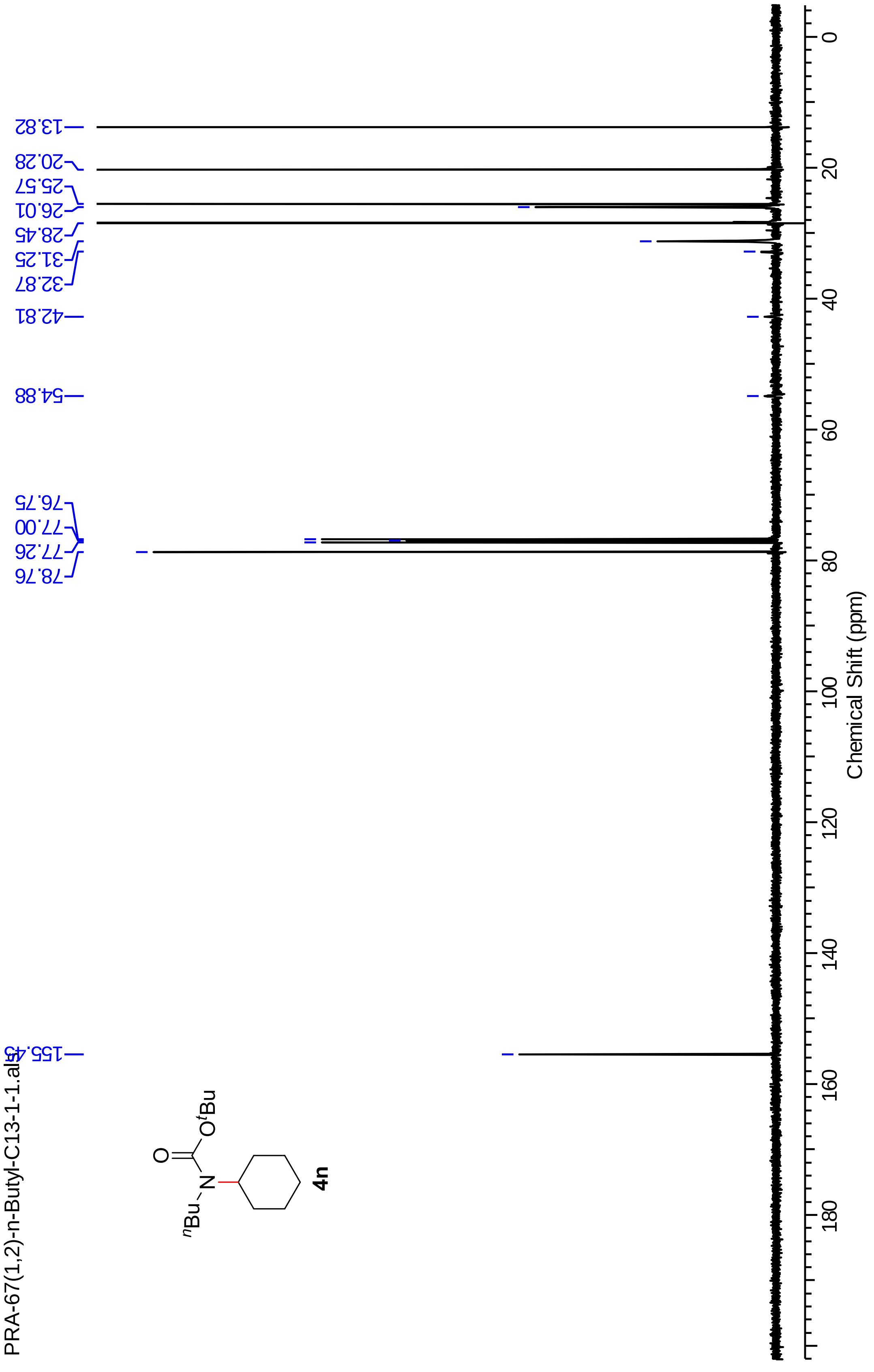
PRA-67(1,2)-n-Butyl-C13-1-1.als
155.45
78.76
77.26
77.00
76.75
54.88
42.81
32.87
31.25
28.45
26.01
25.57
20.28
13.82
O
O^{t}Bu
^{n}Bu
N
4n
Chemical Shift (ppm)
0
20
40
60
80
100
120
140
160
180

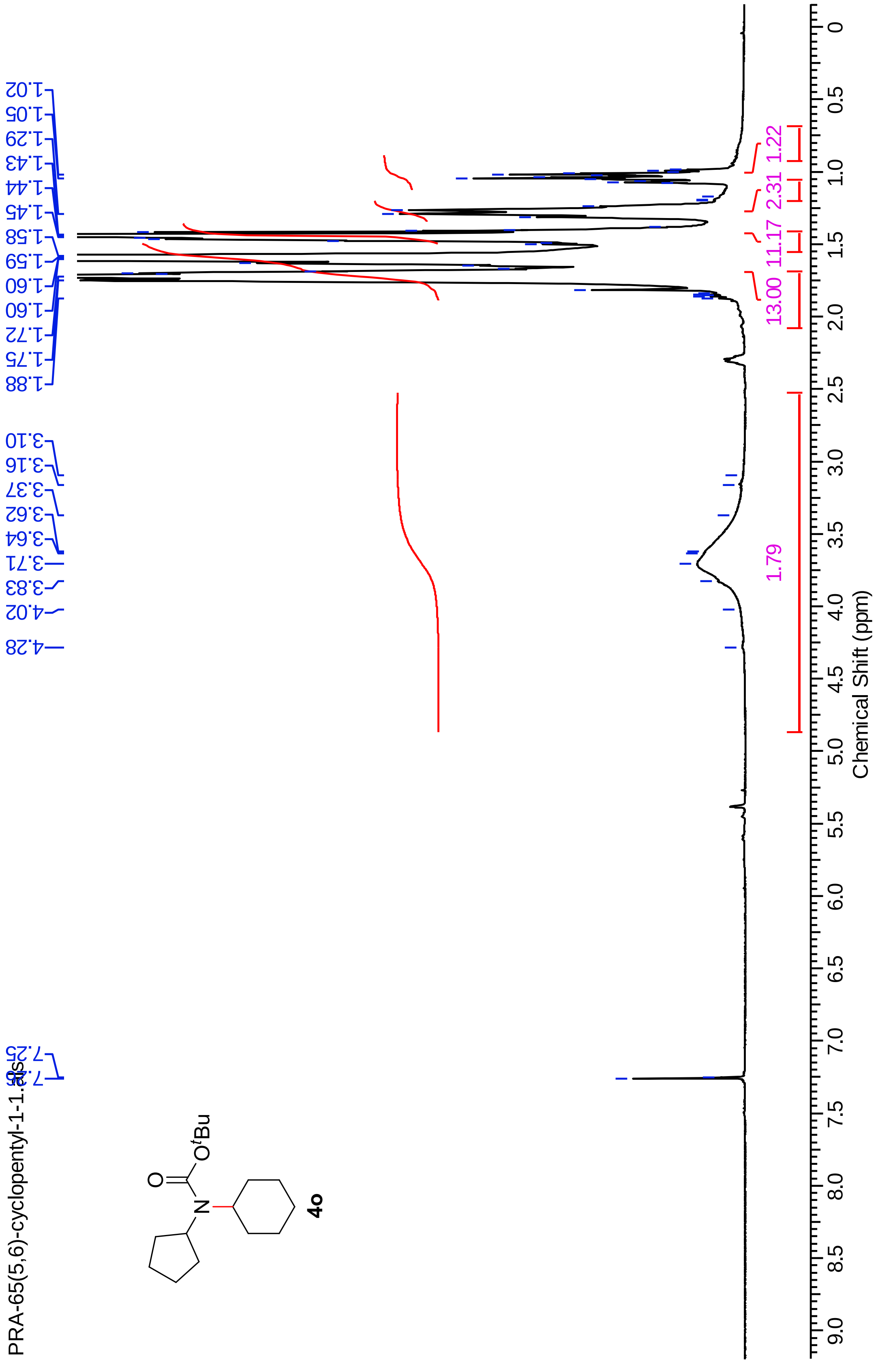
PRA-65(5,6)-cyclopentyl-1-1.fid
4o
7.25
7.26
4.28
4.02
3.83
3.71
3.64
3.62
3.37
3.16
3.10
1.88
1.75
1.72
1.60
1.60
1.59
1.58
1.45
1.44
1.43
1.29
1.05
1.02
1.79
13.00
11.17
2.31
1.22
Chemical Shift (ppm)

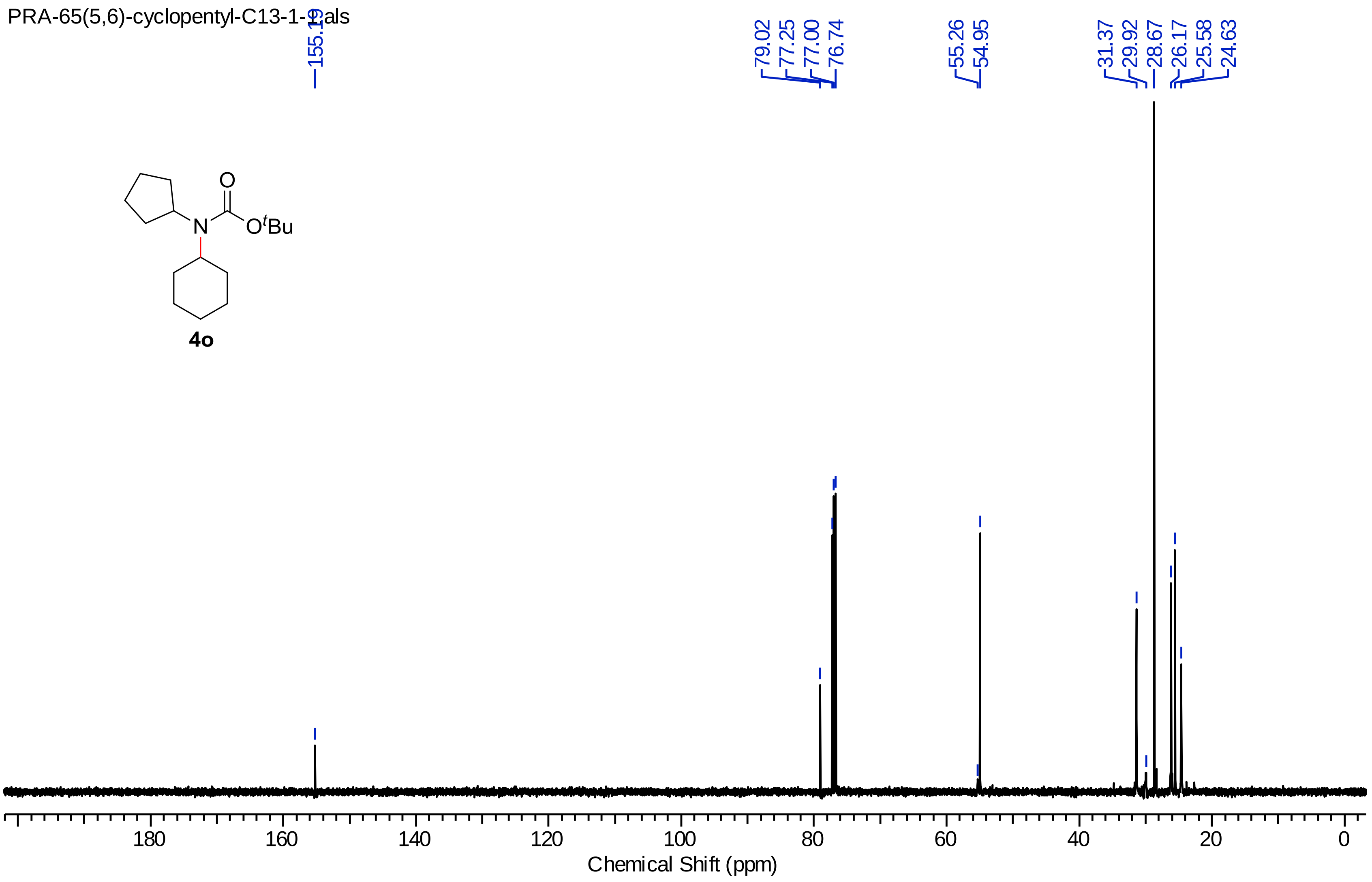
PRA-65(5,6)-cyclopentyl-C13-1-1.als
155.19
79.02
77.25
77.00
76.74
55.26
54.95
31.37
29.92
28.67
26.17
25.58
24.63
O
N
O^{t}Bu
4o
180
160
140
120
100
80
60
40
20
0
Chemical Shift (ppm)

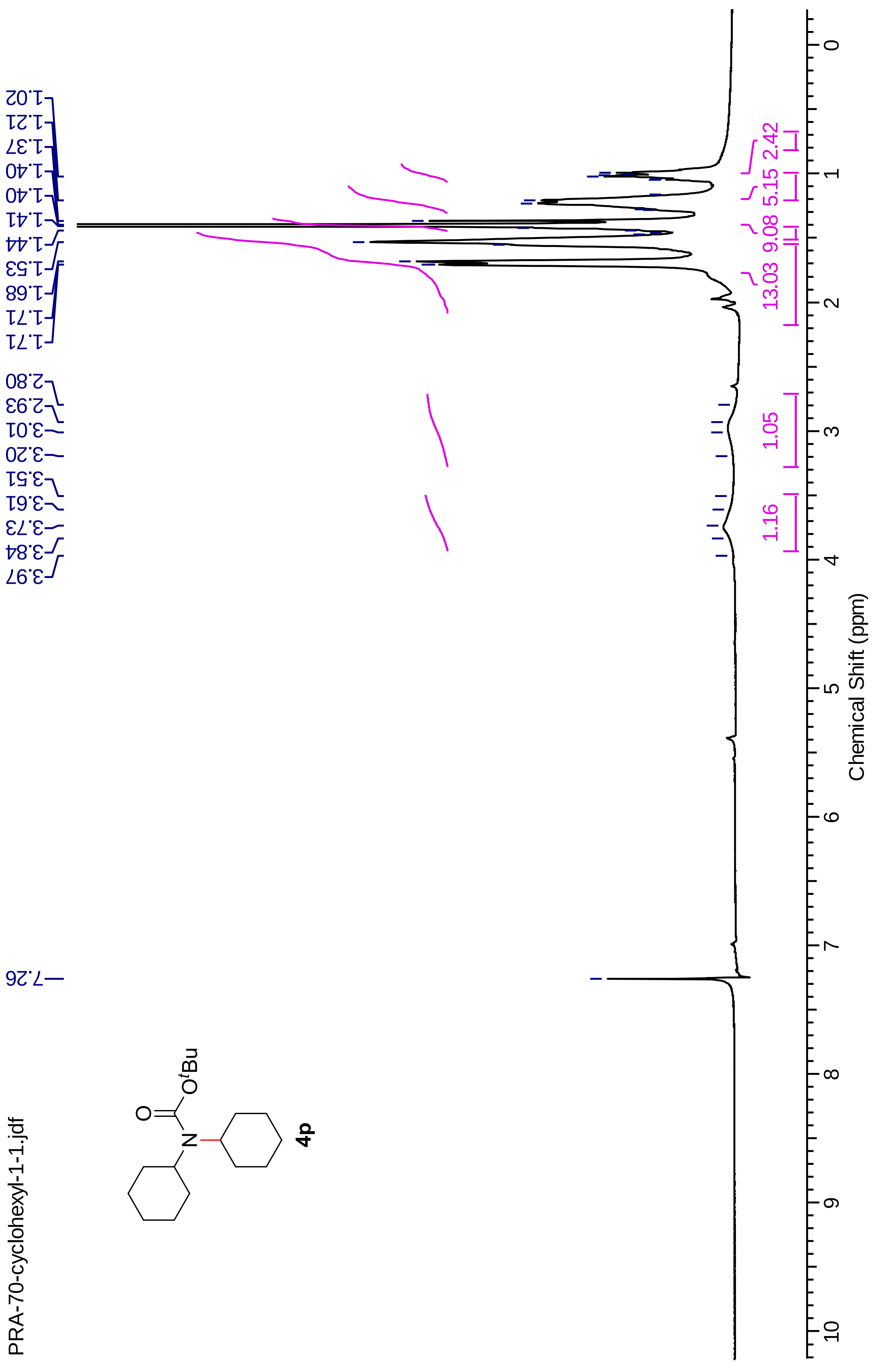
PRA-70-cyclohexyl-1-1.jdf
O
N
O^{t}Bu
4p
7.26
3.97
3.84
3.73
3.61
3.51
3.20
3.01
2.93
2.80
1.71
1.71
1.68
1.53
1.44
1.41
1.40
1.40
1.37
1.21
1.02
1.16
1.05
13.03
9.08
5.15
2.42
Chemical Shift (ppm)

PRA-70-cyclohexyl-C13-1-1.jdf

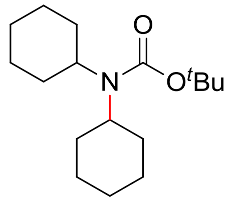

4p

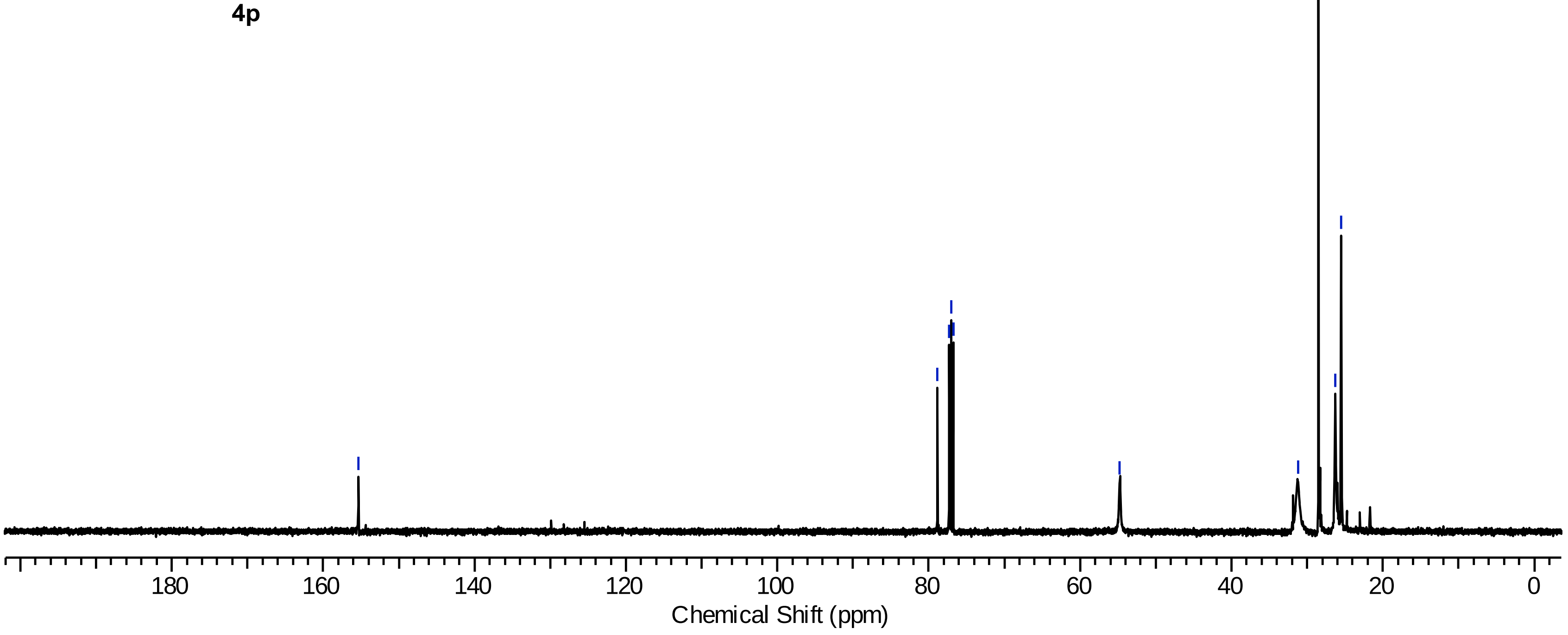

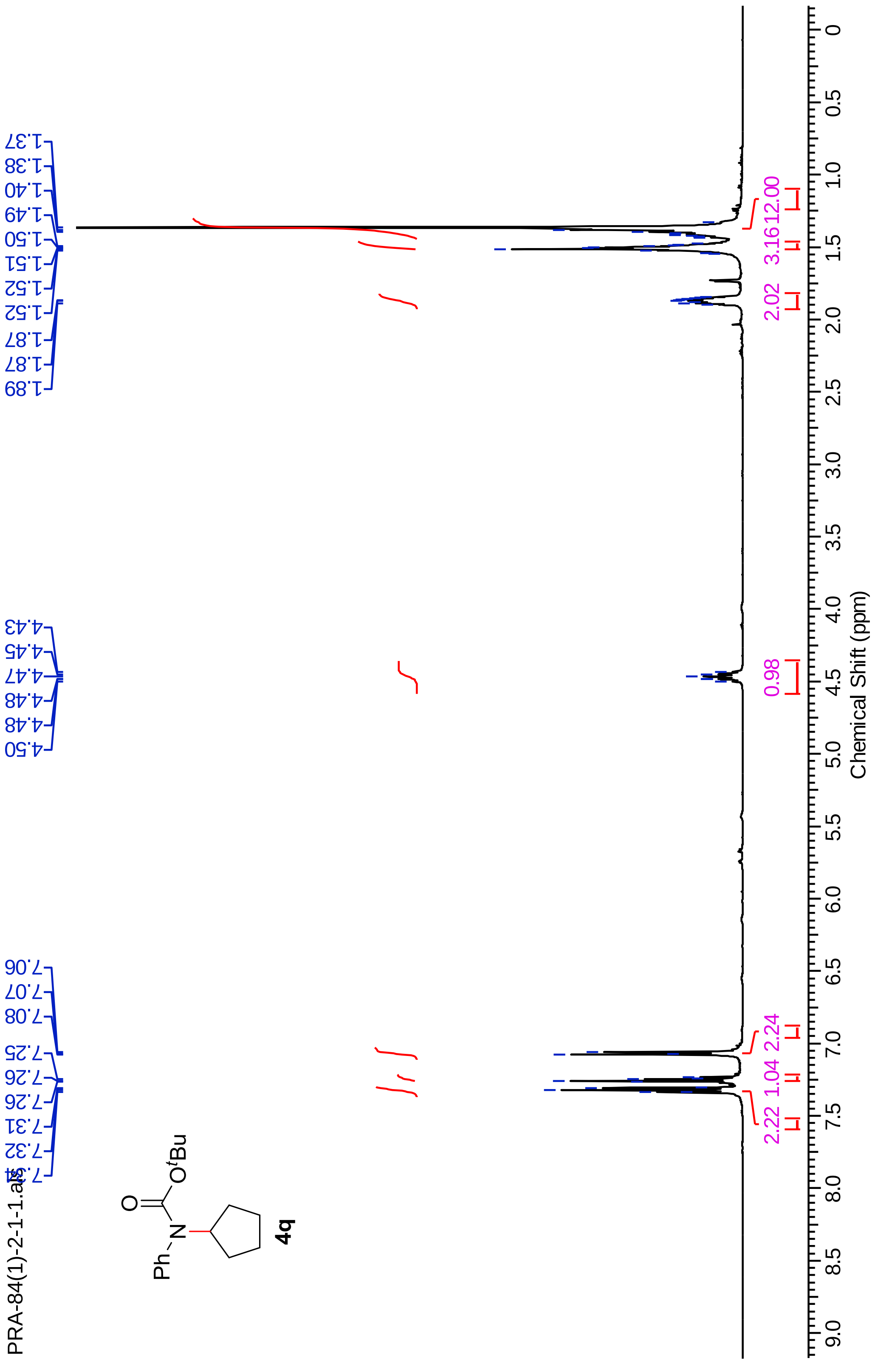

PRA-84(1)-2-1-1.als
4q
Ph
N
O
O^tBu
7.34
7.32
7.31
7.26
7.26
7.25
7.08
7.07
7.06
4.50
4.48
4.48
4.47
4.45
4.43
1.89
1.87
1.87
1.52
1.52
1.51
1.50
1.49
1.40
1.38
1.37
2.22
1.04
2.24
0.98
2.02
3.16
12.00
Chemical Shift (ppm)

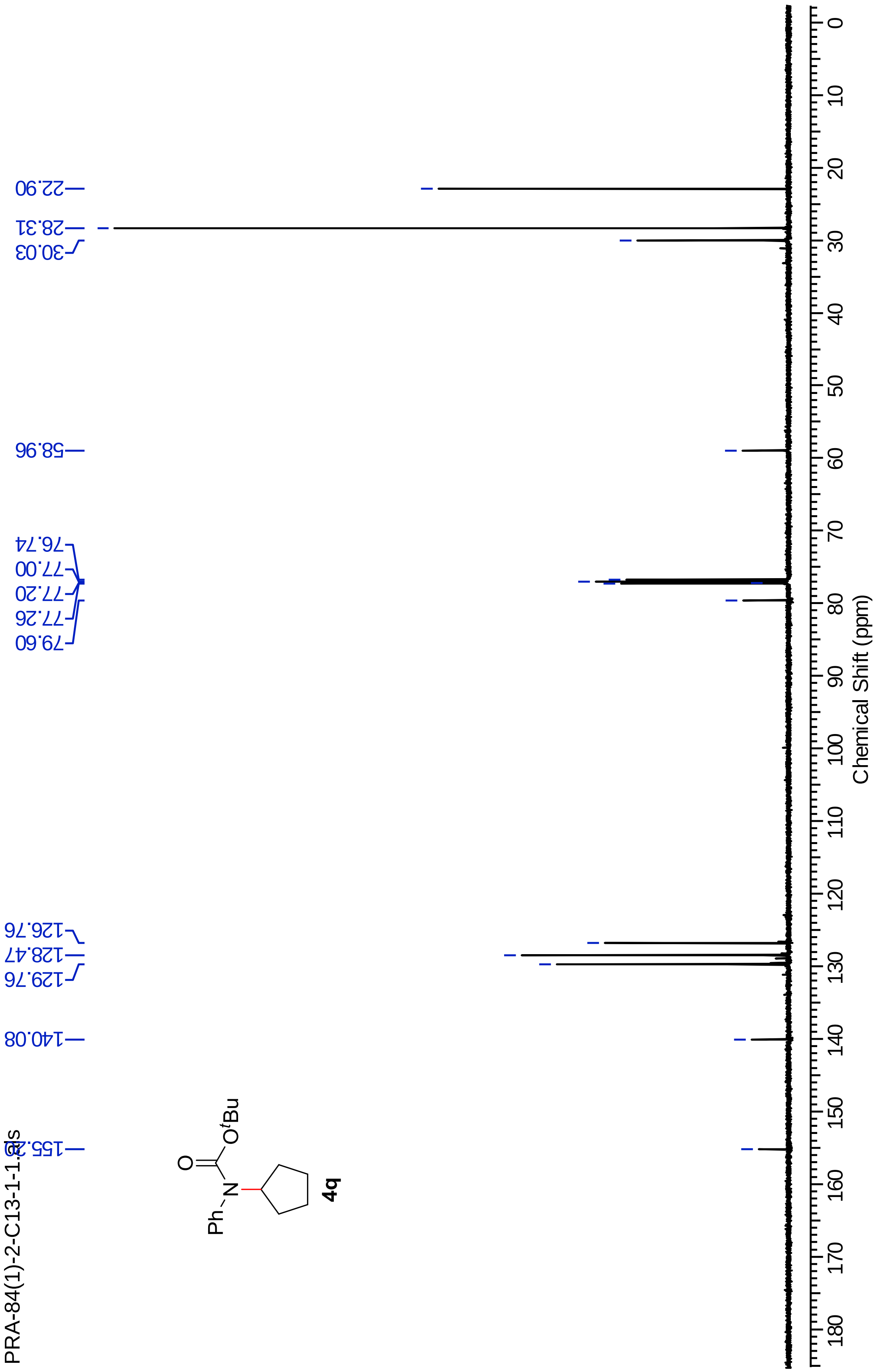
PRA-84(1)-2-C13-1-1
22.90
28.31
30.03
58.96
76.74
77.00
77.20
77.26
79.60
126.76
128.47
129.76
140.08
155.2
Chemical Shift (ppm)
4q

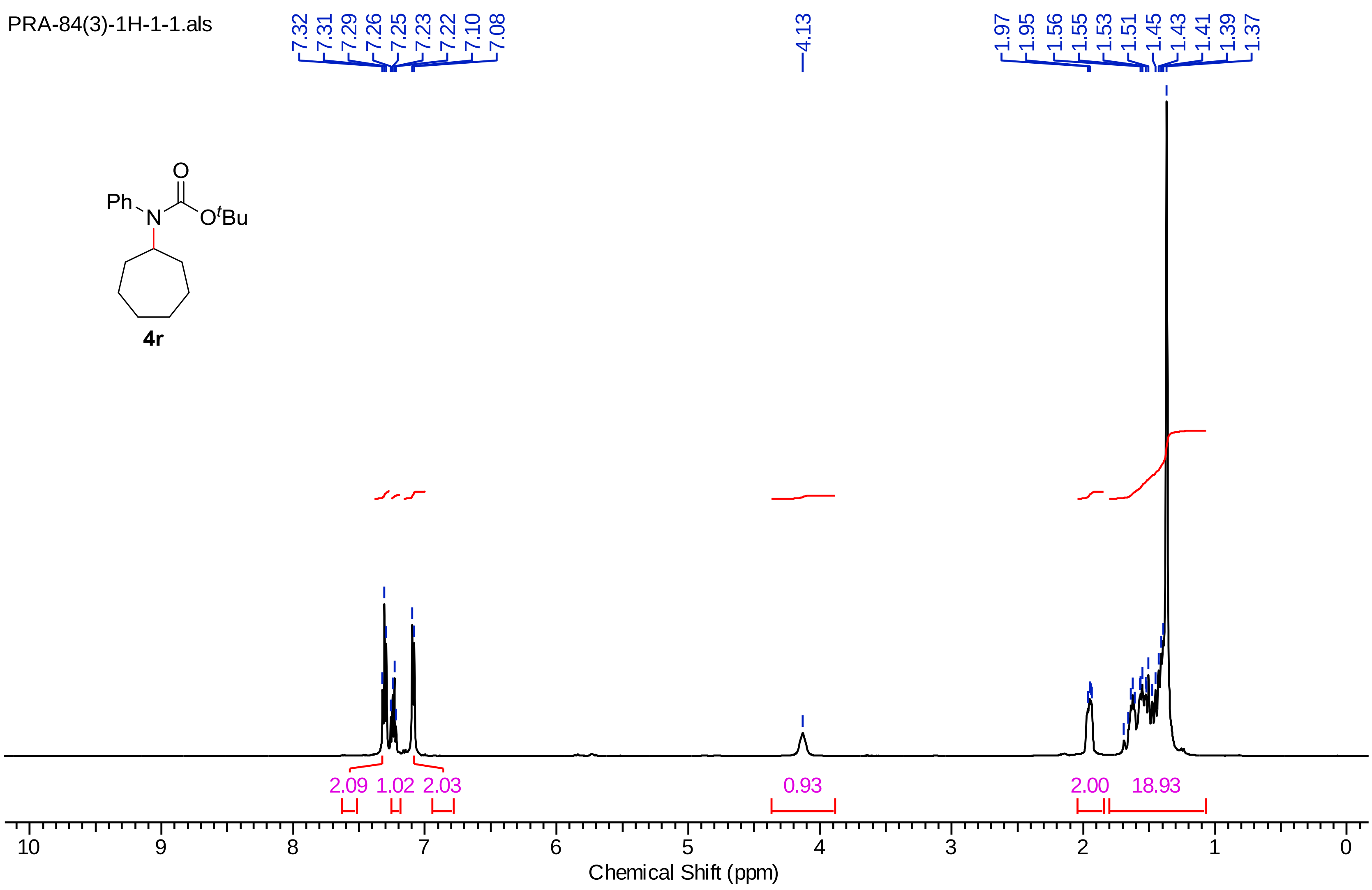
PRA-84(3)-1H-1-1.als
Ph
N
O
O^{t}Bu
4r
7.32
7.31
7.29
7.26
7.25
7.23
7.22
7.10
7.08
4.13
1.97
1.95
1.56
1.55
1.53
1.51
1.45
1.43
1.41
1.39
1.37
2.09
1.02
2.03
0.93
2.00
18.93
10
9
8
7
6
5
4
3
2
1
0
Chemical Shift (ppm)

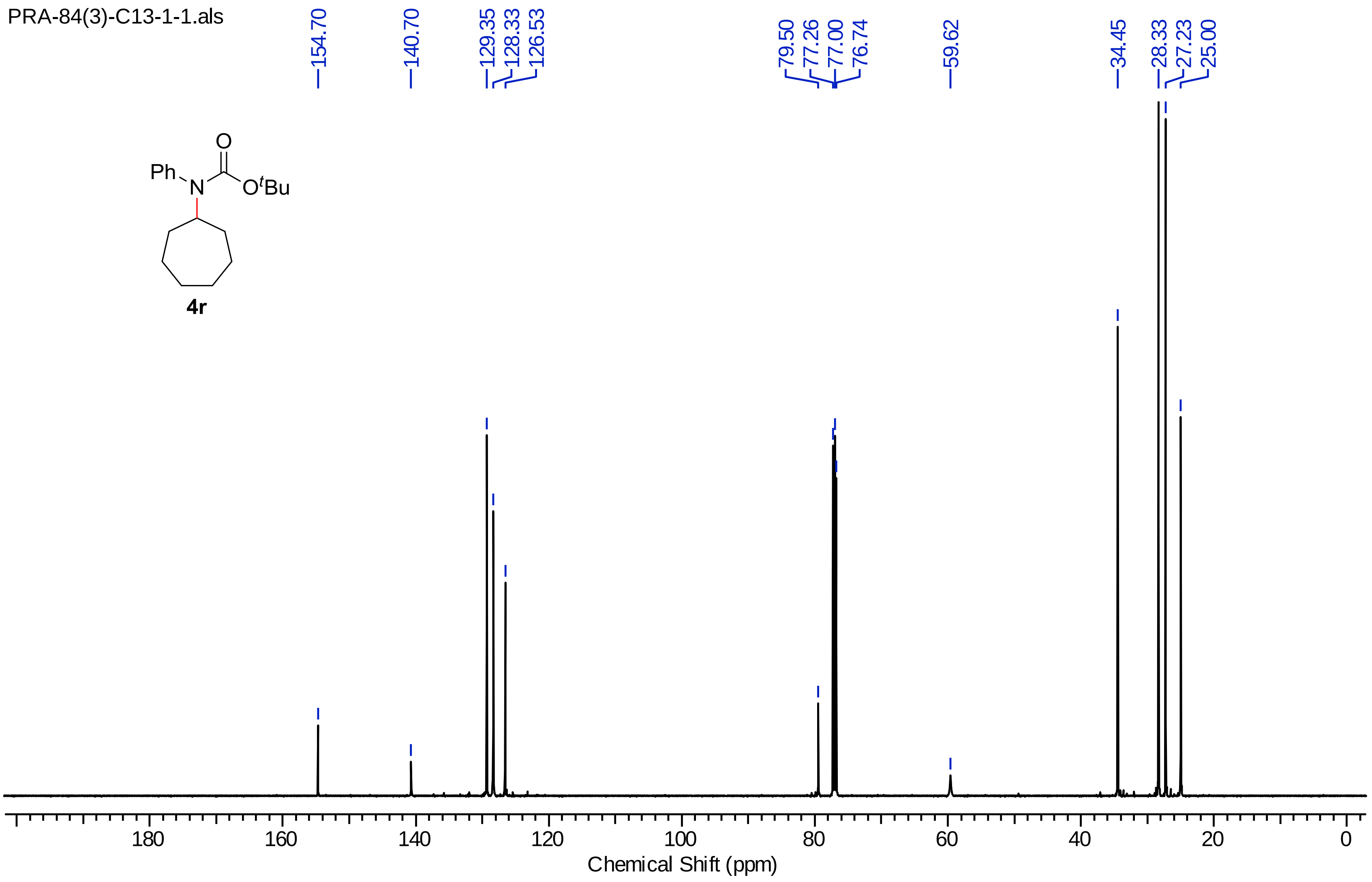

PRA-84(3)-C13-1-1.als
154.70
140.70
129.35
128.33
126.53
79.50
77.26
77.00
76.74
59.62
34.45
28.33
27.23
25.00
Ph
N
O
O^{t}Bu
4r
180
160
140
120
100
80
60
40
20
0
Chemical Shift (ppm)

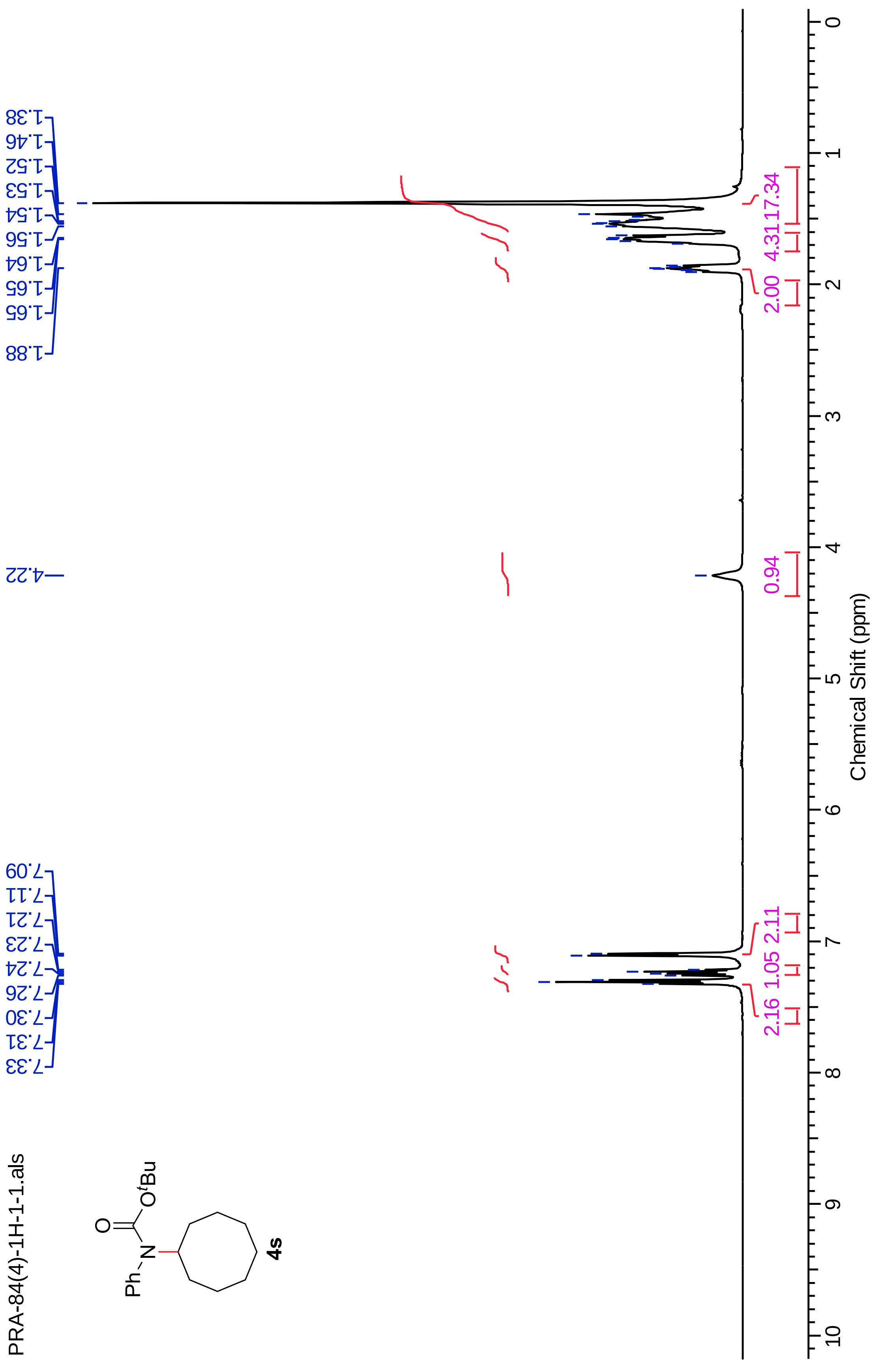

PRA-84(4)-1H-1-1.als
Ph
N
O
O^tBu
4s
7.33
7.31
7.30
7.26
7.24
7.23
7.21
7.11
7.09
4.22
1.88
1.65
1.65
1.64
1.56
1.54
1.53
1.52
1.46
1.38
2.16
1.05
2.11
0.94
2.00
4.31
17.34
10
9
8
7
6
5
4
3
2
1
0
Chemical Shift (ppm)

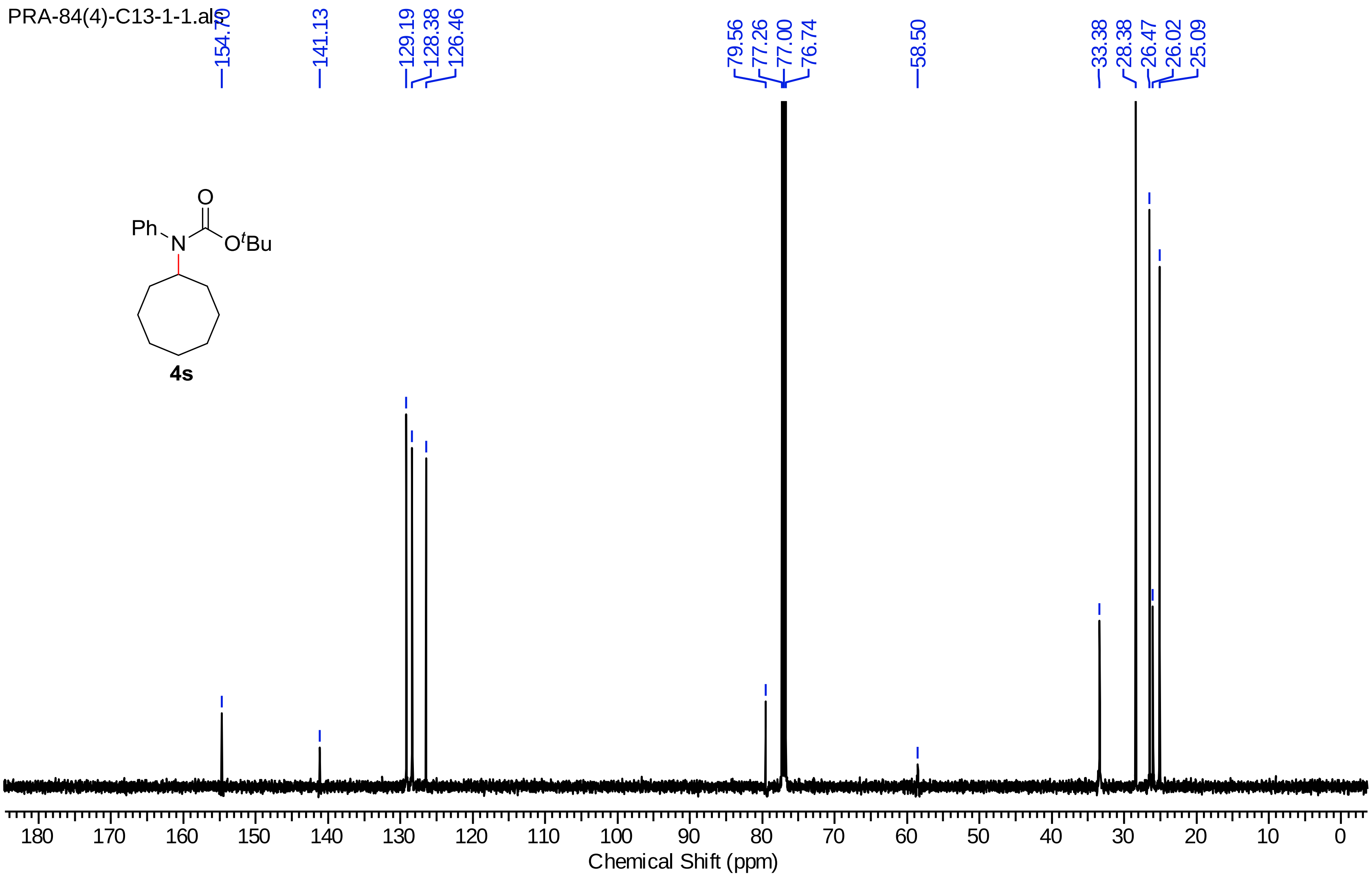
PRA-84(4)-C13-1-1.als
154.70
141.13
129.19
128.38
126.46
79.56
77.26
77.00
76.74
58.50
33.38
28.38
26.47
26.02
25.09
O
Ph
N
O^{t}Bu
4s
180 170 160 150 140 130 120 110 100 90 80 70 60 50 40 30 20 10 0
Chemical Shift (ppm)

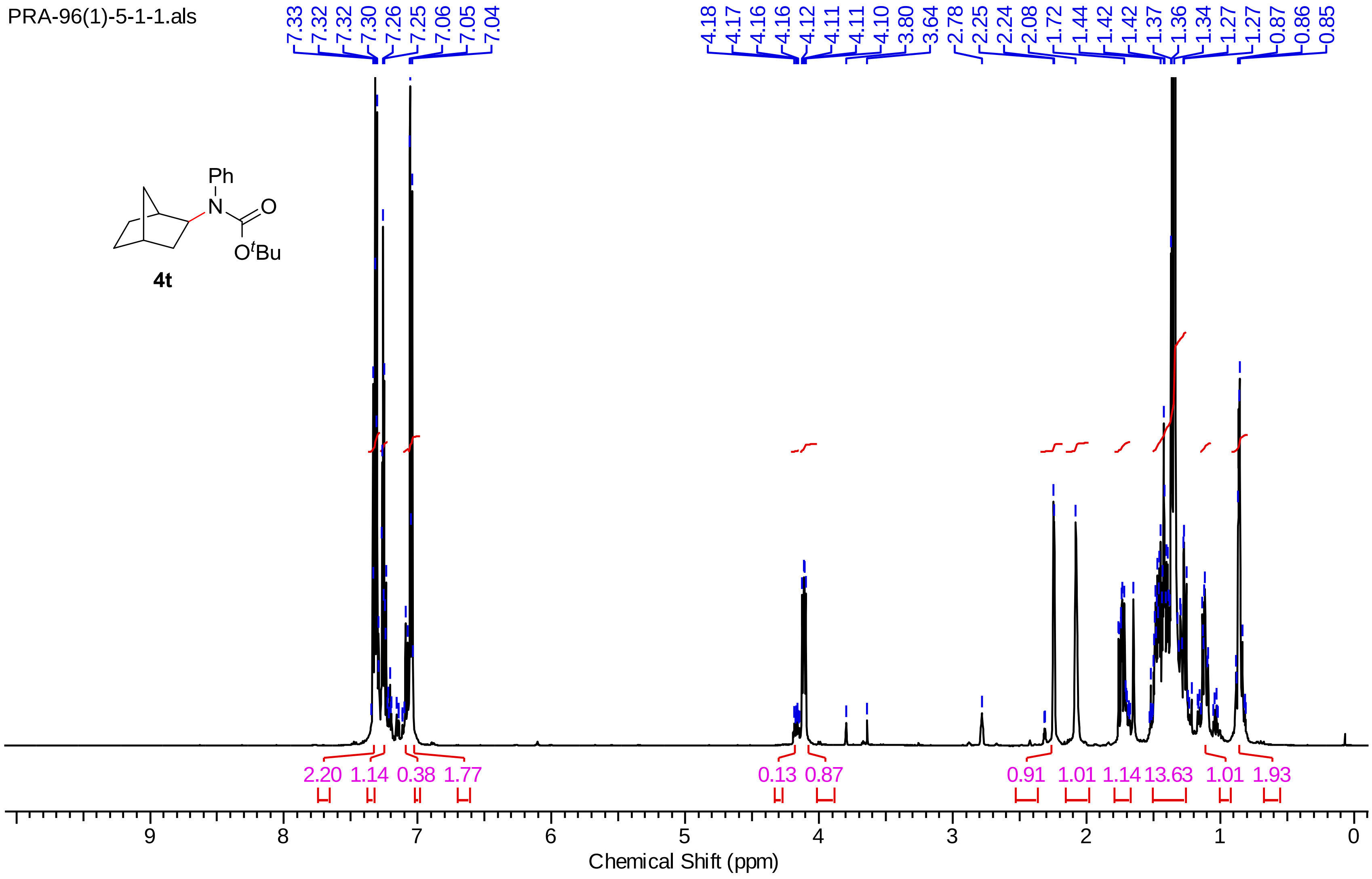
PRA-96(1)-5-1-1.als
Ph
N
O
O^{t}Bu
4t
7.33 7.32 7.32 7.30 7.26 7.25 7.06 7.05 7.04
4.18 4.17 4.16 4.16 4.12 4.11 4.11 4.10 3.80 3.64 2.78 2.25 2.24 2.08 1.72 1.44 1.42 1.42 1.37 1.36 1.34 1.27 1.27 0.87 0.86 0.85
2.20 1.14 0.38 1.77
0.13 0.87
0.91 1.01 1.14 13.63 1.01 1.93
9 8 7 6 5 4 3 2 1 0
Chemical Shift (ppm)

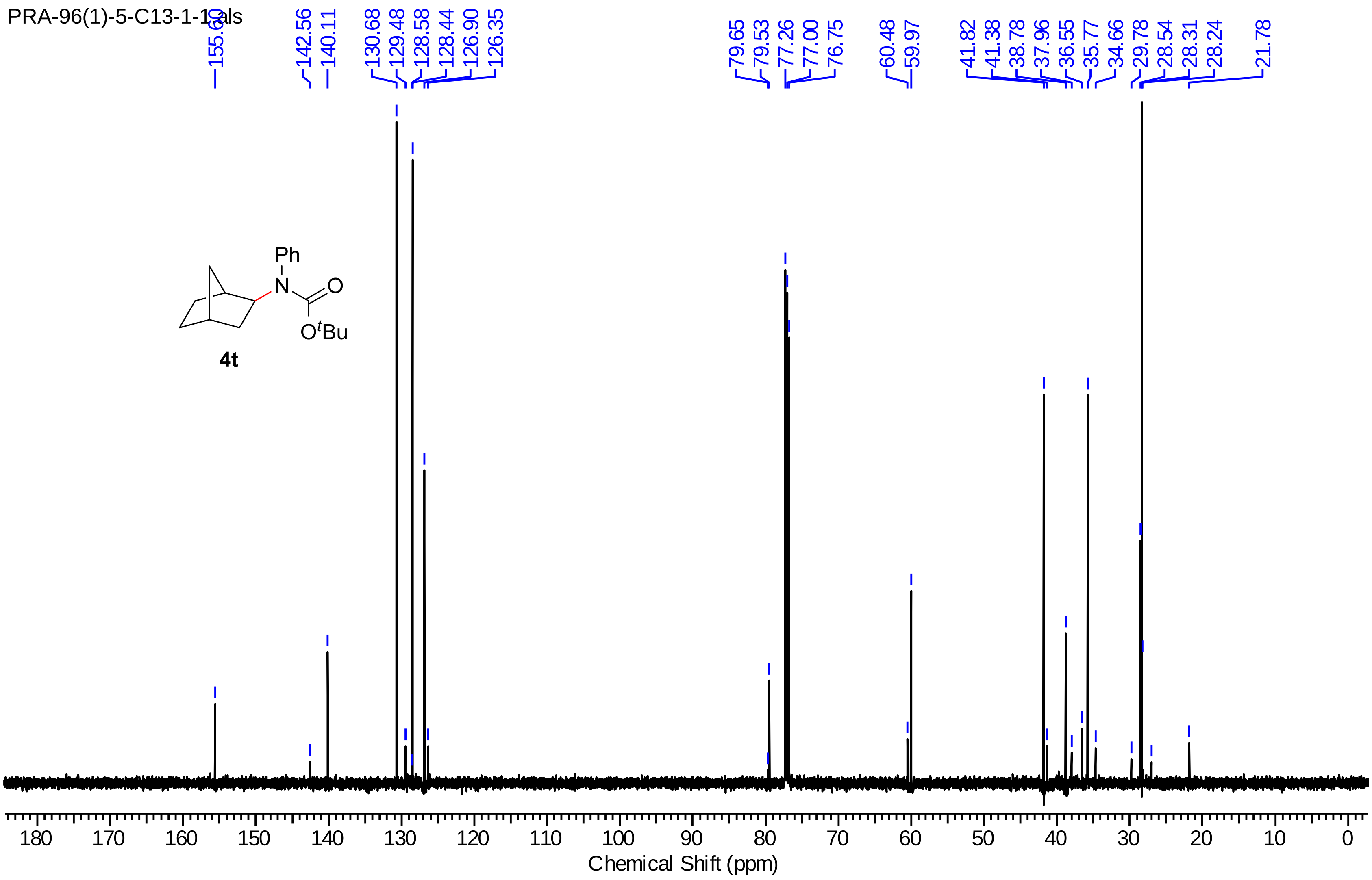
PRA-96(1)-5-C13-1-1.als
155.60
142.56
140.11
130.68
129.48
128.58
128.44
126.90
126.35
79.65
79.53
77.26
77.00
76.75
60.48
59.97
41.82
41.38
38.78
37.96
36.55
35.77
34.66
29.78
28.54
28.31
28.24
21.78
Ph
N
O
O^{t}Bu
4t
Chemical Shift (ppm)

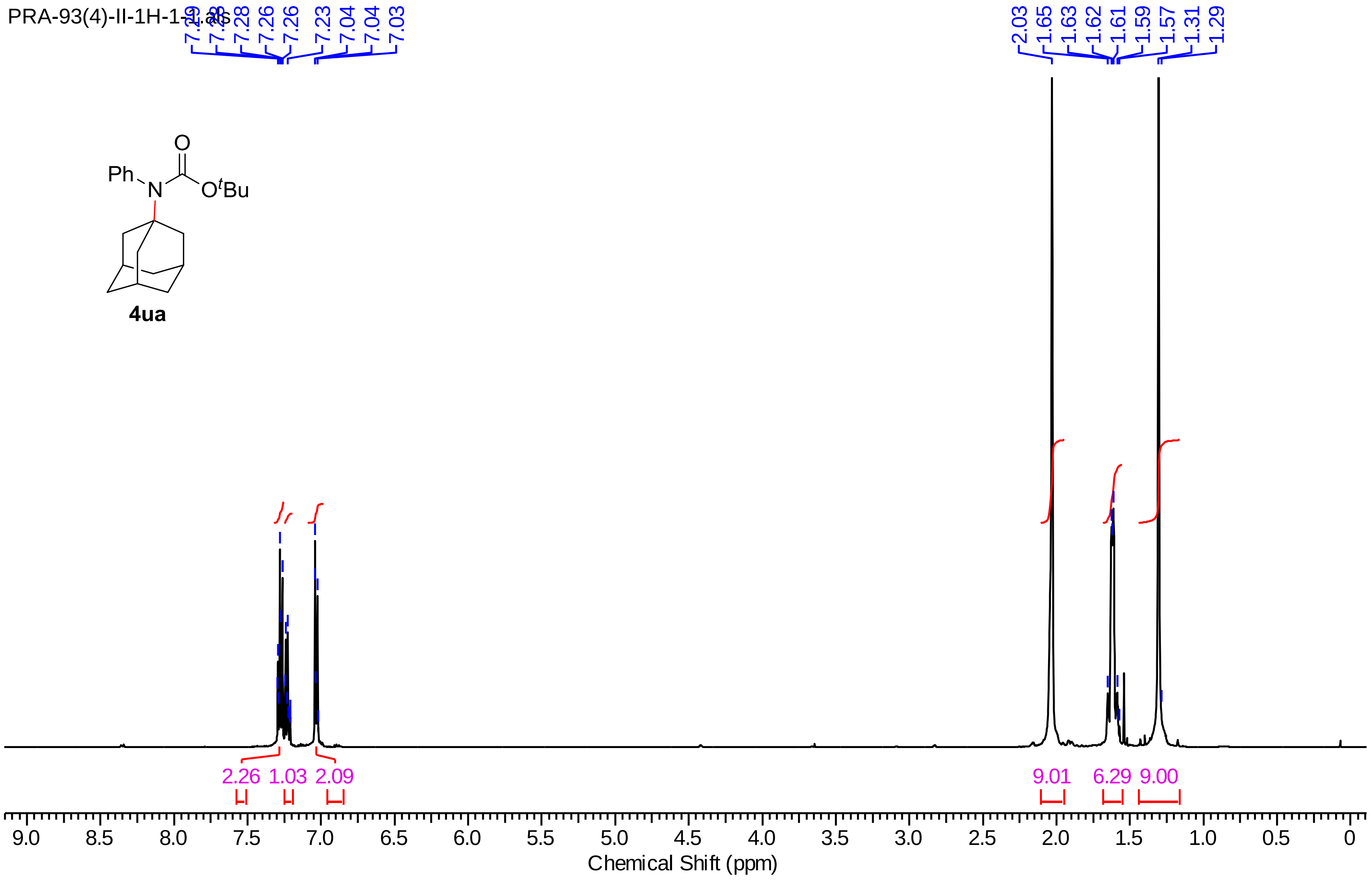
PRA-93(4)-II-1H-1-1.as
7.29
7.28
7.28
7.26
7.26
7.23
7.04
7.04
7.03
2.03
1.65
1.63
1.62
1.61
1.59
1.57
1.31
1.29
Ph
N
O
O^{t}Bu
4ua
2.26
1.03
2.09
9.01
6.29
9.00
9.0
8.5
8.0
7.5
7.0
6.5
6.0
5.5
5.0
4.5
4.0
3.5
3.0
2.5
2.0
1.5
1.0
0.5
0
Chemical Shift (ppm)

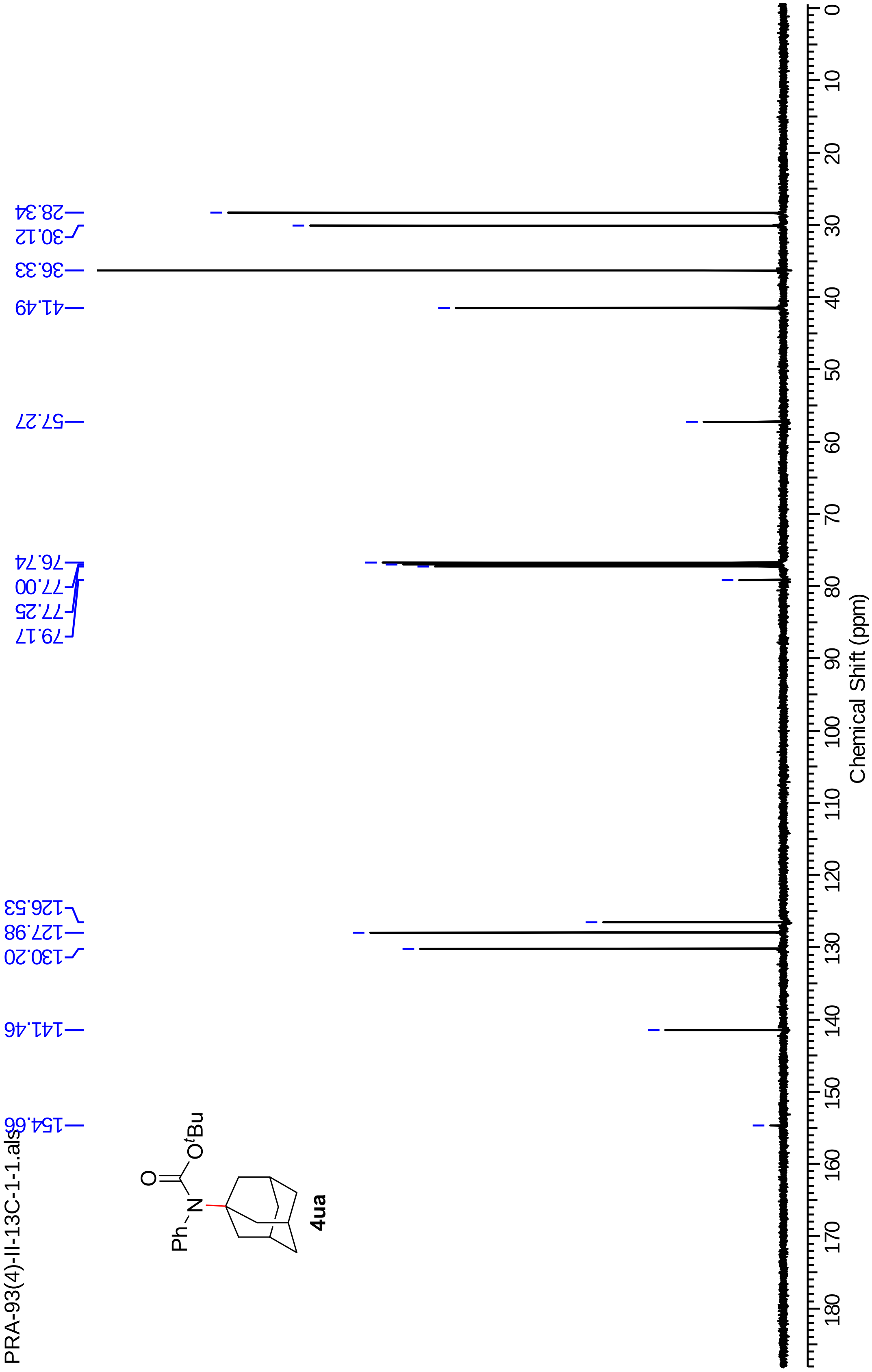
PRA-93(4)-II-13C-1-1.als
154.66
141.46
130.20
127.98
126.53
79.17
77.25
77.00
76.74
57.27
41.49
36.33
30.12
28.34
Ph
N
O
O^{t}Bu
4ua
Chemical Shift (ppm)
0
10
20
30
40
50
60
70
80
90
100
110
120
130
140
150
160
170
180

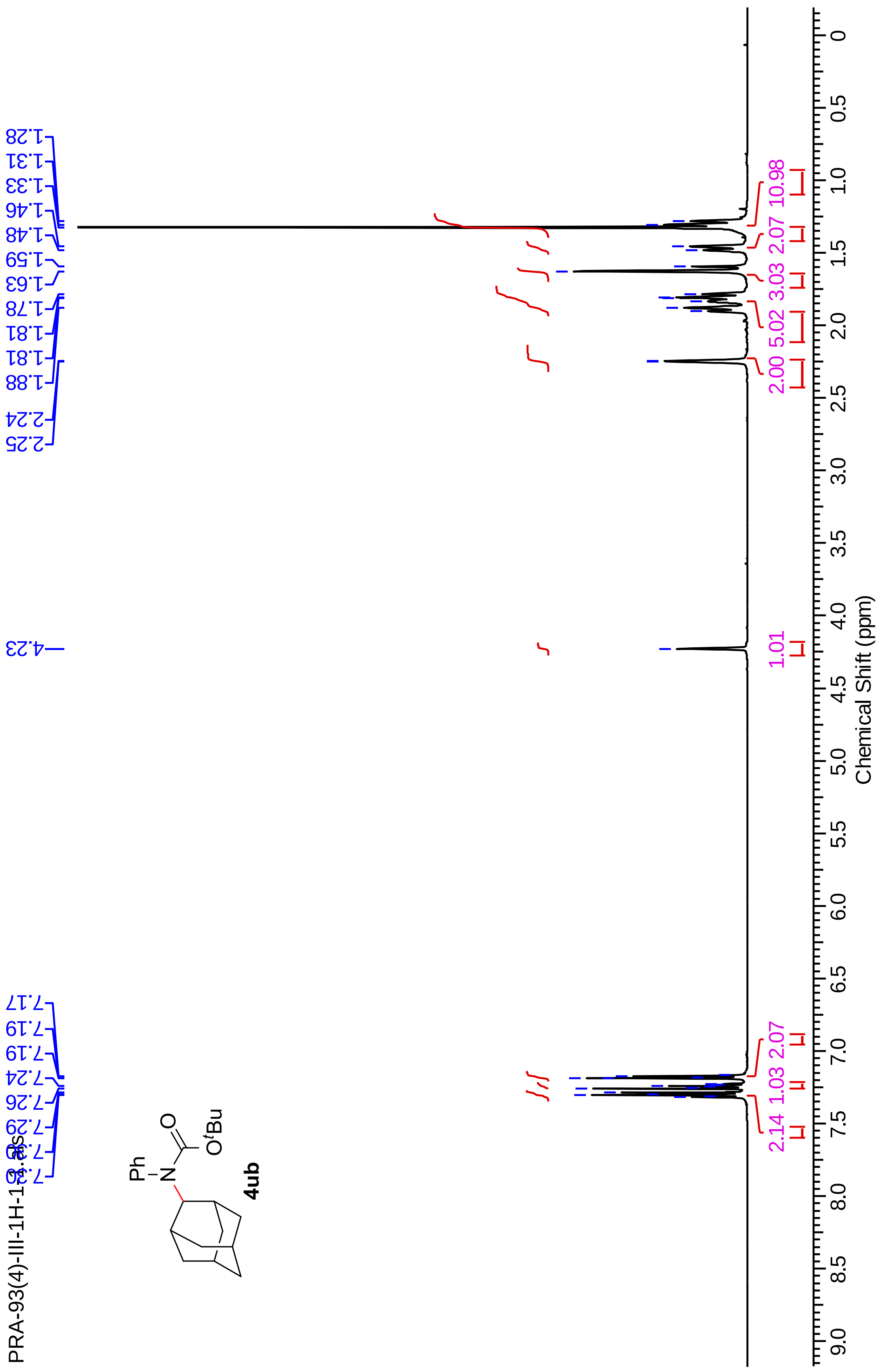

PRA-93(4)-III-1H-1
4ub
Ph
N
O
O^{t}Bu
7.30
7.30
7.29
7.26
7.24
7.19
7.19
7.17
4.23
2.25
2.24
1.88
1.81
1.81
1.78
1.63
1.59
1.48
1.46
1.33
1.31
1.28
2.14
1.03
2.07
1.01
2.00
5.02
3.03
2.07
10.98
Chemical Shift (ppm)
9.0
8.5
8.0
7.5
7.0
6.5
6.0
5.5
5.0
4.5
4.0
3.5
3.0
2.5
2.0
1.5
1.0
0.5
0

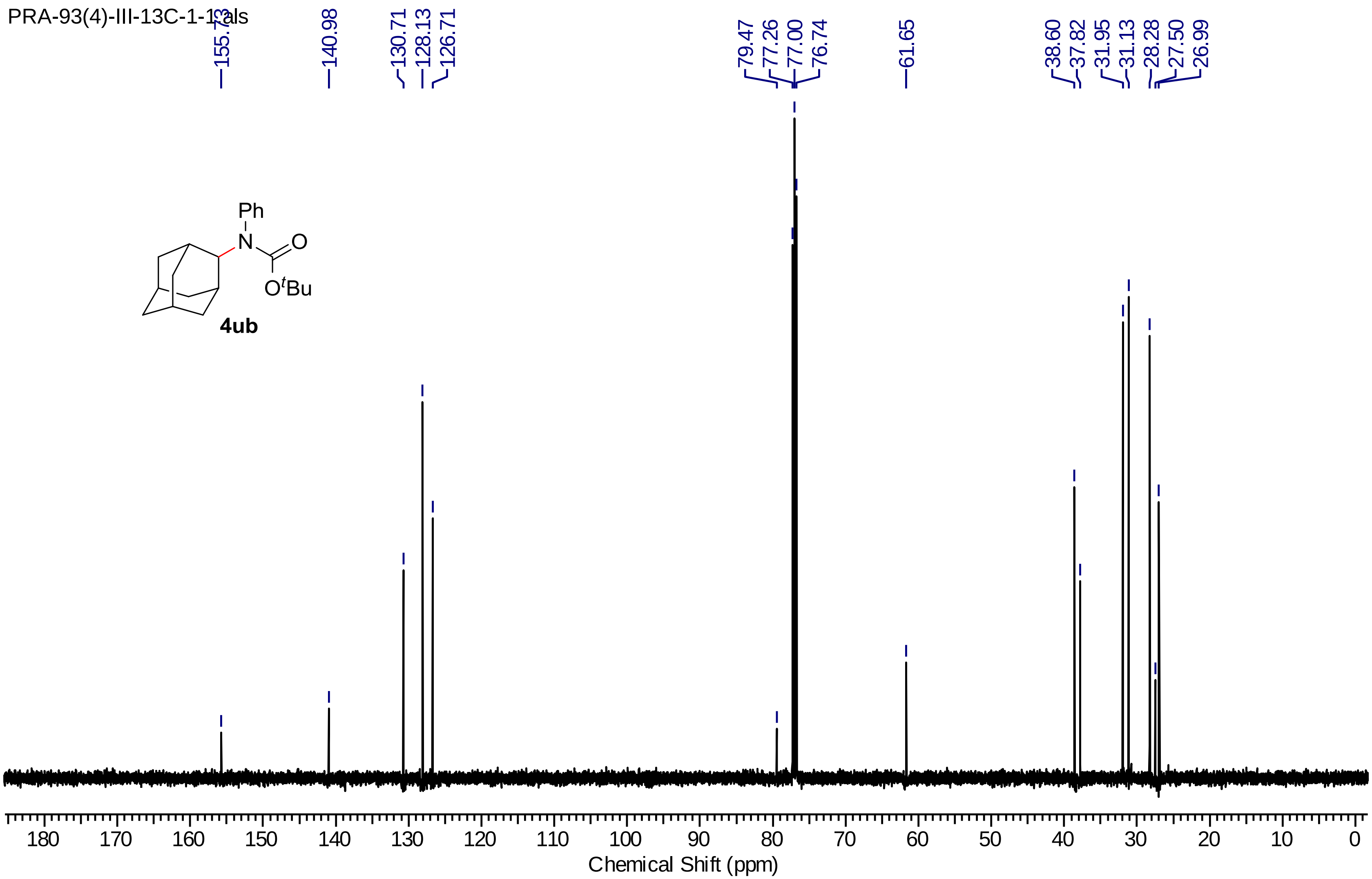
PRA-93(4)-III-13C-1-1.als
155.73
140.98
130.71
128.13
126.71
79.47
77.26
77.00
76.74
61.65
38.60
37.82
31.95
31.13
28.28
27.50
26.99
Ph
N
O
O^{t}Bu
4ub
180 170 160 150 140 130 120 110 100 90 80 70 60 50 40 30 20 10 0
Chemical Shift (ppm)

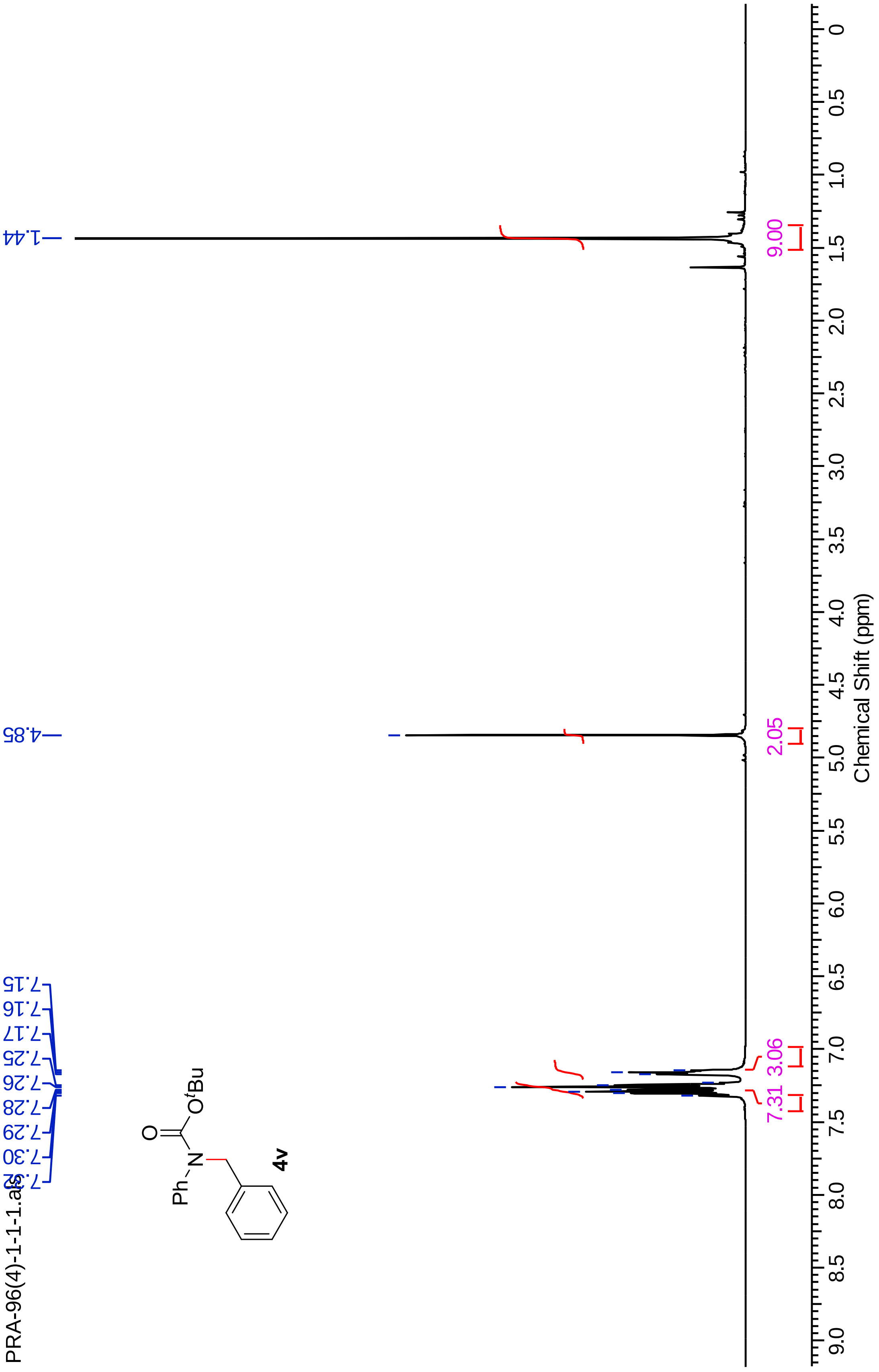
PRA-96(4)-1-1-1.als
7.32
7.30
7.29
7.28
7.26
7.25
7.17
7.16
7.15
4.85
1.44
4v
Ph
N
O
O^tBu
7.31
3.06
2.05
9.00
Chemical Shift (ppm)
9.0
8.5
8.0
7.5
7.0
6.5
6.0
5.5
5.0
4.5
4.0
3.5
3.0
2.5
2.0
1.5
1.0
0.5
0

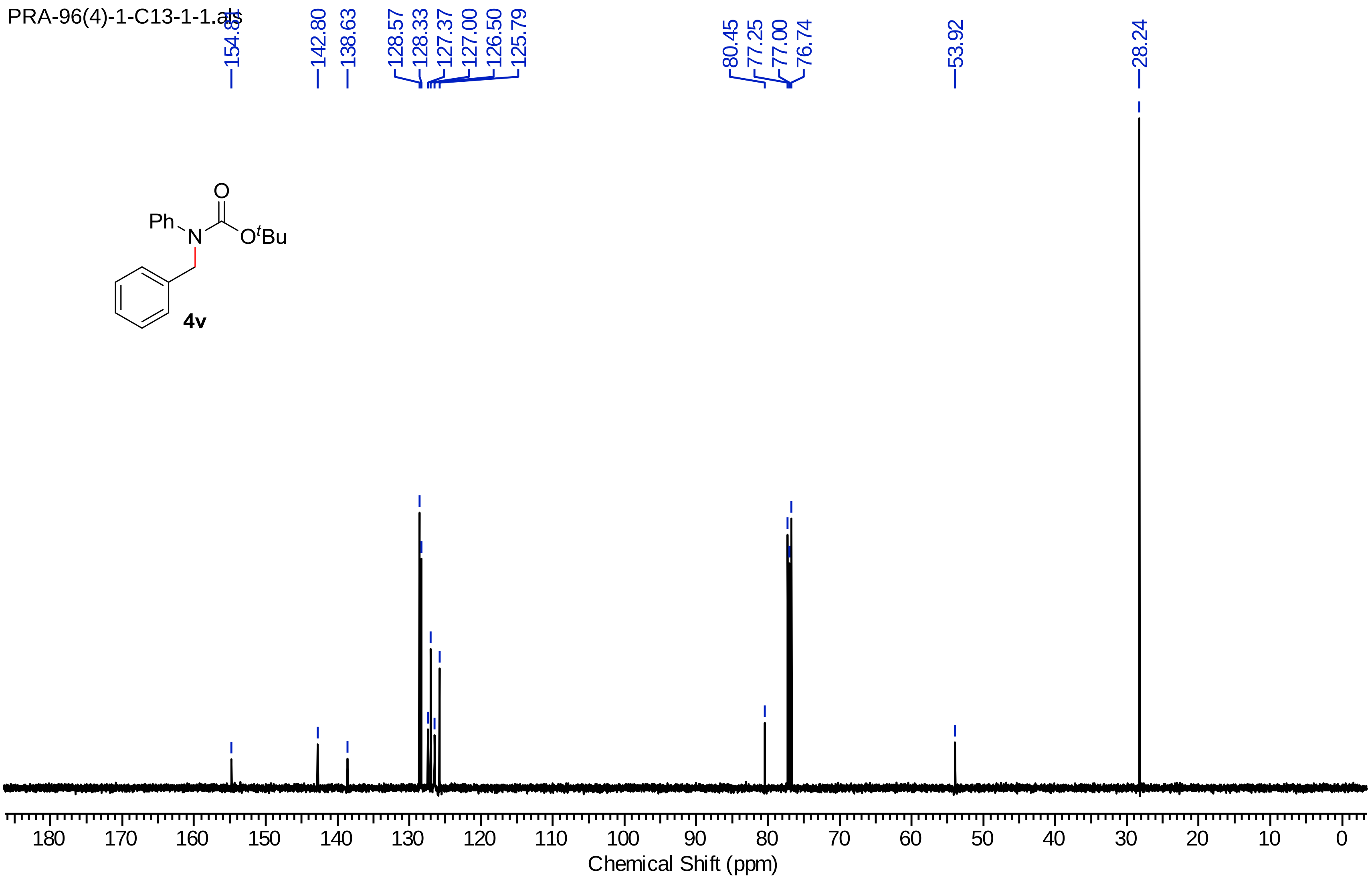
PRA-96(4)-1-C13-1-1.als
154.81
142.80
138.63
128.57
128.33
127.37
127.00
126.50
125.79
80.45
77.25
77.00
76.74
53.92
28.24
Ph
N
O
O^{t}Bu
4v
180 170 160 150 140 130 120 110 100 90 80 70 60 50 40 30 20 10 0
Chemical Shift (ppm)

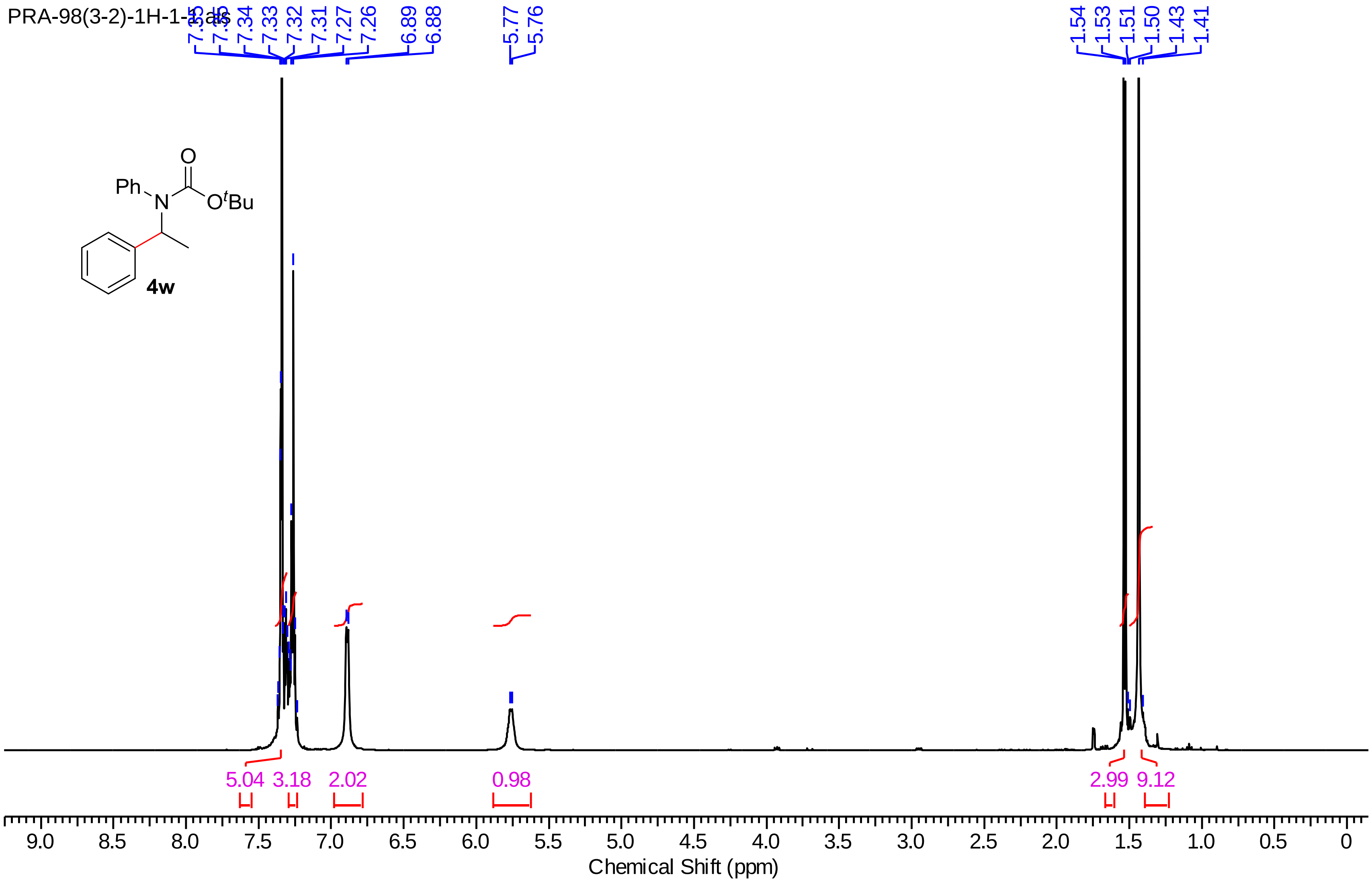

PRA-98(3-2)-1H-1-1.als
7.35
[illegible]
7.34
7.33
7.32
7.31
7.27
7.26
6.89
6.88
5.77
5.76
1.54
1.53
1.51
1.50
1.43
1.41
Ph
N
O
O^{t}Bu
4w
5.04
3.18
2.02
0.98
2.99
9.12
9.0 8.5 8.0 7.5 7.0 6.5 6.0 5.5 5.0 4.5 4.0 3.5 3.0 2.5 2.0 1.5 1.0 0.5 0
Chemical Shift (ppm)

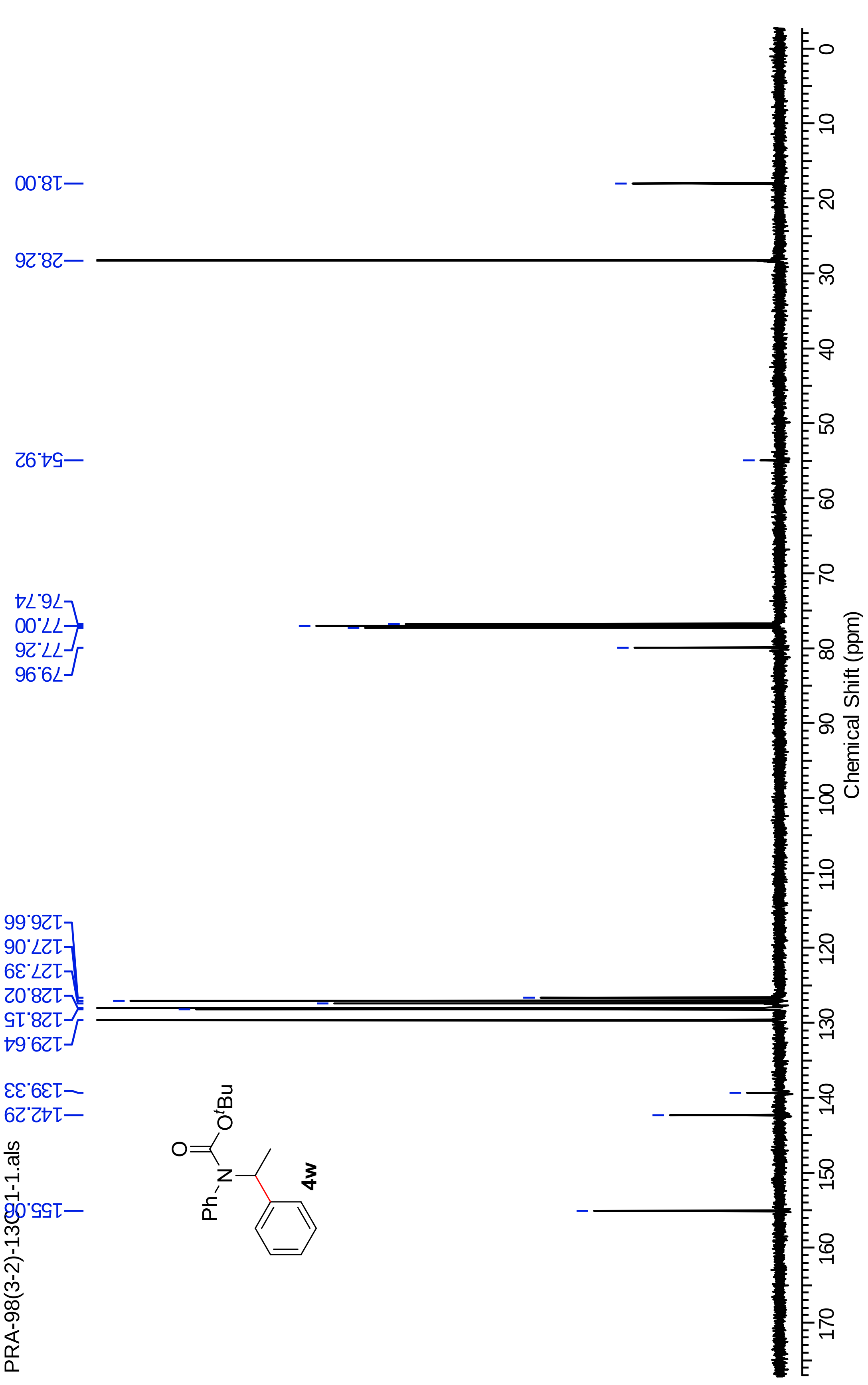
PRA-98(3-2)-13C1-1.als
155.08
142.29
139.33
129.64
128.15
128.02
127.39
127.06
126.66
79.96
77.26
77.00
76.74
54.92
28.26
18.00
Ph
N
O
O^tBu
4w
Chemical Shift (ppm)

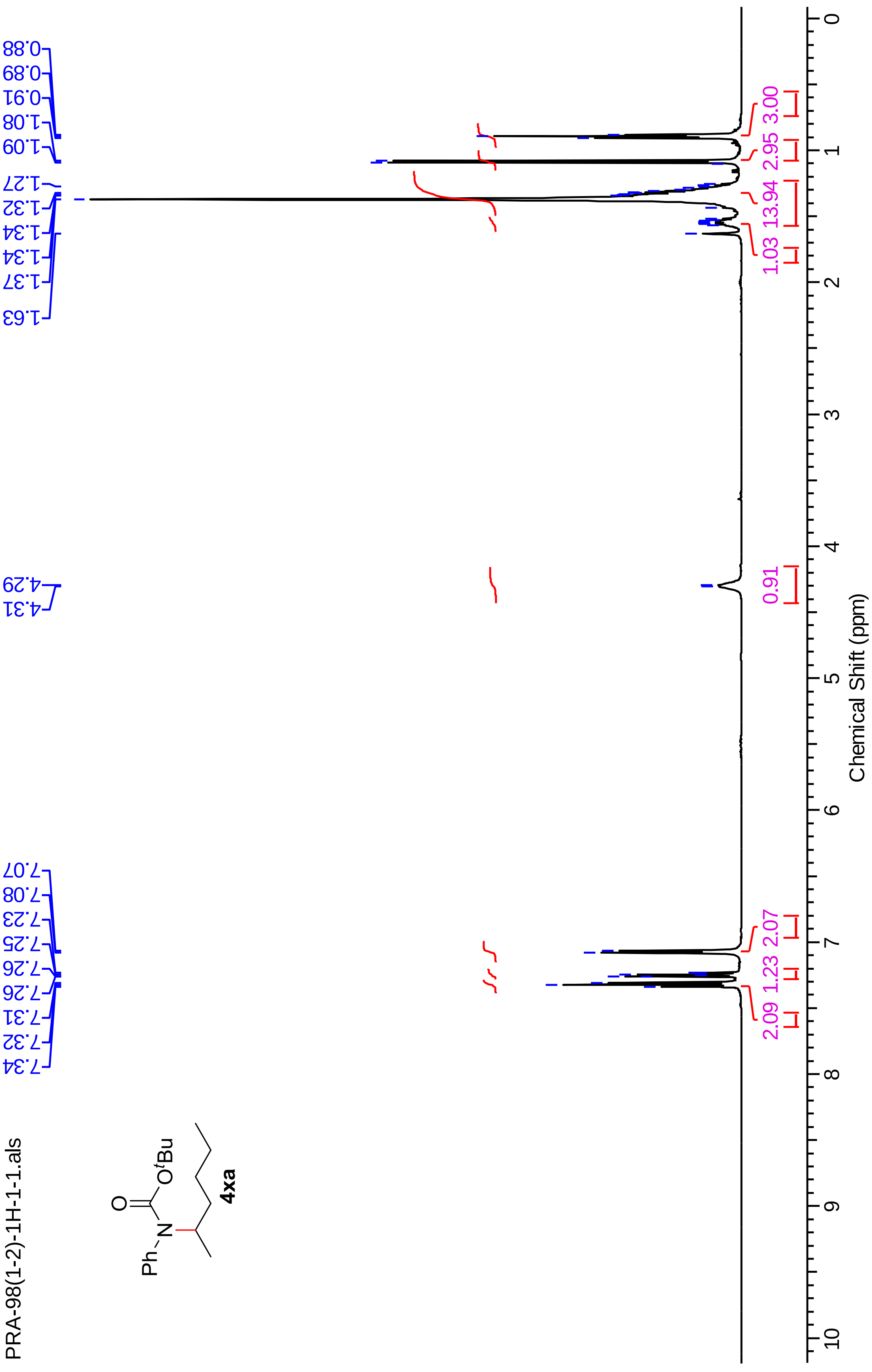
PRA-98(1-2)-1H-1-1.als
Ph
N
O
O^{t}Bu
4xa
7.34
7.32
7.31
7.26
7.26
7.25
7.23
7.08
7.07
4.31
4.29
1.63
1.37
1.34
1.34
1.32
1.27
1.09
1.08
0.91
0.89
0.88
2.09
1.23
2.07
0.91
1.03
13.94
2.95
3.00
Chemical Shift (ppm)

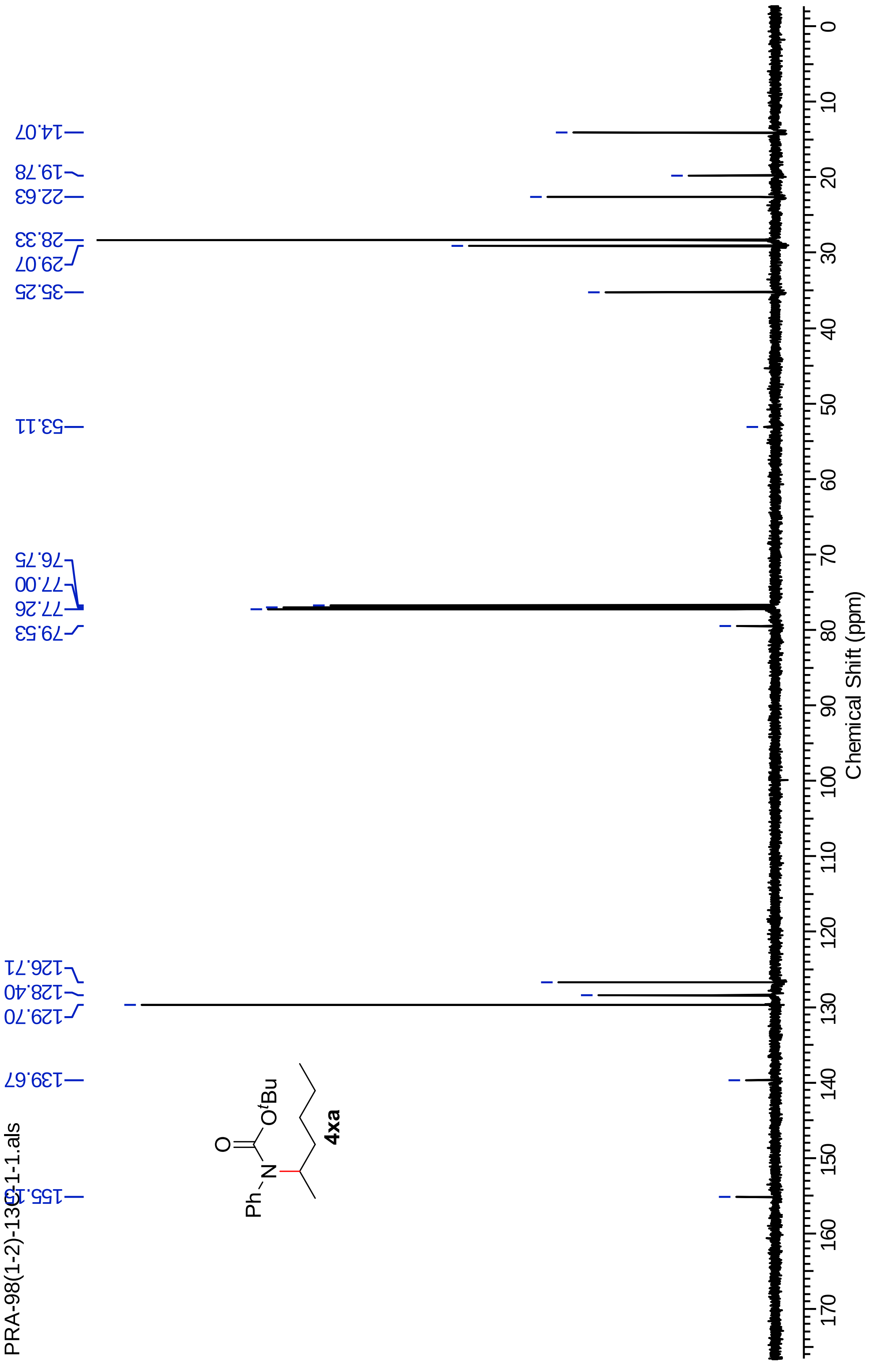

PRA-98(1-2)-13C-1-1.als
155.15
139.67
129.70
128.40
126.71
79.53
77.26
77.00
76.75
53.11
35.25
29.07
28.33
22.63
19.78
14.07
Ph
N
O
O^{t}Bu
4xa
Chemical Shift (ppm)

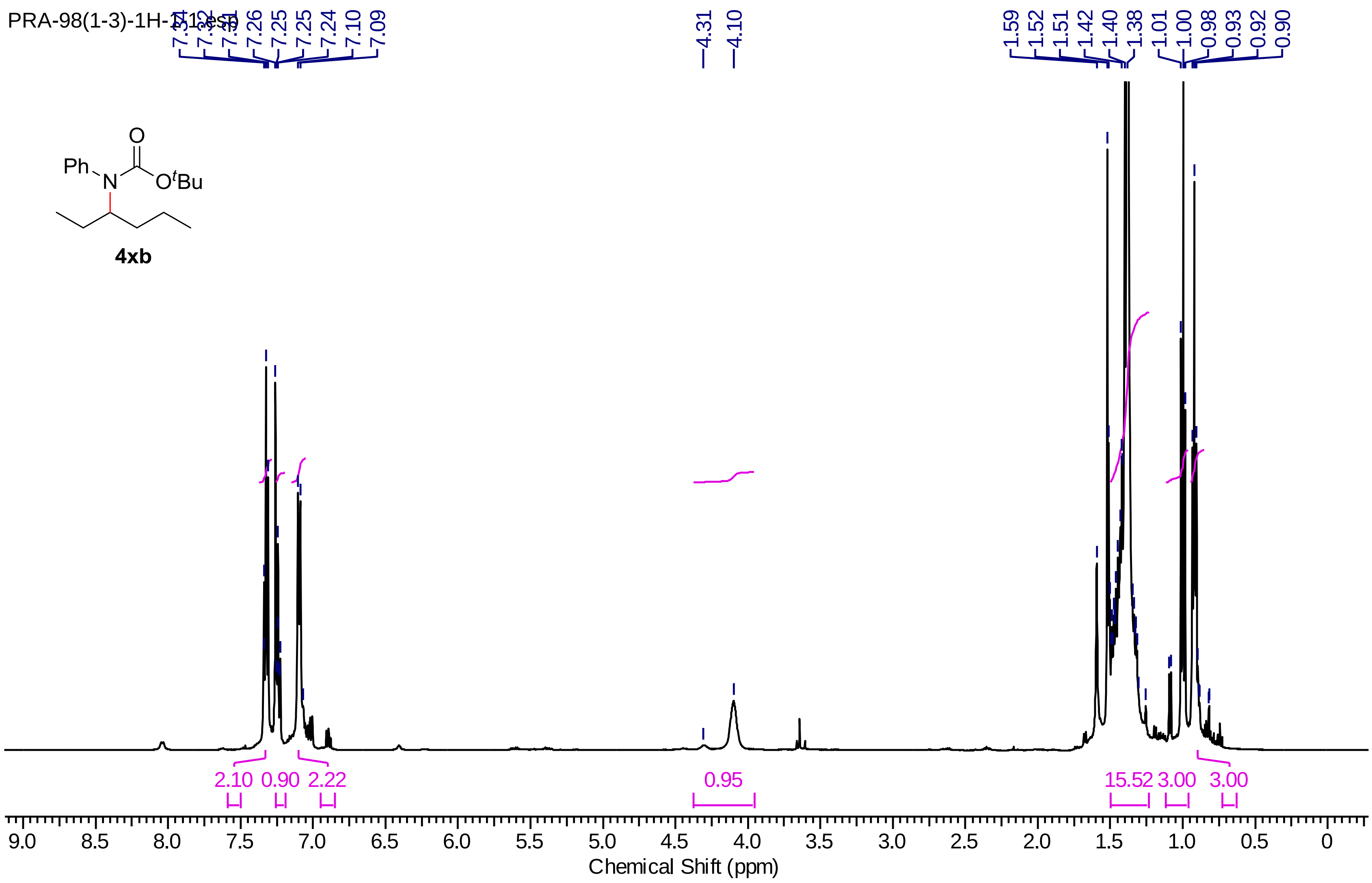
PRA-98(1-3)-1H-11.esp
7.34 7.32 7.31 7.26 7.25 7.25 7.24 7.10 7.09
4.31 4.10
1.59 1.52 1.51 1.42 1.40 1.38 1.01 1.00 0.98 0.93 0.92 0.90
Ph N O O^{t}Bu
4xb
2.10 0.90 2.22 0.95 15.52 3.00 3.00
Chemical Shift (ppm)

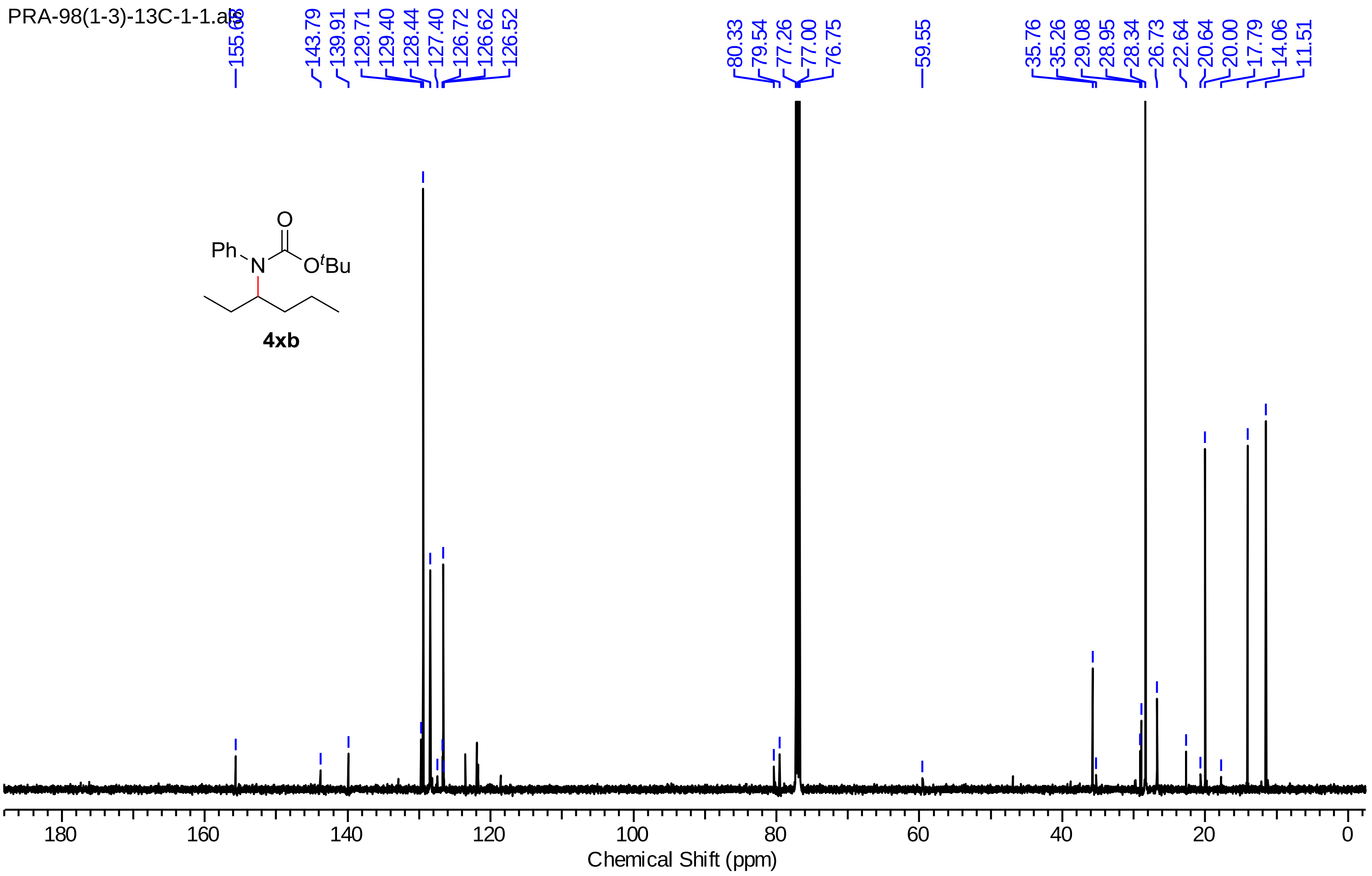
PRA-98(1-3)-13C-1-1.als
155.68
143.79
139.91
129.71
129.40
128.44
127.40
126.72
126.62
126.52
80.33
79.54
77.26
77.00
76.75
59.55
35.76
35.26
29.08
28.95
28.34
26.73
22.64
20.64
20.00
17.79
14.06
11.51
Ph
N
O
O^{t}Bu
4xb
180
160
140
120
100
80
60
40
20
0
Chemical Shift (ppm)

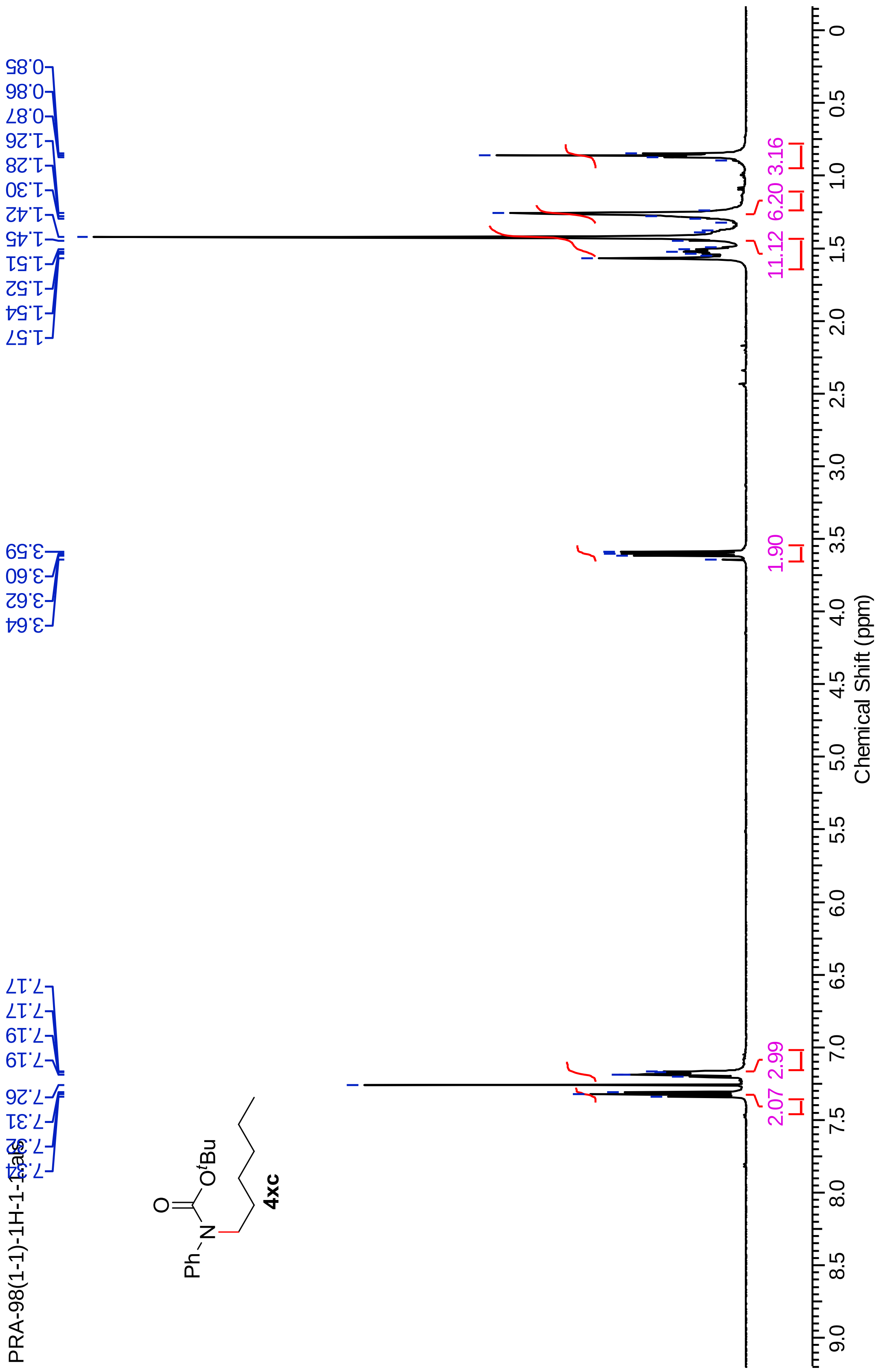
PRA-98(1-1)-1H-1-1.als
7.34
7.32
7.31
7.26
7.19
7.19
7.17
7.17
3.64
3.62
3.60
3.59
1.57
1.54
1.52
1.51
1.45
1.42
1.30
1.28
1.26
0.87
0.86
0.85
Ph
N
O
O
tBu
4xc
2.07
2.99
1.90
11.12
6.20
3.16
Chemical Shift (ppm)

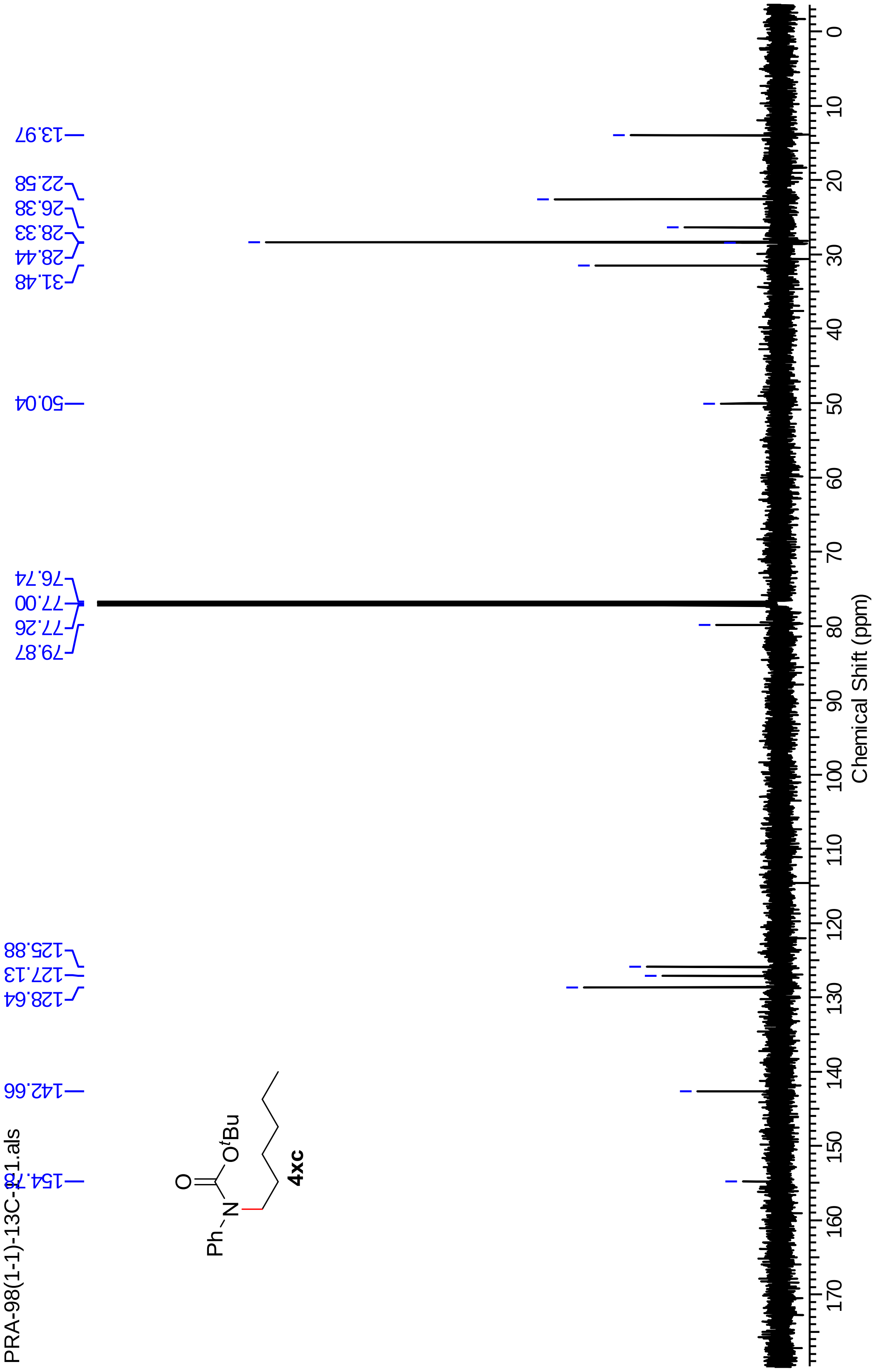

PRA-98(1-1)-13C-81.als
154.78
142.66
128.64
127.13
125.88
79.87
77.26
77.00
76.74
50.04
31.48
28.44
28.33
26.38
22.58
13.97
Ph
N
O
O^tBu
4xc
170 160 150 140 130 120 110 100 90 80 70 60 50 40 30 20 10 0
Chemical Shift (ppm)

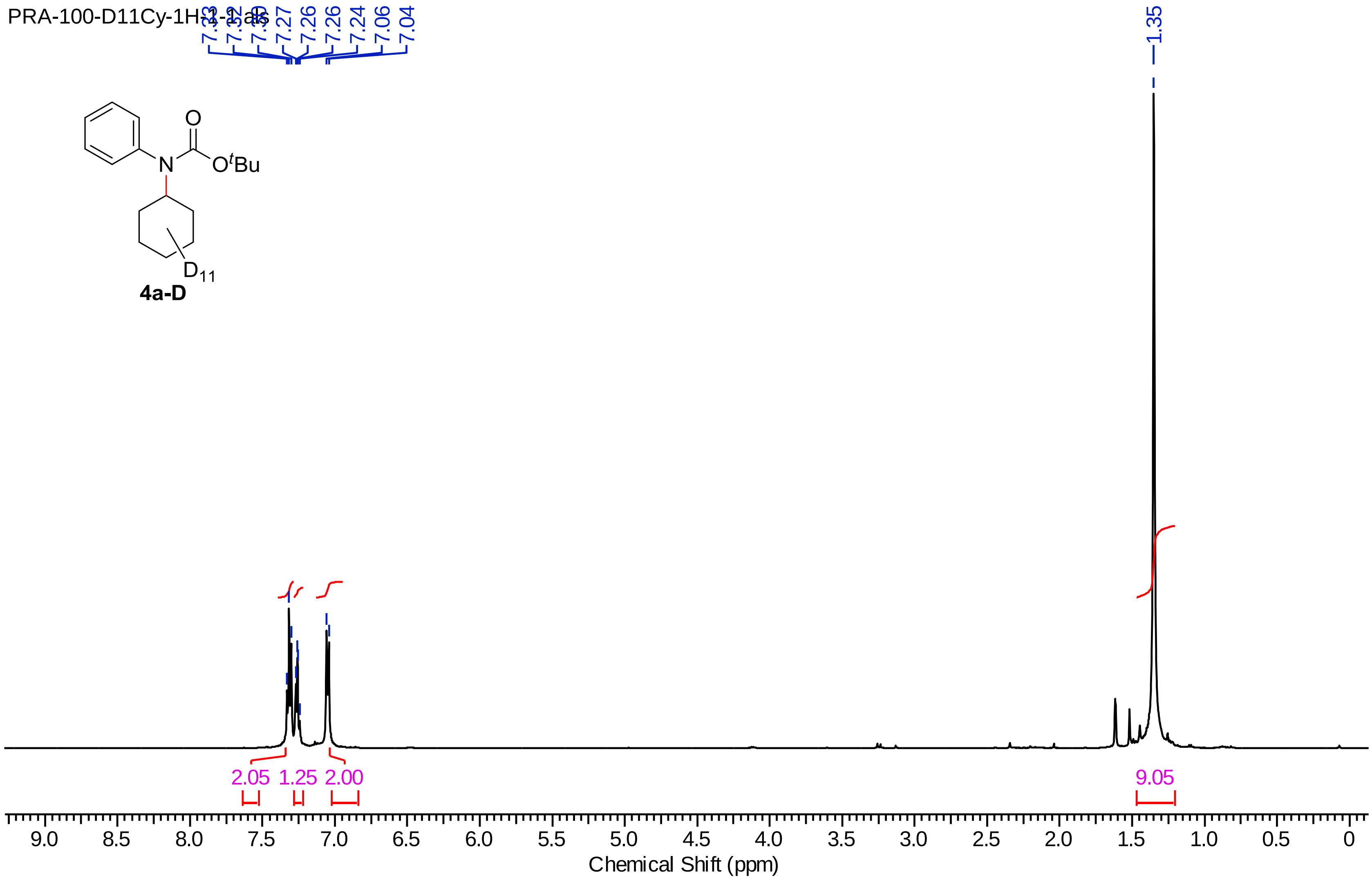

PRA-100-D11Cy-1H
7.33
7.32
7.30
7.27
7.26
7.26
7.24
7.06
7.04
1.35
O
N
O^tBu
D11
4a-D
2.05
1.25
2.00
9.05
9.0
8.5
8.0
7.5
7.0
6.5
6.0
5.5
5.0
4.5
4.0
3.5
3.0
2.5
2.0
1.5
1.0
0.5
0
Chemical Shift (ppm)

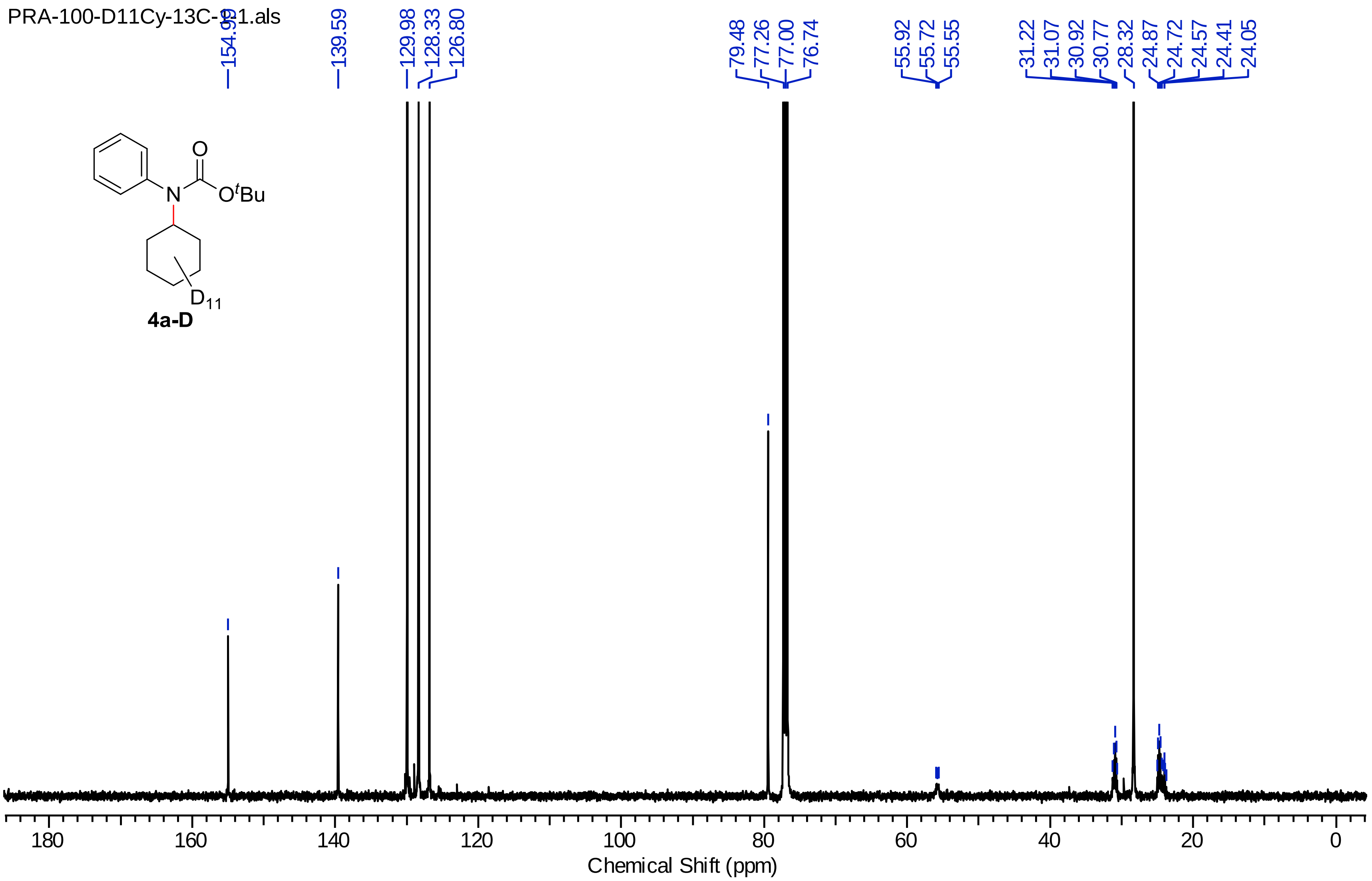
PRA-100-D11Cy-13C-01.als
154.99
139.59
129.98
128.33
126.80
79.48
77.26
77.00
76.74
55.92
55.72
55.55
31.22
31.07
30.92
30.77
28.32
24.87
24.72
24.57
24.41
24.05
O
N
O^{t}Bu
D$_{11}$
4a-D
180
160
140
120
100
80
60
40
20
0
Chemical Shift (ppm)

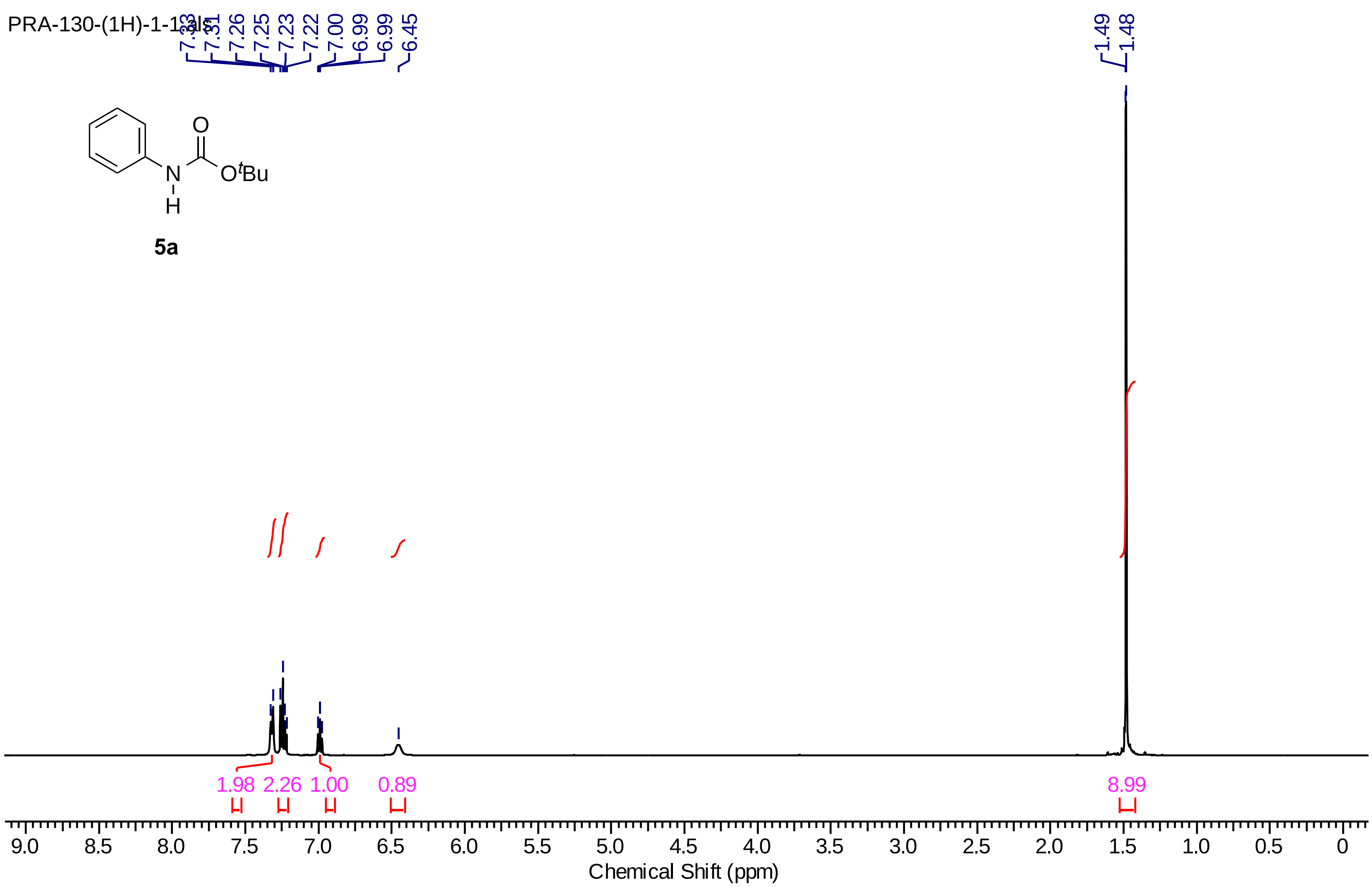
PRA-130-(1H)-1-1.als
7.33
7.31
7.26
7.25
7.23
7.22
7.00
6.99
6.99
6.45
1.49
1.48
O
N
H
O^{t}Bu
5a
1.98
2.26
1.00
0.89
8.99
9.0
8.5
8.0
7.5
7.0
6.5
6.0
5.5
5.0
4.5
4.0
3.5
3.0
2.5
2.0
1.5
1.0
0.5
0
Chemical Shift (ppm)

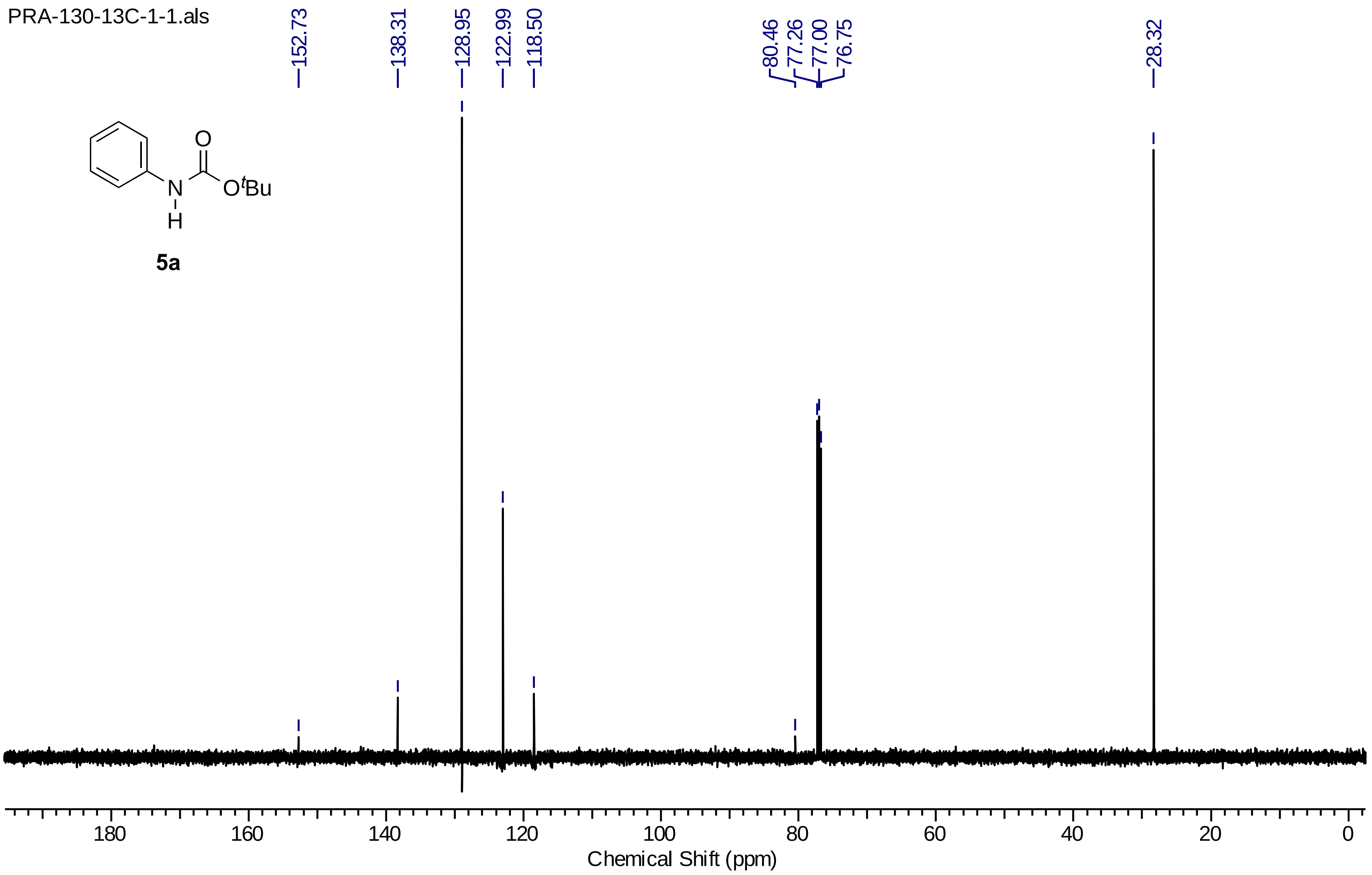

PRA-130-13C-1-1.als
152.73
138.31
128.95
122.99
118.50
80.46
77.26
77.00
76.75
28.32
O
N
H
O^tBu
5a
180
160
140
120
100
80
60
40
20
0
Chemical Shift (ppm)

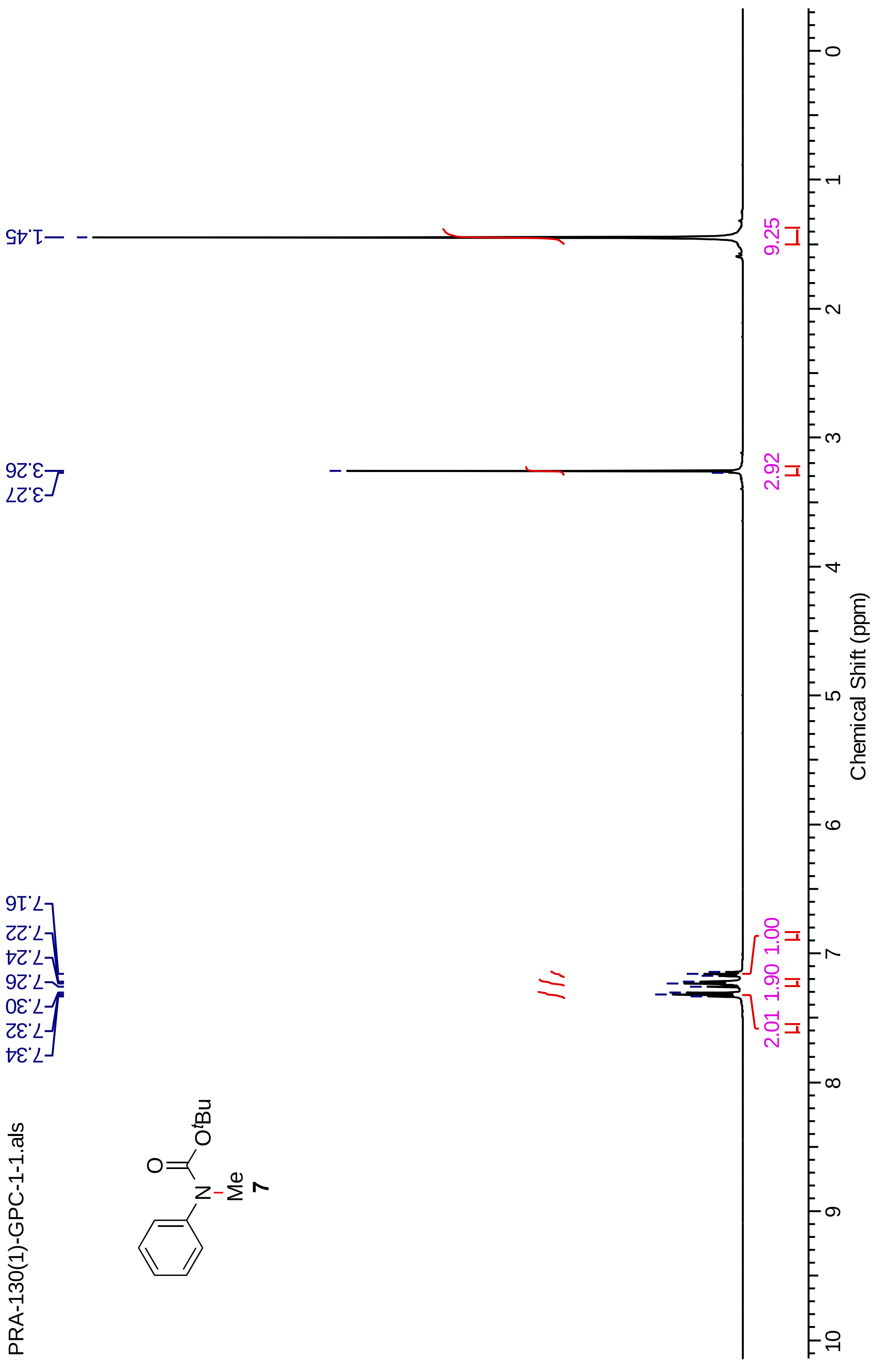

PRA-130(1)-GPC-1-1.als
O
N
Me
O^{t}Bu
7
7.34
7.32
7.30
7.26
7.24
7.22
7.16
3.27
3.26
1.45
2.01
1.90
1.00
2.92
9.25
Chemical Shift (ppm)
10
9
8
7
6
5
4
3
2
1
0

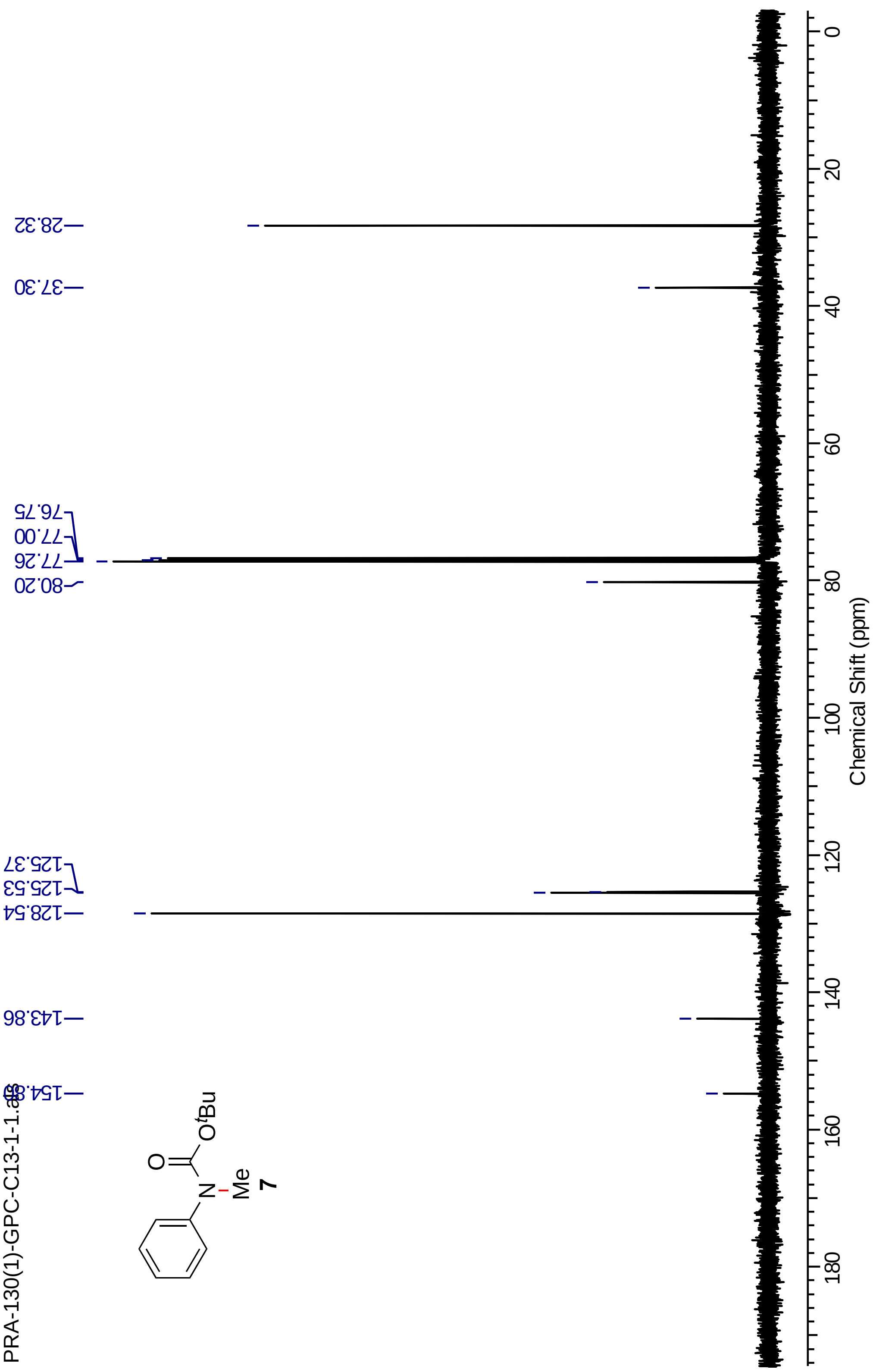
PRA-130(1)-GPC-C13-1-1.als
154.85
143.86
128.54
125.53
125.37
80.20
77.26
77.00
76.75
37.30
28.32
O
N
Me
O^{t}Bu
7
0
20
40
60
80
100
120
140
160
180
Chemical Shift (ppm)

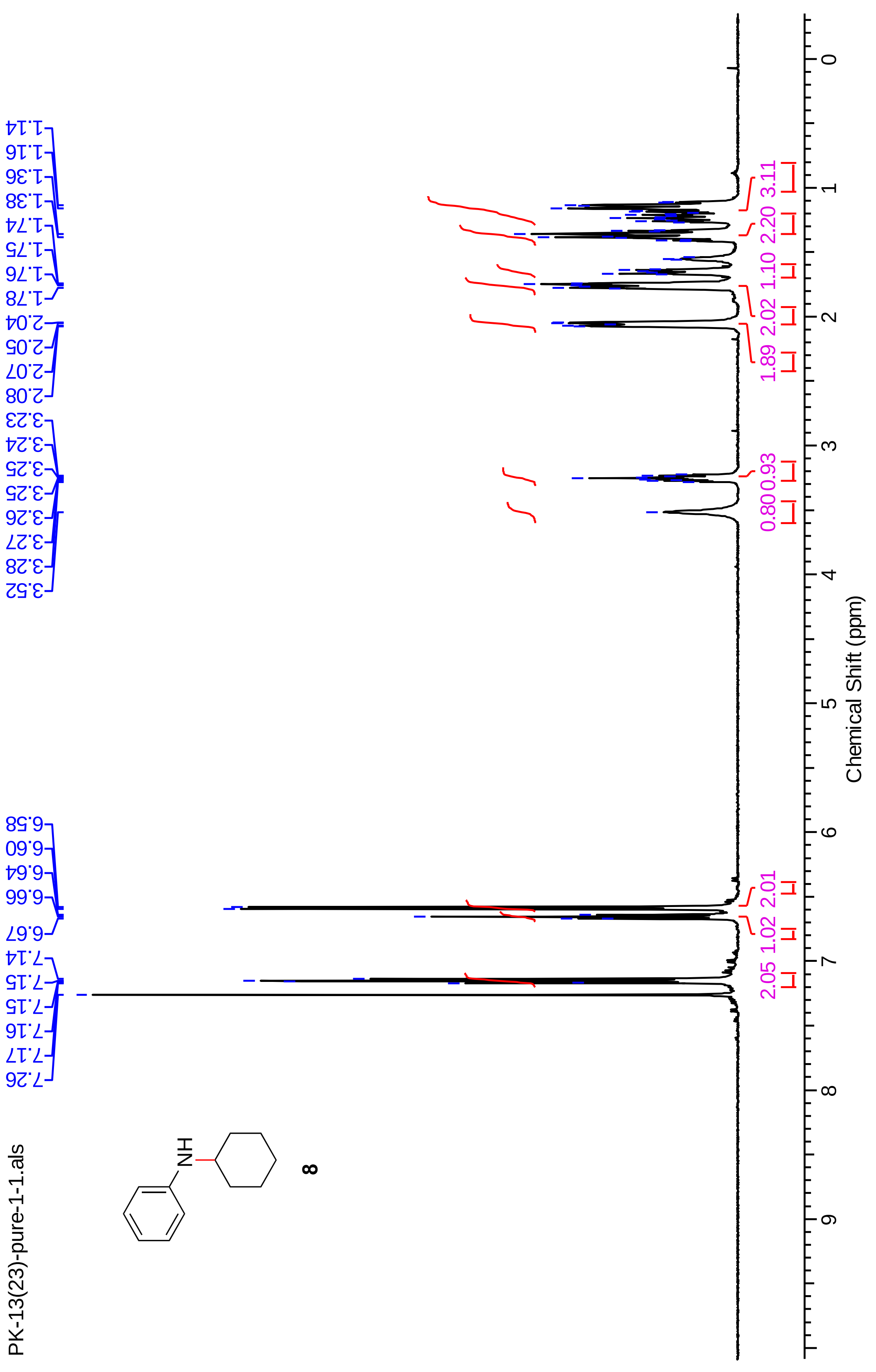

PK-13(23)-pure-1-1.als
NH
8
7.26
7.17
7.16
7.15
7.15
7.14
6.67
6.66
6.64
6.60
6.58
3.52
3.28
3.27
3.26
3.25
3.25
3.24
3.23
2.08
2.07
2.05
2.04
1.78
1.76
1.75
1.74
1.38
1.36
1.16
1.14
2.05 1.02 2.01
0.80 0.93
1.89 2.02 1.10 2.20 3.11
9 8 7 6 5 4 3 2 1 0
Chemical Shift (ppm)

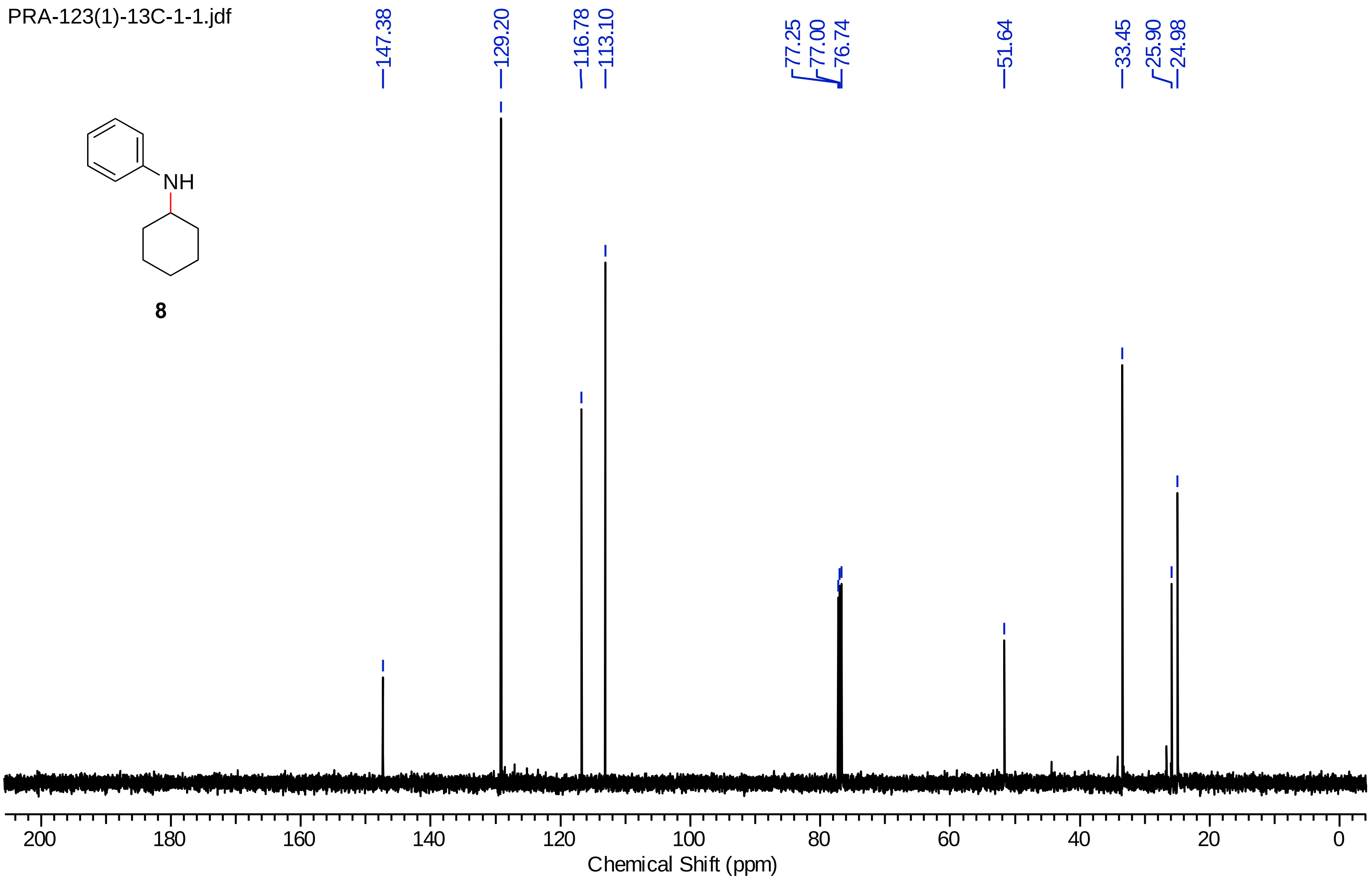

PRA-123(1)-13C-1-1.jdf
NH
8
147.38
129.20
116.78
113.10
77.25
77.00
76.74
51.64
33.45
25.90
24.98
200
180
160
140
120
100
80
60
40
20
0
Chemical Shift (ppm)
